# Modularization of the type II secretion gene cluster from *Xanthomonas euvesicatoria* facilitates the identification of a structurally conserved XpsCLM assembly platform complex

**DOI:** 10.1371/journal.ppat.1013008

**Published:** 2025-04-09

**Authors:** Samuel Goll, Patrick Martin, Sylvestre Marillonnet, Daniela Büttner

**Affiliations:** 1 Department of Genetics, Institute for Biology, Martin-Luther University Halle-Wittenberg, Halle (Saale), Germany; 2 Leibniz Institute for Plant Biochemistry, Halle (Saale), Germany; The Ohio State University, UNITED STATES OF AMERICA

## Abstract

Many bacterial pathogens depend on a type II secretion (T2S) system to secrete virulence factors from the periplasm into the extracellular milieu. T2S systems consist of an outer membrane secretin channel, a periplasmic pseudopilus and an inner membrane-associated assembly platform including a cytoplasmic ATPase. The components of T2S systems are often conserved in different bacterial species, however, the architecture of the assembly platform is largely unknown. Here, we analysed predicted assembly platform components of the Xps-T2S system from the plant-pathogenic bacterium *Xanthomonas euvesicatoria*. To facilitate these studies, we generated a modular *xps-*T2S gene cluster by Golden Gate assembly of single promoter and gene fragments. The modular design allowed the efficient deletion and replacement of T2S genes and the insertion of reporter fusions. Mutant approaches as well as interaction and crosslinking studies showed that the predicted assembly platform components XpsC, XpsL and XpsM form a trimeric complex which is essential for T2S and associates with the cytoplasmic ATPase XpsE and the secretin XpsD. Structural modeling revealed a similar trimeric architecture of XpsCLM homologs from *Pseudomonas, Vibrio* and *Klebsiella* species, despite overall low amino acid sequence similarities. In *X. euvesicatoria,* crosslinking and fluorescence microscopy studies showed that the formation of the XpsCLM complex is independent of the secretin and *vice versa*, suggesting that the assembly of the T2S system is a dynamic process which involves the association of preformed subcomplexes.

## Author summary

The plant-pathogenic bacterium *Xanthomonas euvesicatoria* secretes degradative enzymes via a type II secretion (T2S) system into the extracellular milieu to facilitate infection of its host plants, pepper and tomato. T2S systems are composed of an outer membrane secretin channel, a periplasmic pseudopilus and an inner membrane-associated assembly platform with a cytoplasmic ATPase. The architecture of the assembly platform, which is required for substrate recognition in the periplasm, is still largely uncharacterized. In the present study, we used a modular gene cluster approach to study the assembly platform components XpsC, XpsL and XpsM from *X. euvesicatoria.* We show that they form a trimeric complex, which associates with the cytoplasmic ATPase XpsE and the secretin XpsD. Structural modeling suggests the presence of similar trimeric complexes in related bacteria, despite low amino acid sequence similarity of the individual proteins. Additionally, our studies indicate that the formation of the XpsCLM complex is independent of the secretin, highlighting the dynamic nature of T2S assembly, which likely involves the association of preformed subcomplexes. This research enhances our understanding of bacterial T2S systems and their contribution to the secretion of virulence factors.

## Introduction

Bacterial type II secretion (T2S) systems deliver folded proteins across the outer membrane (OM) into the extracellular milieu and have been identified as virulence factors in many important human (e.g., *Klebsiella pneumoniae*, *Legionella pneumophila*, *Pseudomonas aeruginosa* and *Vibrio cholerae*) and plant pathogens (e.g., *Dickeya dadantii*, *Erwinia amylovora*, *Xanthomonas campestris* and *Xylella fastidiosa*) [1–3]. T2S substrates include degradative enzymes, toxins and proteins involved in bacterial adhesion, biofilm formation and nutrient acquisition [4]. One prominent example for a virulence-associated T2S substrate is cholera toxin from *V. cholerae* [5].

T2S-dependent protein delivery into the extracellular milieu is a two-step process which involves protein secretion across the inner membrane (IM) by the general secretory (Sec) system or the twin-arginine translocation (Tat) system followed by the T2S-dependent delivery of folded proteins across the outer membrane (OM) [6]. T2S systems have, therefore, been referred to as terminal branch of the general secretory pathway (GSP) [7]. Structural components of T2S systems are often encoded by a single operon containing 12 – 16 genes and assemble in three subcomplexes: (i) an IM-associated assembly platform consisting of GspC, GspF, GspL, GspM and the cytoplasmic ATPase GspE, (ii) a periplasmic pseudopilus containing the major pseudopilin GspG capped by the minor pseudopilins GspH, GspI, GspJ and GspK, and (iii) an OM secretin assembled by GspD [3–5]. Targeting of the secretin to the OM often requires a pilotin, GspS [3,8]. Furthermore, in some T2S systems, assembly of the secretin and its transport to the OM is supported by GspA and GspB, which interact with the peptidoglycan layer [3]. The secretin channel consists of a membrane-spanning β-barrel structure which is built by the C-terminal domains of 15 GspD proteins. The N-terminal regions of GspD proteins form a periplasmic ring structure and consist of four subdomains designated N0, N1, N2 and N3. The N0 domains associate with the periplasmic “homologous regions” (HR) of the IM protein GspC which is part of the assembly platform and presumably forms a cage-like structure upon interaction with the N0 domains [3,4,9–11]. A second periplasmic domain of GspC proteins, hereafter designated “second periplasmic (2P) region”, likely acts as a docking site for T2S substrates in the periplasm. The 2P region is variable in different GspC proteins and often contains a PDZ fold consisting of six β sheets flanked by two α helices. In some species, however, the 2P region only contains a single α helix [8,12,13]. GspC surrounds the central component of the assembly platform, GspF, and forms a complex with the IM proteins GspL and GspM which homo- and heterooligomerize via their periplasmic domains, possibly with variable assembly states in response to substrate binding [8,11,14–18]. A periplasmic protein complex likely corresponding to six copies of each GspL and GspM was visualized by cryo-electron tomography of the T2S system from *Legionella pneumophila in vivo* [18]. Furthermore, cryo-electron microscopy and negative stain electron microscopy of an assembled T2S system from *Klebsiella pneumoniae* revealed a hexameric structure formed by GspL, GspM and the ATPase GspE with each component predicted to interact with two copies of GspC [10,11]. Given the symmetry mismatch between 12 copies of GspC and the pentadecameric GspD secretin, it was speculated that the docking of the assembly platform to the secretin GspD leads to structural reorganizations in GspD. Alternatively, openings in the GspC - GspD complex and local rearrangements might control the access of T2S substrates [8,11,19]. It is still unknown how T2S substrates are recognized because their only apparent common feature is a cleavable Sec or Tat signal whereas a conserved signal for T2S-dependent transport across the OM has not yet been identified [8,16]. Given that T2S substrates are folded in the periplasm prior to T2S-dependent transport, the secretion signal is likely part of a structural element which interacts with components of the assembly platform such as the secretin or the 2P domain of GspC [3,16,20]. Additional potential substrate docking sites are provided by the periplasmic pseudopilus which assembles on top of the IM protein GspF and presumably pushes T2S substrates through the secretin by continuous assembly and disassembly [4,17].

In our laboratory, we study T2S in *Xanthomonas euvesicatoria,* which is a Gram-negative gamma-proteobacterium and the causal agent of bacterial spot disease in pepper and tomato plants. *X. euvesicatoria* multiplies locally in the apoplast and utilizes different protein secretion systems to secrete virulence factors into the extracellular milieu or directly into the plant cell [21–26]. Key pathogenicity factor is the type III secretion (T3S) system, which delivers effector proteins into plant cells and allows the manipulation of plant cellular pathways such as defense responses to promote bacterial infection and multiplication [27,28]. In addition to the T3S system, successful colonization of host plants depends on the Xps-T2S system, which is one of two T2S systems from *X. euvesicatoria* and secretes degradative enzymes. We previously showed that deletion of genes encoding the putative ATPase XpsE or the secretin XpsD leads to reduced virulence and extracellular protease and xylanase activity of *X. euvesicatoria*. In agreement with this finding, *in vitro* secretion of two xylanases and a predicted protease was significantly reduced in the absence of a functional T2S system [29,30]. Residual secretion in T2S mutants is likely caused by the export of proteins via outer membrane vesicles (OMVs), which is an alternative way for periplasmic proteins to reach the extracellular milieu [30,31]. Type II-secreted enzymes are predicted to degrade components of the plant cell wall to facilitate nutrient acquisition and the assembly of the T3S pilus, which spans the cell wall and delivers type III effectors (T3Es) to the plant plasma membrane. In agreement with the anticipated interplay of T2S and T3S systems, expression studies showed that the corresponding genes are coregulated and specifically activated during plant infection [29,30]. Components of the Xps-T2S system from *Xe* are encoded by a chromosomal cluster of eleven genes and include XpsE, F, G, H, I, J, K, L, M, C and D [29]. Letters refer to the nomenclature of corresponding Gsp proteins. A homolog of the prepilin peptidase GspO, which processes the minor pseudopilins prior to pseudopilus assembly, is encoded outside of the *xps*-T2S gene cluster [3,4,25]. A predicted pilotin and homologs of GspA and GspB are missing in *X. euvesicatoria*. XpsC (formerly designated XpsN) was considered as a functional equivalent of GspN which is a fifth component of the assembly platform and is present in some but not all T2S systems [3,4]. However, due to the presence of a predicted HR region and structural similarities with other known GspC proteins, it is hereafter referred to as XpsC [23,32].

In the present study, we analysed the functions of the predicted assembly platform components XpsC, XpsL and XpsM from *X. euvesicatoria*. To facilitate genetic manipulations, we generated a modular T2S gene cluster by Golden Gate-based hierarchical assembly of single promoter and gene modules. The modular design allowed the rapid deletion of single and multiple genes and the insertion of reporter fusions by replacement of individual modules. Using mutant approaches, *in vivo* interaction and localization studies, we show that components of the assembly platform are essential for T2S and form structurally conserved complexes which are independent of the OM secretin.

## Results

### Generation of a modular *xps-*T2S gene cluster by Golden Gate cloning

**Fig 1:**
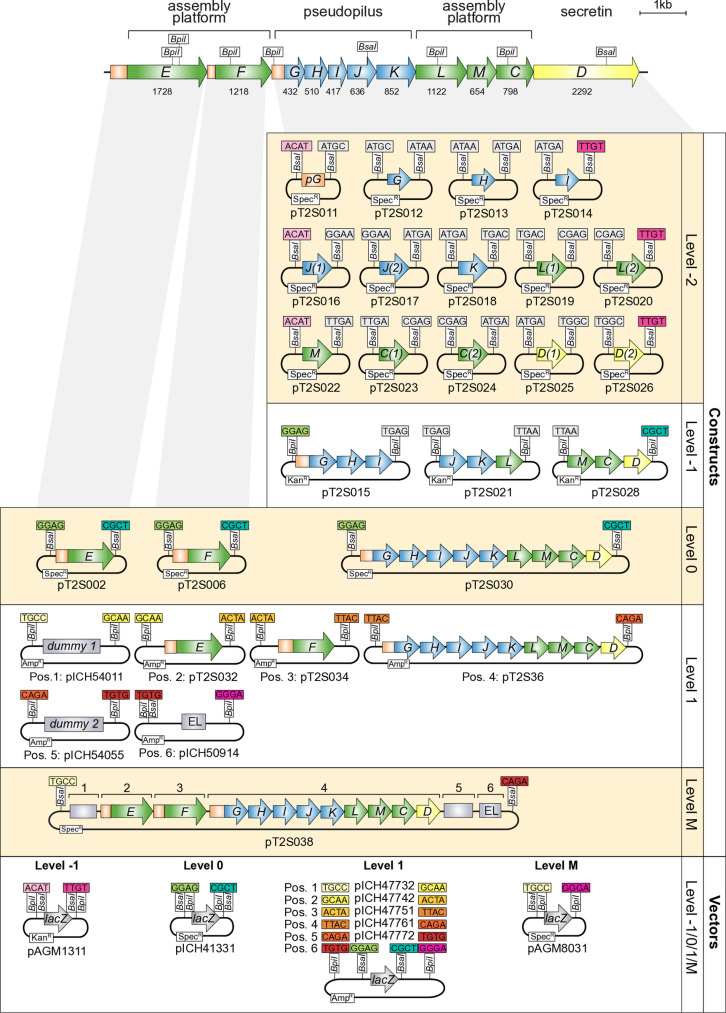
Schematic representation of the *xps-*T2S gene cluster and overview of cloning steps for the generation of the modular *xps-*T2S gene cluster. Genes are represented by arrows, promoters by orange boxes. Letters refer to the nomenclature of *xps* genes. The position of internal *Bsa*I and *Bpi*I restriction sites is indicated. Numbers below the arrows refer to the length of the single genes in base pairs. For the modular assembly, gene and promoter fragments were amplified by PCR and cloned into level −2 vectors (*xpsG* to *xpsD*) or level 0 vectors (*xpsE* and *xpsF*) as indicated. Restriction/ligation using the type IIs restriction enzymes *Bsa*I and *Bpi*I in alternating order led to the assembly of transcription units in level 0 and level 1 vectors and of the entire *xps-*T2S gene cluster in the level M vector. The final level M construct contains an end-linker (EL) and two dummy modules (grey boxes) which can be replaced by additional genes or reporter fusions.

The Xps-T2S system from *X. euvesicatoria* strain 85-10 is encoded by a chromosomal gene cluster which contains eleven genes organized in three predicted operons comprising *xpsE*, *xpsF* and *xpsGHIJKLMCD* (Fig 1). The corresponding gene products constitute the components of the assembly platform, the pseudopilus, the ATPase and the secretin of the T2S system (S1 Fig). In previous studies, we showed that the ATPase XpsE and the secretin XpsD are essential for T2S [29]. For this, the corresponding genes were deleted from the genome of *X. euvesicatoria* strain 85-10 by homologous recombination which is a time-consuming approach including several selection steps and the PCR-based analysis of recombination events. Furthermore, complementation of the respective mutant phenotypes often involves the reintroduction of the deleted gene on a plasmid which leads to higher gene copy numbers and thus possibly interferes with protein function. To facilitate mutagenesis of *xps* genes and complementation studies, we generated a modularized *xps-*T2S gene cluster by hierarchical Golden Gate-based cloning and assembly of gene and promoter fragments [33–35]. The modular design allows genetic manipulations by rapid exchange of single gene modules in a multi-gene construct. For this, individual promoters and genes were subcloned and subsequently assembled by Golden Gate-based modular cloning (MoClo), using the type IIs restriction enzymes *Bsa*I and *Bpi*I. Both enzymes cut DNA outside of their recognition sites. The resulting 4-bp overhangs allow the ordered assembly of DNA fragments by one-pot restriction/ligation reactions [33,34]. MoClo destination vectors designated level −2, −1, 0, 1 and M are available and contain the *lacZα* fragment flanked by *Bsa*I and *Bpi*I sites in inverse orientations and alternating order (Fig 1). This design allows the stepwise cloning of operons and multi-gene constructs in which the internal *Bsa*I and *Bpi*I sites have been removed by PCR-based mutagenesis prior to assembly (Fig 1). For modular cloning, single gene and predicted promoter fragments of the *xpsG* to *xpsD* operon were subcloned into level −2 vectors, preassembled in level −1 vectors and transferred to a level 0 vector (Fig 1). The corresponding construct was subsequently assembled with additional level 0 modules containing *xpsE* and *xpsF* genes to generate the complete *xps-*T2S gene cluster. Two dummy modules were included upstream and downstream of the *xps-*T2S gene cluster, which can be replaced by genes or reporter fusions in the final construct. A series of level 1 vectors with different *Bpi*I fusion sites allows the ordered assembly of modules in a final level M vector (Fig 1). The 3’ *Bpi*I site of the last level 1 module is connected to the level M vector by an end-linker (Fig 1). A similar method was previously used to generate a modular T3S gene cluster from *X. euvesicatoria* [36].

### The modular Xps-T2S system is functional in *X. euvesicatoria*

**Fig 2:**
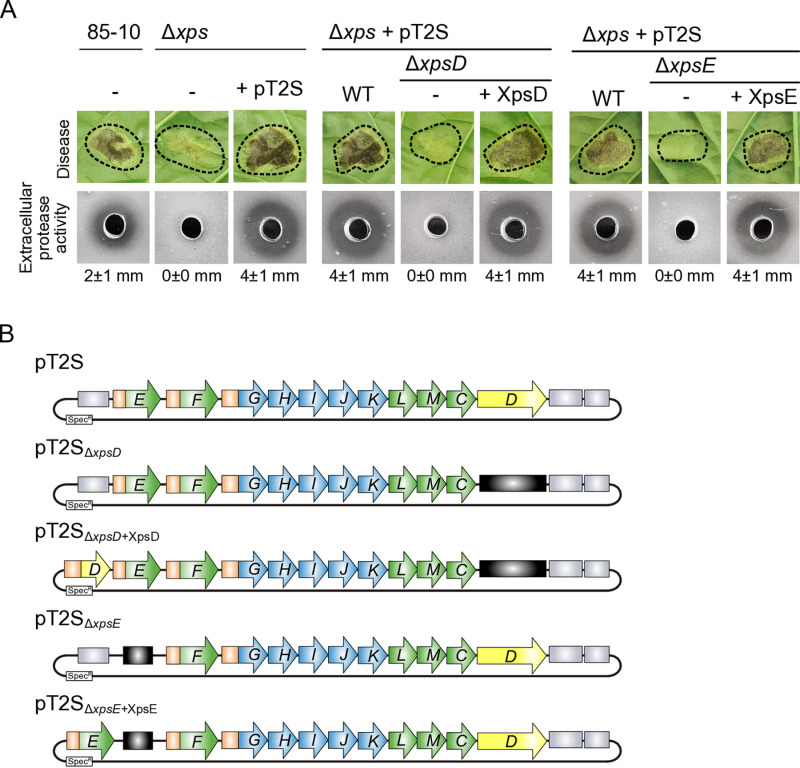
The modular *xps-*T2S system is functional in *X. euvesicatoria* strain 85-10 ∆*xps*. (A) The wild-type strain 85-10 and the T2S deletion mutant 85-10∆*xps* (∆*xps*) with (+) or without (−) the modular *xps-*T2S expression construct (+ pT2S) were analysed for bacterial virulence and extracellular protease activity. For the analysis of *xpsD* and *xpsE* mutants, modular T2S gene cluster constructs lacking *xpsD* (∆*xpsD*) or *xpsE* (∆*xpsE*) were introduced into strain 85-10∆*xps.* For complementation studies, *xpsD* (+XpsD) or *xpsE* (+XpsE) expression cassettes were inserted at position 1 of the respective constructs as depicted in (B). Bacteria were infiltrated at a density of 2 × 10^7^ CFU (colony-forming units) ml^−1^ into leaves of susceptible pepper plants and disease symptoms were photographed 8 dpi (days post inoculation). Dashed lines indicate the infiltrated areas. For the analysis of extracellular protease activity, bacteria were grown on 1% (w/v) milk plates for 2 days and halo formation was documented. Bacteria were removed from the holes prior to documentation. The numbers refer to the width of the halos in millimeters, with mean values calculated from three replicates. (B) Overview on modular level M T2S constructs generated for the analysis of *xpsE* and *xpsD* mutants. Genes are represented by arrows, promoters by orange boxes. Deletions are indicated by black boxes. For complementation studies, *xpsE* and *xpsD* were reinserted at position 1 of the corresponding modular T2S gene cluster constructs as indicated.

For functional studies, the final level M expression construct containing the modularized T2S gene cluster was introduced into strain 85-10∆*xps*, in which the entire chromosomal *xps* gene cluster had been deleted from the chromosome. When bacteria were syringe-infiltrated into leaves of susceptible pepper plants, the wild-type strain 85-10 induced disease symptoms in form of water-soaked lesions whereas reduced symptoms were observed after infiltration of strain 85-10∆*xps* as expected (Fig 2A) [29]. The wild-type phenotype was restored upon introduction of the modular T2S gene cluster on a low copy level M vector (Fig 2A). We also conducted dip infection assays which simulate more natural conditions compared to the syringe infiltration experiments. Dip infection of pepper leaves with the wild-type strain 85-10 led to the formation of bacterial spot symptoms whereas spot formation was significantly reduced after infection with strain 85-10∆*xps* (S2 Fig). As with the phenotypes observed after syringe infiltration, the modular T2S gene cluster restored wild-type levels of spot formation when analysed in the *xps* deletion mutant (S2 Fig). In addition to infection experiments, we monitored extracellular protease activity on milk-containing agar plates*.* As reported previously, strain 85-10 led to the formation of a cleared halo around the inoculation site due to the degradation of milk proteins whereas halo formation was severely reduced for strain 85-10∆*xps* [29,37] (Fig 2A). In the presence of the modular T2S gene cluster, extracellular protease activity was restored and even more prominent than in strain 85-10, suggesting that the plasmid-encoded modular *xps-*T2S gene cluster is functional (Fig 2A).

We next analysed the suitability of the modular T2S gene cluster for mutational approaches and complementation studies in *X. euvesicatoria* strain 85-10∆*xps*. We previously showed that T2S depends on the secretin XpsD and the ATPase XpsE [29,30]. As proof of concept, we individually replaced the corresponding gene modules by short linker sequences and introduced the reassembled modified T2S gene clusters into strain 85-10∆*xps* (Fig 2B)*.* The absence of *xpsE* or *xpsD* led to reduced virulence and extracellular protease activity as expected (Fig 2A) [29]. For complementation studies, *xpsE* and *xpsD* were inserted under control of their native promoters at position 1 upstream of the modular *xps* gene clusters in the final level M constructs (Fig 2B). The corresponding expression constructs fully restored virulence and extracellular protease activity in strain 85-10∆*xps*, suggesting that *xpsE* and *xpsD* were functional when expressed next to the modular *xps* gene cluster under control of their native promoters in the final level M construct (Fig 2A). Taken together, these experiments demonstrate the suitability of the modular T2S gene cluster for mutant and complementation studies in *X. euvesicatoria*.

### Generation of single gene deletions in the modular T2S gene cluster reveals an essential role of predicted assembly platform components

**Fig 3:**
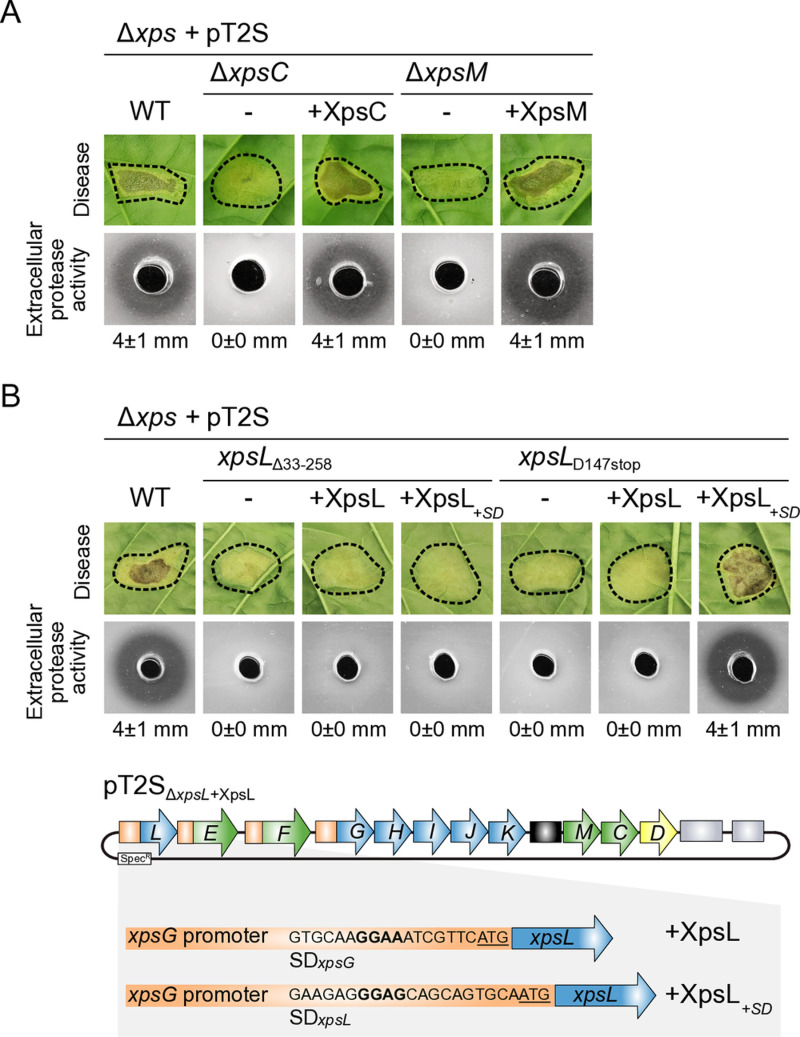
The predicted assembly platform components XpsC, XpsL and XpsM are essential for T2S in *X. euvesicatoria.* (A) Complementation studies with *xpsC* and *xpsM* mutants. Infection and protease activity assays were performed with derivatives of *X. euvesicatoria* strain 85-10∆*xps* with modular expression constructs containing the wild-type (WT) T2S gene cluster (pT2S) or derivatives thereof lacking *xpsC* (∆*xpsC*) or *xpsM* (∆*xpsM*)*.* For complementation studies, expression cassettes encoding XpsC (+XpsC) or XpsM (+ XpsM) under control of the *xpsG* promoter were inserted at position 1 of T2S gene cluster constructs lacking *xpsC* and *xpsM*, respectively. For infection studies, bacteria were infiltrated at a density of 2 × 10^7^ CFU (colony-forming units) ml^−1^ into leaves of susceptible pepper plants and disease symptoms were photographed 8 dpi (days post inoculation). Dashed lines indicate the infiltrated areas. For the analysis of extracellular protease activity, bacteria were grown on 1% (w/v) milk plates for 2 days and halo formation was documented. The numbers refer to the width of the halos in millimeters, with mean values calculated from three replicates. (B) *xpsL* is essential for T2S and depends on its native Shine Dalgarno sequence for efficient expression. The contribution of *xpsL* to virulence and extracellular protease activity was analysed in derivatives of strain 85-10∆*xps* containing the wild-type T2S gene cluster or derivatives thereof lacking *xpsL* (∆*xpsL*) with or without expression cassettes encoding XpsL under control of the native *xpsG* promoter (+XpsL). In a second construct, the predicted Shine Dalgarno (SD) sequence of *xpsG* (indicated in bold letters) was exchanged by the corresponding sequence upstream of *xpsL* (XpsL_+SD_) as illustrated. The translation initiation codon (ATG) is underlined. For complementation studies, bacteria were infiltrated into leaves of susceptible pepper leaves and were grown on milk-containing agar plates. Protease activity was monitored as described in **(A)**.

According to similarities between Xps and Gsp proteins, the T2S system from *X. euvesicatoria* consists of the OM secretin XpsD, a central periplasmic pseudopilus and an assembly platform containing the IM protein XpsF which is associated with the cytoplasmic ATPase XpsE and surrounded by XpsC, XpsL and XpsM [3]. Complexes of XpsC, XpsL and XpsM homologs have been detected in *E. amylovora* and *X. campestris* pv. *campestris*, however, their structure and contribution to the formation of the assembly platform is largely unknown [38,39]. In the present study, we analysed the functions and interactions of XpsC, XpsL and XpsM using the modular T2S gene cluster from *X. euvesicatoria*. For this, we generated level M expression constructs in which the individual genes were replaced by short linker sequences (S3 Fig). For complementation studies, individual assembly platform genes were reinserted under control of the native *xpsG* promoter at position 1 of the corresponding modular gene clusters (S3 Fig). When analysed in strain 85-10∆*xps,* modular T2S gene cluster constructs deleted in *xpsC, xpsL* or *xpsM* led to reduced virulence and extracellular protease activity, indicative of a loss of T2S (Fig 3A and 3B). *In cis* expression of *xpsC* and *xpsM* in the corresponding mutant T2S gene clusters restored the wild-type phenotype, suggesting that reduced virulence and loss of detectable extracellular protease activity were specifically caused by the absence of XpsC or XpsM and not by a polar effect of the deletions on neighbouring genes (Fig 3A). In case of the *xpsL* mutant, however, reinsertion of *xpsL* under control of the native promoter did not restore the wild-type phenotype in the *xpsL* mutant (Fig 3B). To exclude that the lack of complementation was caused by a polar effect of the deletion in *xpsL* on the expression of downstream genes in the *xps-*T2S gene cluster, we generated an additional *xpsL* mutant containing a nonsense mutation at codon position 147 of *xpsL* and repeated the infection and complementation studies. Similar to the *xpsL* deletion, the stop codon in *xpsL* resulted in reduced virulence and extracellular protease activity when the corresponding modular *xps-*T2S gene cluster construct was analysed in strain 85-10∆*xps* (Fig 3B). However, *in cis* expression of *xpsL* under control of the *xpsG* operon promoter in the corresponding modular expression construct did not complement the mutant phenotype (Fig 3B). To investigate whether the lack of complementation was caused by alterations in the expression level of *xpsL*, we replaced the predicted Shine Dalgarno sequence in the *xpsG* promoter by the corresponding sequence which is present upstream of *xpsL* (Fig 3B). The resultling *xpsL* expression cassette restored the wild-type phenotype, suggesting that the function of XpsL is tightly linked to its level of translation which is controlled by the Shine Dalgarno sequence (Fig 3B).

### Structural modeling reveals a trimeric XpsCLM complex which is conserved in different bacterial species

**Fig 4:**
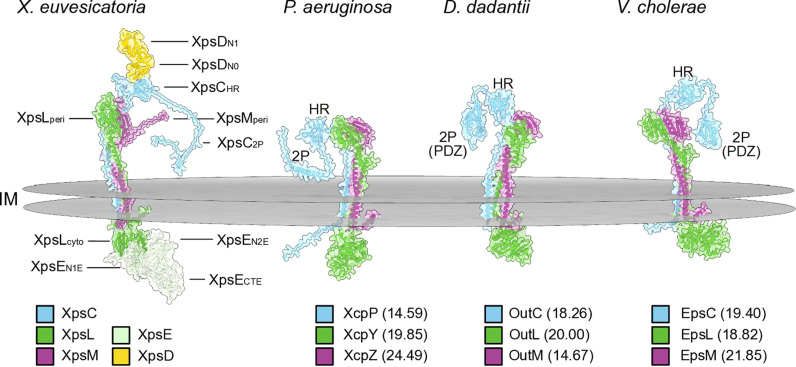
XpsCLM form a predicted trimeric complex which is structurally conserved in different bacterial species. The structure of a complex consisting of the periplasmic region of XpsD (N1 and N0 domains), XpsC, XpsL, XpsM and XpsE was predicted using the AlphaFold2 algorithm and the molecular visualization program UCSF ChimeraX. Proteins are represented in different colours as indicated. The HR and 2P domains of XpsC, the periplasmic (peri) and cytoplasmic (cyto) domains of XpsL and XpsM and the position of the inner membrane (IM) are indicated. The N termini of XpsC, XpsL and XpsM are located in the cytoplasm. XpsE consists of two N-terminal (N1E and N2E) and one C-terminal (CTE) domain. Ribbon diagrams for the XpsCLM complex and for single proteins are shown in S4 Fig. The per-residue model confidence score (pLDDT) of single protein complexes is shown in S6 Fig. AlphaFold2 predictions are also presented for the GspCLM proteins XcpPYZ from *P. aeruginosa* strain PAO1, OutCLM from *D. dadantii* strain 3937 and EpsCLM from *V. cholerae* strain N16961. HR and 2P domains of GspC proteins are indicated. The 2P domains of OutC and EpsC contain a PDZ fold. Numbers refer to the amino acid identities with the corresponding XpsCLM proteins from *X. euvesicatoria.* The following proteins were used for the topology models: XcpP (accession number CAA48581), XcpY (accession number AAG06484) and XcpZ (accession number AAG06483) from *P. aeruginosa* strain PAO1, OutC (accession number CAA46369), OutL (accession number ADM99368) and OutM (accession number ADM99367) from *D. dadantii* strain 3937 and EpsC (accession number P45777), EpsL (accession number P45782) and EpsM (accession number P41851) from *V. cholerae* strain N16961*.*

XpsC, XpsL and XpsM are predicted IM proteins with periplasmic domains and likely surround the central XpsF component of the assembly platform. To predict the architecture of the XpsCLM complex in the context of the T2S system, we modeled the corresponding protein structures *in silico* using the AlphaFold2 structure prediction algorithm and the molecular visualization program UCSF ChimeraX [40,41]. When included in one structural model, XpsC, XpsL and XpsM form a trimeric complex with a pTM (predicted template modelling) value of 0.56 and an ipTM (inter-chain predicted template modelling) value of 0.62 (see Material and Methods, Figs 4 and S6). The pTM score assesses the confidence in the relative arrangement of domains within a single protein chain whereas the ipTM score evaluates the reliability of predicted conformations and interactions between protein chains in a multimer structure. pTM and ipTM values range from 0 to 1, and values closer to 1 indicate higher confidence. According to the AlphaFold2 predictions, XpsC, XpsL and XpsM insert into the IM with an N-terminal transmembrane helix followed by a large C-terminal periplasmic region. The cytoplasmic domain of XpsL is predicted to associate with the putative ATPase XpsE, albeit with lower pTM and ipTM values of 0.48 and 0.51, respectively (Figs 4 and S6). The periplasmic regions of XpsL and XpsM adopt potential ferredoxin-like folds containing two α helices and an anti-parallel β sheet (S4 Fig). According to the structural model, XpsL and XpsM interact with each other via their transmembrane and periplasmic regions including the predicted ferredoxin-like fold as was previously described for GspL and GspM from *Klebsiella oxytoca* and *Dickeya dadantii* [14,39]. Additionally, both XpsL and XpsM likely interact with the periplasmic region of XpsC (S5 Fig).

XpsC was previously designated XpsN but is a homolog of GspC [32]. The periplasmic region of XpsC contains an HR domain which is formed by β sheets and is also present in other GspC proteins including XcpP from *P. aeruginosa*, OutC from *D. dadantii* and EpsC from *V. cholerae* (Figs 4 and S5). The HR domain of XpsC is predicted to interact with the periplasmic domain of the secretin XpsD (Fig 4). A second periplasmic region, referred to as 2P domain, contains a single predicted α helix as described for XcpP from *Pseudomonas aeruginosa* [42]*.* In contrast, a PDZ fold containing α helices and β sheets is present in the 2P domains of the GspC proteins OutC and EpsC from *D. dadantii* and *V. cholerae* [12,43] (Figs 4 and S5). PDZ domains also occur in T2S system-unrelated proteins and are often involved in protein-protein interactions [44,45]. Interestingly, AlphaFold modeling revealed a similar structure of trimeric GspCLM complexes from the bacterial pathogens *P. aeruginosa* (XcpPYZ), *D. dadantii* (OutCLM) and *V. cholerae* (EpsCLM), despite limited amino acid sequence similarities of the corresponding proteins. The pTM values of 0.42 for all three complexes and the ipTM values of 0.41 (for XcpPYZ), 0.42 (for OutCLM) and 0.48 (for EpsCLM) were slightly lower than the corresponding values for XpsCLM from *X. euvesicatoria* (see above). Notably, however, significant portions of the GspC homologs in all models were highly disordered, which might result in lower pTM and ipTM values (see S6 Fig). Taken together, we conclude from these structure predictions that the architecture of XpsCLM complexes is likely conserved across highly divergent T2S sytems (Figs 4 and S6).

### Bacterial two-hybrid studies revealed interactions between XpsC, XpsL, XpsM and XpsE

**Fig 5:**
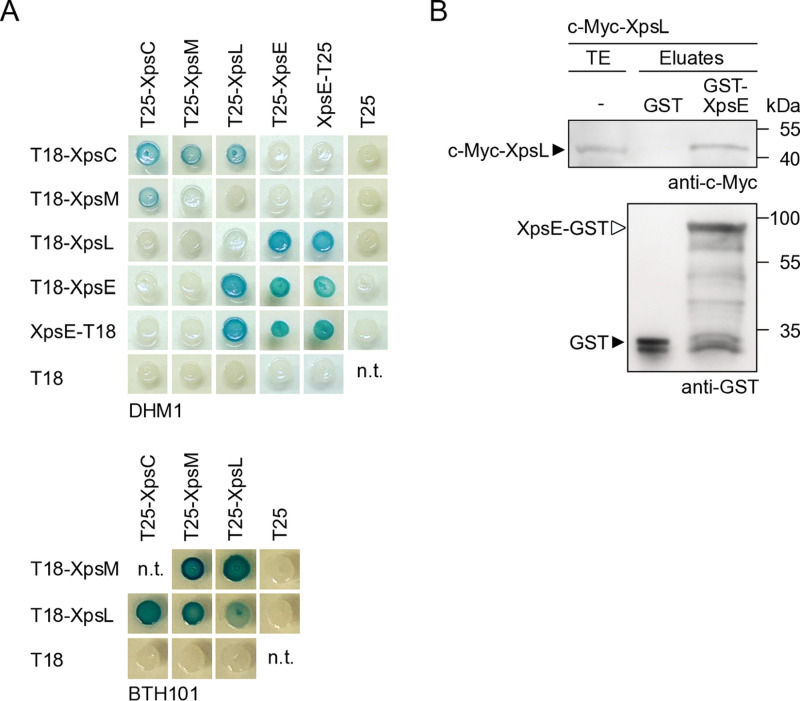
*In vitro* protein-protein interaction studies with predicted assembly platform components from *X. euvesicatoria.* (A) BACTH assays with XpsC, XpsL, XpsM and XpsE. T25 and T18 fusions of XpsC, XpsL, XpsM and XpsE as indicated were analysed in the *E. coli* reporter strains DHM1 and BTH101 as indicated. Transformants were incubated on indicator plates containing X-Gal and IPTG and representative colonies were photographed after five days. All fusions proteins were stably synthesized as was shown by immunoblot analysis (S7 Fig). Experiments were performed at least three times with similar results. One representative colony is shown. n.t., not tested. (B) *In vitro* interaction of XpsL and XpsE. GST and GST-XpsE were immobilized on a glutathione sepharose matrix and incubated with a bacterial lysate containing c-Myc-XpsL. The total cell extract (TE) and eluted proteins (eluate) were analysed by immunoblotting using c-Myc- or GST-specific antibodies. Signals corresponding to GST and GST-XpsE are indicated by asterisks. The experiment was performed three times with similar results. One representative result is shown.

To experimentally validate the predicted interactions between XpsC, XpsL, XpsM and XpsE, we performed protein-protein interaction studies using the bacterial adenylate cyclase two-hybrid (BACTH) system. This method is based on the reconstitution of the catalytic domain of the adenylate cyclase (Cya) from two subdomains (T18 and T25) in the bacterial cytoplasm and is suitable for the analysis of transmembrane proteins [46,47]. The T18 and T25 subdomains of Cya were analysed as N-terminal fusion partners of predicted assembly platform components to allow their localization in the cytoplasm (Fig 4). According to topology predictions, the C-terminal regions of XpsC, XpsL and XpsM are located in the periplasm (Fig 4). A C-terminal T18 or T25 fusion partner would, therefore, prevent the formation of a functional catalytic Cya domain in the cytoplasm. For the putative ATPase XpsE, however, which is located in the cytoplasm, T18 and T25 subdomains were analysed as both N- or C-terminal fusion partners. Immunoblot analysis of bacterial cell extracts showed that all proteins were stably synthesized (S7 Fig). For BACTH assays, T18 and T25 fusions were co-expressed in the *E. coli* reporter strain DHM1, which lacks the native *cya* gene. Protein-protein interactions led to cAMP production and thus to *lacZ* gene expression which was monitored when bacteria were grown on reporter plates containing X-Gal. We detected self-interactions of XpsE and XpsC, interactions of XpsE with XpsL as well as of XpsC with both XpsM and XpsL (Fig 5A). The analysed fusion proteins did not bind to the T18 or T25 subdomains alone, suggesting that the observed interactions were specific (Fig 5A). We did not detect interactions between XpsM and XpsL in strain DHM1 and, therefore, repeated the BACTH assays using the more sensitive reporter strain BTH101, which grows faster than strain DHM1 and leads to enhanced LacZ activity [46]. When analysed in strain BTH101, XpsM and XpsL interacted with themselves and with each other, suggesting that they can form homo- and heterooligomeric complexes (Fig 5A). To confirm the interaction between XpsE and XpsL, we performed *in vitro* GST (glutathione S-transferase) pull-down assays. For this, GST and GST-XpsE were immobilized on glutathione sepharose and incubated with bacterial lysates containing an N-terminally c-Myc epitope-tagged derivative of XpsL. Immunoblot analyses showed that c-Myc-XpsL coeluted with GST-XpsE but not with GST alone, suggesting a direct interaction between XpsE and XpsL (Fig 5B). Our results are in agreement with the previous finding that the ATPase XpsE from *X. campestris* pv. *campestris* oligomerizes and interacts with XpsL [48].

### Stability of XpsC and XpsL depends on XpsM

Next, we analysed predicted components of the assembly platform *in vivo* in the context of the native T2S system. To detect XpsL, we introduced a c-Myc epitope at the N terminus because a C-terminal epitope might interfere with the predicted interaction of XpsL with XpsC and XpsM. The corresponding expression cassette containing *c-myc-xpsL* under control of the *xpsG* promoter was inserted into the flanking region of modular *xps* gene cluster constructs, which were mutated in *xpsL* or additionally lacked *xpsC*, *xpsM*, *xpsE, xpsF* or *xpsD* (S3 Fig). The resulting constructs were transferred into strain 85-10∆*xps* and assessed for T2S system activity and the synthesis of c-Myc-XpsL*.*

**Fig 6:**
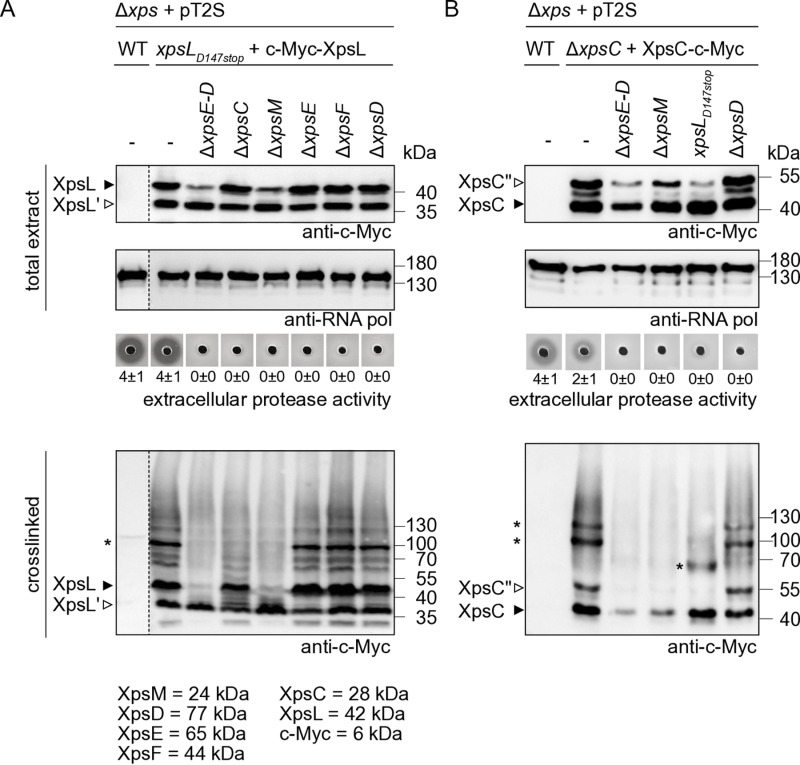
*In vivo* crosslinking experiments reveal the presence of a trimeric XpsCLM complex which assembles independently of the secretin XpsD. (A) Detection of XpsL-containing complexes after *in vivo* crosslinking depends on XpsC and XpsM. Derivatives of strain 85-10∆*xps* (∆*xps*) containing the wild-type (WT) modular T2S gene cluster (+pT2S) or derivatives thereof deleted in *xpsL, xpsE-D, xpsC, xpsM, xpsE, xpsF* or *xpsD* and encoding c-Myc-XpsL as indicated were grown in NYG medium. Equal amounts of cell cultures were centrifuged and cells were either resuspended in Laemmli buffer at 99°C (total extract) or incubated with formaldehyde and resuspended in Laemmli buffer at 37°C (crosslinked). Proteins were analysed by immunoblotting using antibodies specific for the c-Myc epitope or the RNA polymerase β to ensure equal loading. Signals corresponding to the expected sizes of c-Myc-XpsL (48 kDa; black arrow), a putative degradation product of c-Myc-XpsL (XpsL’, 37 kDa; white arrow) and a protein complex (100 kDa; asterisk) are indicated. The molecular weight of predicted components of the assembly platform is indicated below the blots. The dotted line indicates that the blot has been cut to exclude an additional lane. For the analysis of T2S system activity, bacteria were grown on milk protein-containing agar plates to demonstrate extracellular protease activity. Halo formation was documented two days after incubation. Experiments were performed three times with similar results. One representative example is shown. The numbers refer to the width of the halos in millimeters, with mean values calculated from three replicates. (B) The formation of XpsC-containing complexes depends on XpsM. Derivatives of strain 85-10∆*xps* (∆*xps*) containing the wild-type modular T2S gene cluster or derivatives thereof deleted in *xpsC, xpsE-D, xpsM, xpsL* or *xpsD* and encoding XpsC-c-Myc as indicated were grown in NYG medium and analysed as described in (A). The signals corresponding to c-Myc-XpsC (black arrow), a potential N-terminally extended derivative or c-Myc-XpsC (XpsC”, white arrow) and c-Myc-XpsC-specific complexes (asterisks) are indicated.

Protease activity assays showed that c-Myc-XpsL restored extracellular protease activity in the *xpsL* mutant, suggesting that it was functional (Fig 6A). When analysed by immunoblotting, c-Myc-XpsL was detected at the expected molecular size of 48 kDa (Fig 6A). An additional protein at a size of approximately 37 kDa, hereafter referred to XpsL’, presumably represents a degradation or cleavage product of c-Myc-XpsL lacking the C-terminal protein region (Fig 6A). Reduced amounts of c-Myc-XpsL were detected in strains lacking the complete *xps-*T2S gene cluster (∆*xpsE-D*) or *xpsM*, suggesting that XpsM contributes to XpsL stability (Fig 6A). Interestingly, however, in contrast to the full-length c-Myc-XpsL protein, the levels of c-Myc-XpsL’ appeared to be unaffected, suggesting that the N-terminal region of XpsL is stable in the absence of XpsM (Fig 6A).

For the analysis of XpsC, a gene module encoding XpsC-c-Myc under control of the *xpsG* promoter was inserted into level M constructs containing modular *xps-*T2S gene clusters with deletions in single (*xpsC*) or multiple *xps* genes as described above (S3 Fig). XpsC-c-Myc restored protease activity in a strain lacking the native *xpsC* gene in the modular T2S gene cluster, suggesting that the C-terminal c-Myc epitope did not significantly interfere with XpsC function (Fig 6B). When analysed by immunoblotting in *X. euvesicatoria* protein extracts, XpsC-c-Myc was detected at a size of approximately 40 kDa instead of the expected size of 32 kDa (28 kDa for XpsC and 4 kDa for the c-Myc epitope) (Fig 6B). Furthermore, an additional XpsC-c-Myc-specific signal (referred to as XpsC”-c-Myc) was present at a size of 55 kDa (Fig 6B). Inspection of the *xpsG* promoter, which is the native promoter of the predicted *xpsG-D* operon and was used to express *xpsC* (see above, Fig 1), revealed the presence of additional potential start codons upstream of the translation initiation site of *xpsC*. Alternative translation initiation might lead to the synthesis of an N-terminally extended derivative of XpsC-c-Myc and could thus explain the detection of XpsC”-c-Myc. In agreement with this hypothesis, the introduction of a nonsense mutation upstream of the start codon of *xpsC* abolished the detection of XpsC” (S8 Fig). These experiments were performed with an N-terminally c-Myc epitope-tagged derivative of XpsC, which was functional and restored the extracellular protease activity in the *xpsC* mutant (S8 Fig).

### Identification of the XpsCLM complex by *in vivo* crosslinking experiments

To investigate a possible complex formation by XpsC and XpsL in *X. euvesicatoria*, we performed crosslinking experiments with formaldehyde (FA) which penetrates cells and often interacts with the amino group of lysine. FA introduces inter- or intramolecular covalent crosslinks between amino acid residues of proteins which are in close proximity (2.3–2.7 Å) to each other [49,50]. Immunoblot analysis of cell extracts containing c-Myc-XpsL revealed a specific signal at approximately 100 kDa and additional less prominent signals at 60 and 70 kDa after FA treatment, suggesting that c-Myc-XpsL integrates into oligomeric protein complexes (Fig 6A). XpsL-containing complexes were also observed upon deletion of *xpsE*, *xpsF* or *xpsD* but were significantly reduced in the absence of the entire *xps-*T2S gene cluster as well as in *xpsC* or *xpsM* mutants (Fig 6A). Given the contribution of XpsC and XpsM to complex formation, we speculate that the signal at 100 kDa corresponds to the predicted trimeric complex of c-Myc-XpsL (48 kDa), XpsC (28 kDa) and XpsM (24 kDa). This hypothesis is supported by the results of our BACTH assays which showed interactions of XpsC, XpsM and XpsL (see above, Fig 5A).

In addition to c-Myc-XpsL, we analysed complex formation by XpsC-c-Myc after FA crosslinking. Immunoblot analyses revealed the presence of XpsC-c-Myc-specific protein complexes at molecular sizes of approximately 100 and 120 kDa after FA treatment (Fig 6B). The signal at 120 kDa likely resulted from incorporation of the N-terminally extended version of XpsC-c-Myc into the complex because it was absent upon introduction of a nonsense mutation upstream of the *xpsC* start codon (see above, S8 Fig). The 100 kDa complex containing XpsC-c-Myc was also detected in a secretin mutant but not in a strain lacking *xpsE-D* (Fig 6B). Furthermore, deletion of *xpsM* abolished complex formation, whereas the absence of *xpsL* led to a smaller complex at a size of approximately 60 kDa (Fig 6B). Given that the 100 kDa complex corresponds to the size of XpsC-c-Myc (40 kDa), XpsL (37 kDa) and XpsM (24 kDa) and was detected after crosslinking for both c-Myc-XpsL and XpsC-c-Myc, we speculate that XpsL and XpsC associate with XpsM in *X. euvesicatoria* and form the predicted XpsCLM trimer. Future crosslinking and mass spectrometry analysis will provide insights into complex formation *in vivo* and will help to identify the interaction sites of the predicted assembly platform components.

### The XpsCLM complex and the secretin assemble independently of each other

It was previously reported that the HR domain of GspC proteins interacts with the periplasmic N0 domain of the secretin and thus connects the IM assembly platform to the OM secretin channel [11]. Our crosslinking experiments with XpsC-c-Myc and c-Myc-XpsL suggested that XpsD is dispensable for the formation of the XpsCLM complex (see above, Fig 6A and 6B). To investigate whether the assembly platform is required for secretin assembly, we performed fluorescence microscopy-based *in vivo* localization studies with XpsD and the red fluorescent reporter mCherry which is active in the periplasm [51]. Fluorescent reporter fusions allow to study the assembly of protein secretion systems and were previously used to analyse the formation of the cytoplasmic sorting platform of the T3S system from *X. euvesicatoria* [36]. Reporter fusion proteins, which are located in the cytoplasm, produce fluorescent signals throughout the entire bacterial cells, whereas reporter fusions integrated into the protein secretion system allow the detection of fluorescent foci which are indicative of complex formation. mCherry was used as C-terminal fusion partner of XpsD because the N terminal region of XpsD is part of a cleavable Sec signal. According to topology predictions, both the N- and C-termini of XpsD are located in the periplasm. Complex formation of XpsD-mCherry should result in the formation of fluorescent foci at the cell periphery as shown previously for PulD of *K. oxytoca* [52].

**Fig 7:**
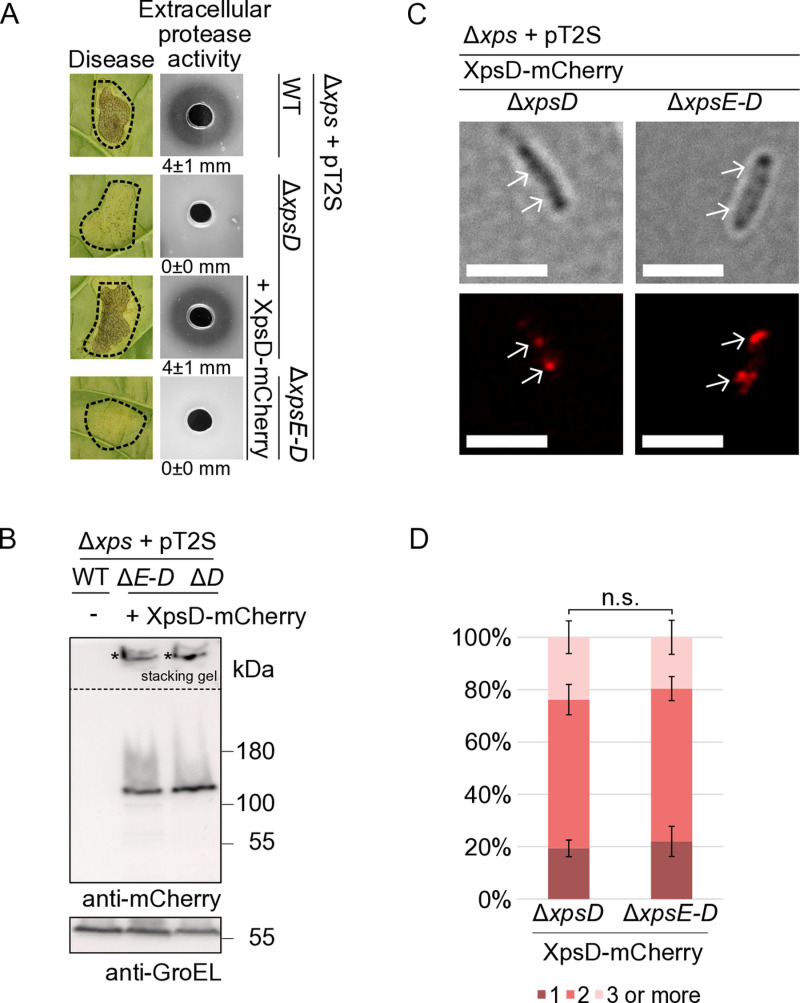
Assembly of the OM secretin XpsD is independent of T2S system components. (A) Complementation studies with XpsD-mCherry in *X. euvesicatoria.* Strain 85-10∆*xps* carrying the wild-type (WT) modular T2S gene cluster (pT2S) or derivatives thereof deleted in *xpsD* (∆*xpsD*) or *xpsE – xpsD* (∆*xpsE-D*) and encoding XpsD-mCherry as indicated were infiltrated at a density of 2 × 10^7^ CFU (colony-forming units) ml^−1^ into leaves of susceptible pepper plants and disease symptom formation was documented 8 dpi (days post inoculation). Dashed lines indicate the infiltrated areas. For the analysis of extracellular protease activity, bacteria were grown on 1% (w/v) milk plates. Halo formation resulting from the degradation of milk proteins was photographed after two days. Experiments were performed three times with similar results. Results from one representative experiment are shown. The numbers refer to the width of the halos in millimeters, with mean values calculated from three replicates. (B) XpsD-mCherry is stably synthesized and forms complexes. Bacterial strains as described in (A) were cultivated in NYG medium and equal amounts of protein extracts were analysed by SDS-PAGE and immunoblotting using mCherry-specific antibodies. The signals labeled with asterisks correspond to XpsD-mCherry-specific complexes which were detected in the stacking gel. The dashed line indicates different exposure times for the lower and upper part of the blot. Blots were reprobed with antibodies directed against GroEL, which was analysed as a loading control. (C) XpsD-mCherry forms fluorescent foci. For fluorescence microscopy studies, strain 85-10∆*xps* (∆*xps*) containing modular T2S gene cluster constructs (pT2S) deleted in *xpsD* (∆*xpsD*) or in the entire cluster (∆*xpsE-D*) and encoding XpsD-mCherry as indicated were incubated in NYG medium for 2 hours. Foci formation was analysed by fluorescence microscopy using a Zeiss LSM 780 AxioObserver Z1 microscope at 60 x magnification. The scale bar corresponds to 3 µm. Bright field images are shown in the upper panel, the signals from the fluorescent channel for mCherry in the lower panel. Fluorescent foci and their positions in the bright field images are indicated with white arrows. The experiment was performed three times with similar results. One representative image for each strain from one experiment is shown. (D) Quantitative analysis of foci formation. Fluorescent foci were counted in at least 100 cells per strain in three different transconjugants. Mean values and standard deviations are shown as percentage of bacterial cells. Asterisks indicate a significant difference between the number of foci with a *p*-value < 0.05 based on the results of a chi-squared test.

Prerequisite for the formation of fluorescent foci is the stability and functionality of the XpsD-mCherry fusion protein. For functional studies, the native *xpsD* gene was deleted from the modular T2S gene cluster and an expression cassette encoding XpsD-mCherry under control of the native promoter was inserted at position 1 of the corresponding level M construct. When bacteria were analysed by infection studies, deletion of *xpsD* led to reduced bacterial virulence in susceptible pepper plants as expected [29] (Fig 7A). XpsD-mCherry restored the wild-type phenotype when encoded in the flanking region of the modular T2S gene cluster lacking *xpsD*, suggesting that the mCherry fusion partner did not interfere with XpsD function (Fig 7A). Immunoblot analyses of bacterial cell extracts showed that XpsD-mCherry was stably synthesized and forms high molecular weight complexes which were detected in the stacking gel and presumably correspond to XpsD-mCherry multimers (Fig 7B). Complex formation was also detected in the absence of *xpsE – xpsD* genes, suggesting that the secretin assembles independently of other components of the T2S system (Fig 7B). Similar findings were observed for a C-terminally c-Myc epitope-tagged derivative of XpsD which was expressed *in trans* under control of a native *Xe-*specific promoter in *xpsD* and *xpsE-D* deletion mutants (S9 Fig). XpsD-c-Myc complemented the *xpsD* mutant phenotype *in planta,* restored extracellular protease activity and formed high molecular weight complexes, even in the absence of other T2S system components, as was shown by immunoblot analysis of bacterial cell extracts (S9 Fig).

To confirm XpsD complex formation in *X. euvesicatoria* T2S mutants, we performed fluorescence microscopy studies with bacteria containing XpsD-mCherry in the presence or absence of other Xps proteins. One to three fluorescent foci per cell were detected in both strains, indicative of the formation of XpsD-mCherry oligomers and thus the assembly of the secretin (Fig 7C and 7D). Foci formation was specific for the presence of XpsD and was not detected in bacteria containing mCherry fused to the effector protein XopB (S10 Fig). No significant differences in foci formation were detectable in the presence or absence of the modular *xps-*T2S gene cluster (Fig 7C and 7D). This confirms the above hypothesis that the secretin assembles independently of other components of the T2S system.

## Discussion

In the present study, we aimed at the functional characterization of the predicted assembly platform components of the Xps-T2S system from *X. euvesicatoria*. We previously reported that the Xps-T2S system is an important virulence factor which depends on the ATPase XpsE and the secretin XpsD for function [29]. Here, we show that the predicted assembly platform components XpsC, XpsL and XpsM form a trimeric complex which is structurally conserved in different bacterial species and represents an essential periplasmic substructure of the T2S system. To expedite functional studies of Xps-T2S system components in *X. euvesicatoria*, we generated a modular T2S gene cluster by stepwise and hierarchical assembly of individual promoter and gene modules using the Golden Gate-based modular cloning strategy [53]. Complementation studies confirmed that the modular T2S gene cluster is functional in *X. euvesicatoria* and restores both virulence and extracellular protease activity in a T2S mutant strain. The modular design allows the rapid generation of single or multiple gene deletions and facilitates the reintegration of genes or reporter fusions at different positions in the gene cluster. Gene expression from the same plasmid does not alter the relative gene copy number and thus avoids negative effects on T2S by increased levels of single components. The generation of deletion mutants and complementation studies using the modular T2S gene cluster confirmed that T2S depends on the ATPase XpsE and the secretin XpsD and demonstrated an equally essential role of the predicted assembly platform components XpsC, XpsL and XpsM.

In addition to functional studies, we used the modularized T2S gene cluster for crosslinking experiments with XpsC, XpsL and XpsM in *X. euvesicatoria* under native conditions. When *X. euvesicatoria* strains with modularized T2S gene clusters expressing c-Myc-fusions of XpsL and XpsC, respectively, were treated with formaldehyde, oligomeric complexes corresponding to the size of the predicted XpsCLM assembly were detected. This is in agreement with previous reports of XpsL - XpsM complexes in *X. campestris* pv. *campestris* and *Erwinia chrysanthemi* [38,39]. Protein studies revealed that XpsM stabilizes XpsL as was also shown for homologous proteins from *P. aeruginosa* [54]. The amounts of the XpsL degradation product, however, remained unchanged in the absence of XpsM, indicating that XpsM predominantly stabilizes the full-length XpsL protein. Given our finding that the function of *xpsL* depends on its native Shine-Dalgarno sequence, we assume that the levels of XpsL are tightly controlled both on the translational level and post-translationally by XpsM.

According to structure predictions using the AlphaFold2 algorithm, XpsC, XpsL and XpsM each insert into the IM via a single N-terminal TM helix followed by a C-terminal periplasmic domain. AlphaFold2 predictions suggest that the trimeric XpsCLM complex shares striking structural similarities with GspCLM complexes from *P. aeruginosa, D. dadantii* and *V. cholerae*, despite overall low amino acid identities of the corresponding proteins. We, therefore, propose that GspCLM complexes represent a conserved structural feature of T2S systems from different bacterial species. According to structure predictions, the periplasmic domains of GspL and GspM proteins include ferredoxin-like folds which likely interact with each other as was shown for GspL and GspM proteins from *Klebsiella* spp. and *D. dadantii* [11,14,15]. GspC proteins contain a periplasmic HR domain which interacts with the N0 domain of the secretin as was reported for GspC and GspD proteins from *E. coli* and *D. dadantii* [10,55–58]. A second periplasmic domain, referred to as 2P domain, forms a flexible and variable structure which folds as a single helix or PDZ structure in different GspC proteins [4,8]. It remains to be investigated whether the 2P domain acts as a binding site for T2S substrates after their Sec- or Tat-dependent transport into the periplasm.

**Fig 8:**
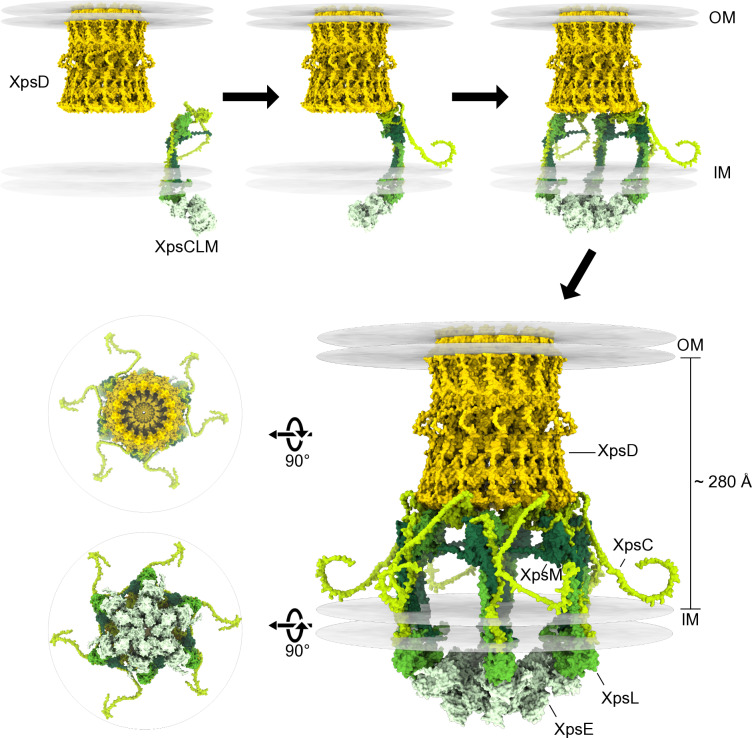
Model of the assembly platform in association with the pentadecameric secretin and the hexameric ATPase. (A) The structure of the XpsCLM complex from *X. euvesicatoria* in context with the OM secretin and the cytoplasmic ATPase was predicted using the AlphaFold2 algorithm and the molecular visualization program UCSF ChimeraX. Proteins are represented in different colours as indicated. To predict the structure of the XpsCLM complex from *X. euvesicatoria* in context with the OM secretin and the cytoplasmic ATPase, the predicted structures of XpsD and XpsE monomers were aligned to crystal structures of the 15-meric *Pseudomonas aeruginosa* PAO1 XcpQ secretin channel (PDB: 5WLN) and the hexameric *V. cholerae* ATPase GspE (PDB: 4KSR). This led to the model of a pentadecameric XpsD and a hexameric XpsE complex which was subsequently aligned with XpsCLM complexes according to the predicted XpsC-XpsD and XpsL-XpsE interactions (see Fig 4). To include the interaction between the N0 domain of XpsD and the HR domain of XpsC in the model of the XpsCLM complex, we slightly repositioned the flexible linker of XpsC which is predicted with low confidence and is located between the second alpha helix and the HR domain (Figs 4, S4 and S6). Hydrophobic residues in structures were used to predict the position of the OM and IM in the structure. The resulting integrated *in silico* model of the core components of the Xps-T2S system from *X. euvesicatoria* suggests the presence of a hexamer of XpsCLM assembled in a cage-like structure and connected to a pentadecamer of XpsD. The positions of the IM and OM are indicated. Top and bottom views of the complex are shown on the left side. According to our model, the pentadecameric XpsD secretin channel and the XpsCLM complex assemble independently of each other. Six XpsCLM complexes each bound to one molecule of the ATPase XpsE associate with the secretin, thus leading to the formation of a predicted cage-like structure connected to a cytoplasmic hexamer of XpsE.

GspC is located at the outside of the GspLM complex and forms a cage-like structure as was shown in *Klebsiella pneumoniae* by cryo-electron microscopy of an assembled T2S system [11]. In our integrated model for *X. euvesicatoria*, six XpsCLM complexes are arranged within the T2S system like columns, connected to the secretin via the HR domains of XpsC. We propose that the stoichiometrical mismatch between these six XpsCLM complexes and the predicted XpsD pentadecamer is resolved by the inherent flexibility of a disordered region in XpsC, located between the HR domain and the binding site for XpsL and XpsM. According to our model, the HR domains of six XpsC molecules interact with six out of 15 N0 domains of XpsD, thus providing positional flexibility and leaving space for T2S substrates to enter the inner vestibule of the secretin channel (Fig 8). In *K. pneumoniae*, however, twelve PulC molecules were predicted to integrate into the assembly platform based on the results of stoichiometry measurements [11]. Since no lateral contacts were detected between the HR domains of PulC proteins, this arrangement could overcome the symmetry mismatch between PulC and PulD by leaving three N0 domains of PulD being unattached [11]. The integration of GspC dimers into the assembly platform of the T2S system has, however, not yet been experimentally confirmed. It is possible that the increased levels of GspC in purified protein fractions are caused by its attachment to the secretin. Alternatively, six additional monomers of GspC could bind to the nine free N0 domains of the secretin channel, alongside the six copies of the GspCLM complex. This could explain why twelve copies of GspC were identified in *K. pneumoniae* [11].

In *X. euvesicatoria*, structural modeling suggests that the XpsCLM complex is connected to the cytoplasmic ATPase XpsE via the cytoplasmic domain of XpsL. The interaction between XpsL and XpsE was verified by BACTH assays and GST pull-down experiments. An interaction with the ATPase was previously also shown for GspL proteins from *K. oxytoca* and *Vibrio* species [11,14,59,60]. Furthermore, cryo-electron microscopy studies of T2S systems from *K. pneumoniae* revealed that the association of the cytoplasmic domain of GspL with GspE leads to the formation of a GspL-GspM-GspE complex which assembles as a flexible hexameric structure [11]. It remains to be investigated whether the ATPase preassembles as a hexamer prior to its incorporation into the T2S system or first binds to GspL as a monomer and only oligomerizes upon hexamerization of the GspCLM complex. To date, a hexameric GspE structure could not be crystallized without an assistant hexameric protein [61], suggesting that the formation of the ATPase complex depends on interactions with additional proteins. The hexameric ATPase is presumably required for the assembly of XpsF and the pseudopilus which are located in the center of the assembly platform. In *K. oxytoca*, the GspF protein PulF was recently demonstrated to form a trimeric ion-channel which fits into the center of the hexameric ATPase PulE [62]. GspF is essential for the formation of the pseudopilus and was shown to interact with both GspL and GspE [11,59,60,63,64].

In *X. euvesicatoria*, our crosslinking experiments with *xps* deletion mutants showed that XpsF, the ATPase XpsE and the secretin XpsD are dispensable for the formation of the XpsCLM complex. Furthermore, fluorescence microscopy studies with an XpsD-mCherry reporter fusion revealed that the formation of secretin complexes occurred independently of the XpsCLM complex. These observations imply that the assembly of the T2S system involves the association of independently generated subcomplexes including the secretin and the XpsCLM complex (Fig 8). It remains to be investigated whether the formation of the OM secretin initiates T2S system assembly as was previously proposed as outside-in model for T2S system assembly in *V. cholerae* and *E. coli* [8,52,65]. The dynamic association of XpsCLM complexes with the secretin might not only lead to T2S system assembly but might also contribute to the regulation of T2S. It is assumed that T2S substrates bind to the 2P region of XpsC or to the entire XpsCLM complex and subsequently associate with the tip proteins of the pseudopilus and the secretin. Unbound XpsCLM modules might dynamically disassemble from the T2S system after secretion of their cargo proteins and might be replaced by substrate-bound XpsCLM complexes. This predicted dynamic assembly of the T2S system could explain why cryo-electron microscopy approaches have so far fallen short in revealing insights into the architecture of T2S systems beyond the structure of the highly stable OM secretin [8,11].

Taken together, we propose an architectural model of the core components of the Xps-T2S system from *X. euvesicatoria* which involves the association of preassembled trimeric XpsCLM complexes with a pentadecameric OM secretin channel. In future studies, we will perform *in vivo* photocrosslinking and interaction studies to analyse the architecture of T2S system components including the IM component XpsF and the pseudopilus proteins XpsG, H, I, J and K. Furthermore, the identification of potential substrate binding sites in T2S system components and secretion signals in T2S substrates might help to uncover how this important transport route for virulence factors is controlled.

## Materials and methods

### Bacterial strains and growth conditions

Bacterial strains and plasmids used in this study are listed in S1 Table. *Escherichia coli* strains were cultivated at 37°C in lysogeny broth (LB) or terrific broth (TB) medium and *X. euvesicatoria* strains at 30°C in nutrient-yeast extract-glycerol (NYG) medium. Plasmids were introduced into chemically competent *E. coli* strains by heat shock and into *X. euvesicatoria* strains by electroporation. Antibiotics were added to the media at the following final concentrations: ampicillin, 100 μg ml^−1^; gentamycin 15 μg ml^−1^; kanamycin, 25 μg ml^−1^; rifampicin, 100 μg ml^−1^ and spectinomycin, 100 μg ml^−1^.

### Deletion of the chromosomal *xps-*T2S gene cluster in *X. euvesicatoria* strain 85-10

To generate strain 85-10Δ*xps*, in which the complete T2S gene cluster is deleted, DNA fragments flanking the *xps* gene cluster were amplified from *X. euvesicatoria* by PCR using the primer pairs Δxps-5’-fw/ Δxps-5’-rv and Δxps-3’-fw/ Δxps-3’-rv (S2 Table). PCR amplicons were cloned into the suicide vector pOGG2 by Golden Gate cloning using *Bsa*I and ligase [33,66]. The resulting deletion construct pOGG2Δxps was introduced into *X. euvesicatoria* strain 85-10 by triparental conjugation using the helper plasmid pRK2013 and transconjugants were selected as described previously [67]. Double crossovers resulted in strain 85-10Δ*xps* which contains a 11,750-bp deletion in the *xps-*T2S gene cluster.

### Generation of expression constructs

To generate expression constructs for BACTH assays, *xps* genes were amplified by PCR from *X. euvesicatoria* using gene-specific primers (S2 Table). PCR amplicons were cloned into vector pAGM9121 using *Bpi*I and T4 ligase or into vectors pICH41021 and pUC57 by blunt-end cloning using *Sma*I and *Eco*RV, respectively. Genes were subsequently transferred to the Golden Gate-compatible BACTH vectors pKT25_GG_, pKNT25_GG_, pUT18_GG_ or pUT18C_GG_ using *Bsa*I and ligase as described previously [33] (S1 Table). For GST pull-down assays, *xpsE* and *xpsL* were amplified by PCR and subcloned into vectors pICH41021 (*xpsL*) and pUC57 (*xpsE*) using *Sma*I or *Eco*RV and ligase. *xpsL* was subsequently cloned into vector pBRNM downstream of an N-terminal c-Myc epitope-encoding sequence using *Bsa*I and ligase. *xpsE* was assembled by Golden Gate cloning with an expression cassette encoding GST under control of the *ptac* promoter in the final destination vector pBRM-Pstop. All constructs are listed in S1 Table.

To generate the expression construct encoding XpsD-c-Myc, the 5’ and 3’ region of *xpsD* was amplified by PCR from *X. euvesicatoria* strain 85-10 and the corresponding amplicons were individually cloned into vector pICH41021 using *Sma*I and ligase in a cut/ligation reaction. The resulting modules were assembled with the promoter of gene XCV4361 in vector pBRM-P by Golden Gate cloning, thus generating construct pB-PxpsD (S1 Table).

### Generation of the modular *xps-*T2S gene cluster by Golden Gate cloning

The *xps-*T2S gene cluster was assembled from single promoter and gene fragments by Golden Gate-based modular cloning [35,53]. For this, the native promoters and coding sequences of *xpsE* and *xpsF* were amplified by PCR using gene-specific primers listed in S2 Table. The resulting PCR amplicons were cloned into the level −1 vector pAGM1311 using *BsaI* and T4 ligase, resulting in constructs pT2S001 (*xpsE* and promoter) and pT2S005 (*xpsF* and promoter). Similarly, the native promoter of the *xpsG-xpsD* operon as well as *xpsG, xpsH, xpsI, xpsJ, xpsK, xpsL, xpsM, xpsC* and *xpsD* were amplified by PCR and cloned into the level −2 vector pAGM9121 using *Bpi*I and T4 ligase, resulting in constructs pT2S011 to pT2S026 (Fig 1). Constructs pT2S011 – pT2S014, pT2S016 – pT2S020 and pT2S022 – pT2S026 were assembled by Golden Gate cloning in the level −1 vector pAGM1311 using *Bsa*I and T4 ligase, thus generating constructs pT2S015 (*xpsG, xpsH* and *xpsI* including the native promoter), pT2S021 (*xpsJ, xpsK and xpsL*) and pT2S028 (*xpsM, xpsC* and *xpsD*). For the generation of level 0 constructs, gene modules from constructs pT2S001 (*xpsE* and promoter), pT2S005 (*xpsF* and promoter) as well as pT2S015, pT2S021 and pT2S028 were inserted into vector pICH41331 using *Bpi*I and ligase, thus leading to the level 0 constructs pT2S002 (*xpsE* and promoter), pT2S006 (*xpsF* and promoter) and pT2S030 (*xpsG - xpsD* operon and promoter). Inserts of level 0 constructs were ligated into different level 1 vectors which determine the positions of the individual modules in the final level M construct [53]. This resulted in constructs pT2S32 (*xpsE* and promoter in vector pICH47742), pT2S34 (*xpsF* and promoter in vector pICH47751) and pT2S36 (*xpsG-xpsD* operon and promoter in vector pICH47761) (Fig 1). Additional level 1 constructs pICH54011, pICH54055 and pICH50914 contained dummy modules for insertion at positions 1 and 5, and an end-linker (Fig 1). The final level M construct pT2S038 was constructed by assembly of level 1 modules in vector pAGM8031 using *Bpi*I and T4 ligase. The end-linker connects the 3’ end of the last module to the destination vector. Constructs used for modular cloning are listed in S1 Table. All constructs were verified by test restriction with appropriate enzymes and all plasmids containing PCR amplicons were sequenced by Sanger sequencing. Deletions, expression cassettes and epitope- or mCherry-encoding sequences were introduced into the modular *xps-*T2S gene cluster as described in S1 Appendix.

### Plant material and infection studies

*Capsicum annuum* cultivar Early Cal Wonder (ECW) plants were grown at 25°C for 16 h of light and 8 h of darkness at 60 – 70% relative humidity. For syringe infections, *X. euvesicatoria* strains were infiltrated with a needleless syringe into the lower side of pepper leaves at concentrations of 2 × 10^7^ CFU (colony-forming units) ml^−1^ in 1 mM MgCl_2_. Symptoms were photographed 6 – 8 days post inoculation (dpi). For dip infections, leaves were incubated in 1 mM MgCl_2_ solutions containing *X. euvesicatoria* at concentrations of 2 × 10^7^ CFU ml^−1^ and 0.02% (v/v) Silwet for at least 30 seconds. The formation of bacterial spot symptoms was monitored 4 – 7 weeks after dipping. Spots were counted in seven infected leaf areas per strain. Statistical significance was determined using an ANOVA with post-hoc HSD (*P* < 0.001). All experiments were performed at least three times with similar results. Representative results from one experiment are shown.

### Protease activity assays

For the analysis of extracellular protease activity, bacteria were grown over night in NYG medium with respective antibiotics. Fourty microlitres of the over-night cultures adjusted to a density of 10^9^ CFU ml^−1^ were pipetted into holes, which were punched out from NYG agar plates containing 1% (w/v) skimmed milk as described previously [29]. Plates were incubated at 30°C for 2 days and bacteria were removed prior to documentation of halo formation. The width of the halos was measured in millimeters and the mean values were calculated from three replicates. Experiments were performed three times with similar results. One representative result is shown.

### Immunoblot analysis of bacterial protein extracts

For immunoblot analysis of protein extracts, bacterial cell pellets were resuspended in Laemmli buffer, denatured by boiling and analysed by SDS-PAGE and immunoblotting, using antibodies specific for the c-Myc epitope (polyclonal antibody from rabbit, Sigma-Aldrich), the FLAG epitope (monoclonal antibody from mouse, Sigma-Aldrich), mCherry (polyclonal antibody from rabbit, Abcam Limited), GroEL (polyclonal antibody from rabbit, Enzo Life Sciences), RNA polymerase β (monoclonal antibody from mouse, Invitrogen), and GST (glutathione *S-*transferase; polyclonal antibody from goat, Cytiva), respectively. Horseradish peroxidase-labelled anti-mouse, anti-rabbit and anti-goat antibodies were used as secondary antibodies. Binding of antibodies was visualized by enhanced chemiluminescence using a Vilber FUSION-FX6 chemiluminescence imager. Results were reproduced at least twice. One representative blot is shown.

### Analysis of protein-protein interactions using the BACTH system

For protein-protein interaction studies, we used a modified version of the Euromedex BACTH system which allowed Golden Gate cloning of genes of interest in fusion with T18- and T25-encoding fragments [68] (see above). For the analysis of protein synthesis, BACTH constructs were introduced into *E. coli* strain JM109 and gene expression was induced by IPTG (isopropyl-β-D-thiogalactopyranoside) for 2 h at 37°C after the optical densities of the bacterial cultures at 600 nm (OD_600_) had reached values between 0.6 and 0.8. Cells were resuspended in Laemmli buffer and analysed by immunoblotting using a FLAG epitope-specific antibody. For interaction studies, the expression constructs encoding T18 and T25 fusion proteins were introduced into the *E. coli* reporter strains DHM1 or BTH101 as indicated and transformants were cultivated on LB agar plates containing kanamycin and gentamycin [69]. At least three transformants for each interaction were grown over night in liquid LB medium with appropriate antibiotics and 2 µl of each over-night culture were spotted on LB agar plates containing kanamycin, gentamycin, X-Gal (5-bromo-4-chloro-3-indolyl-β-d-galactopyranosid; 40 μg/ml) and 2 mM IPTG (isopropyl-*β*-D-thiogalactopyranoside). Plates were incubated for up to seven days at room temperature and the colour of bacterial colonies was documented by photographs. Experiments were performed at least three times with bacteria from independent transformations. One representative colony is shown.

### GST pull-down assays

For GST pull-down assays, *E. coli* TOP10 cells with expression constructs encoding GST, GST-XpsE and c-Myc-XpsL were grown in LB medium at 37°C. When the cultures reached an OD_600_ of 0.6 – 0.8, gene expression was induced by addition of IPTG at a final concentration of 2 mM for two hours. Cells were harvested by centrifugation, resuspended in 1 x PBS buffer and lysed with a French press. Insoluble cell debris were removed by centrifugation and soluble GST and GST-XpsE fusion proteins were immobilized on a glutathione sepharose matrix according to the manufacturer’s instructions (GE Healthcare). The glutathione sepharose with immobilized GST and GST fusion proteins was washed with 1 x PBS buffer and incubated with bacterial lysates containing c-Myc-XpsL for 1 h at 4°C on an overhead shaker. Unbound proteins were removed by washing in 1 x PBS. Bound proteins were eluted with Laemmli buffer and analysed by SDS-PAGE and immunoblotting, using GST- and c-Myc epitope-specific antibodies. Experiments were repeated at least two times with similar results.

### *In vivo* crosslinking experiments

*In vivo* protein complex formation was analysed after crosslinking with FA as described [70]. Briefly, *X. euvesicatoria* strains were grown over night in NYG medium, resuspended at an OD_600_ of 0.3 in fresh NYG medium and incubated on a tube rotator at 30°C. After 3 – 4 hours, cells from 1 ml cultures were collected by centrifugation, resuspended in Laemmli buffer and boiled for 10 minutes (total cell extract). In parallel, cells from 1 ml of cultures were resuspended in 1 ml of 1 mM sodium phosphate buffer (pH 6.8) containing 1% (v/v) FA. After incubation for 20 minutes at room temperature, cells were collected by centrifugation, washed in 1 ml of 1 mM sodium phosphate buffer (pH 6.8) without FA and resuspended in Laemmli buffer. Protein samples were incubated at 37°C for 20 minutes to maintain FA cross-links. Total cell extracts and crosslinked samples were subsequently analysed by SDS-PAGE and immunoblotting, using antibodies specific for the c-Myc epitope and the RNA polymerase β, which was analysed as loading control. Experiments were performed three times with similar results. One representative blot for each experiment is shown.

### Fluorescence microscopy studies

To analyse the localization of an XpsD-mCherry fusion protein, bacteria were grown over night in NYG medium, resuspended in fresh NYG medium at an OD_600_ of 0.15 and incubated on a tube rotator at 30°C for 2 hours. Bacterial cultures were placed on microscopy slides on top of a pad of 1% (w/v) agarose dissolved in NYG medium as described previously [36]. mCherry fluorescence was visualized with a confocal laser scanning microscope (Zeiss LSM 780 AxioObserver. Z1) using filter sets for mCherry (excitation at 587 nm; emission at 610 nm). Experiments were performed with three different transconjugants per construct and repeated two times with comparable results. Fluorescent foci of at least 100 cells per transconjugant were counted. Results from one representative experiment are shown.

### *In-silico* modeling of protein structures using the AlphaFold2 algorithm

The AlphaFold2 algorithm was used for protein structure prediction using the Colabfold server and the molecular visualization program UCSF ChimeraX [40,41,71]. Single structure predictions were performed for XpsE and XpsD, and full-length predictions for complexes of XpsL-XpsE, XpsC-XpsL-XpsM and XpsC_36-169_-XpsD_22-252_. For the generation of an *in silico* model of T2S system core components, the structural model of XpsD was superimposed on a pentadecameric crystal structure of the homologous *Pseudomonas aeruginosa* PAO1 XcpQ secretin channel (PDB: 5WLN) to generate a secretin channel. Furthermore, the XpsE structure was superimposed on the hexameric crystal structure of the homologous ATPase GspE from *V. cholerae* (PDB: 4KSR). The XpsE-XpsL structure prediction was used to align six predicted XpsCLM complexes to the hexameric XpsE structure and the XpsC_36-169_-XpsD_22-252_ prediction was used to align the HR domains of the resulting hexameric XpsCLME complex to the N0 domains of the pentadecameric XpsD complex.

## Supporting information

S1 Table Strains and plasmids used in this study. (PDF)

S2 Table Primers used in this study. (PDF)

S1 Appendix Golden Gate assembly of modular T2S gene cluster constructs. (PDF)

S2 Appendix Supplementary data sets. (PDF)

S1 Fig Schematic representation of the T2S system. The T2S system is composed of the assembly platform (shown in green; GspC, M, L and F and the hexameric ATPase GspE), the pseudopilus (blue; GspG, H, I J and K) and the OM secretin complex (yellow; GspD). Pseudopilins are processed by GspO (light grey), which is encoded outside the *xps-*T2S gene cluster of *X. euvesicatoria*. T2S substrates are exported into the periplasm via the general secretory (Sec) system or the twin-arginine translocation (Tat) system (dark grey) and subsequently recruited by the T2S system. The HR domain of GspC interacts with the secretin GspD. A second periplasmic domain of GspC is indicated as 2P domain. Letters refer to the nomenclature of Gsp proteins. IM, inner membrane; OM, outer membrane.
(PDF)

S2 Fig The modular T2S system restores bacterial spot formation in an *xps*-T2S gene cluster mutant. (A) Leaves of susceptible ECW pepper plant were dip-infected with the wild-type strain 85-10 and the T2S deletion mutant 85-10∆*xps* (∆*xps*) with (+) or without (−) the modular *xps*-T2S expression construct (pT2S). Disease symptom formation was photographed seven weeks after infection. The experiment was performed three times with similar results. Spot formation on one representative leaf per strain is shown. (B) Quantitative analysis of spot formation. Spots were counted in seven different leaf areas per strain. Mean values of the number of spots per cm^2^ of leaf area and standard deviations are shown. Statistical significance was determined using an ANOVA with post-hoc HSD (Honestly Significant Difference) test (*P*<0.001).
(PDF)

S3 Fig Overview on modular T2S gene cluster constructs used in this study. Genes are represented by arrows, promoters by orange boxes. Deletions are indicated by black boxes. Letters refer to the nomenclature of *xps* genes. For complementation and localization studies, genes and gene fusions under control of the *xpsG* promoter were inserted upstream of *xpsE* into the modular T2S gene cluster constructs as indicated.
(PDF)

S4 Fig Secondary structure predictions for XpsC, XpsL and XpsM from *X. euvesicatoria.
* Secondary structures were predicted using the AlphaFold2 algorithm and the molecular visualization program UCSF ChimeraX [40,41,71]. N- and C-terminal regions are indicated in blue and red colours, respectively. The models on the right side show the per-residue model confidence score (pLDDT, predicted local distance difference test) which is scaled from 0 to 100 with blue colours indicating higher scores (70 – 100) and thus a higher confidence and more accurate prediction.
(PDF)

S5 Fig Predicted secondary structures in XpsC and corresponding GspC proteins. (A) Predicted α-helices and β-sheets in XpsC from *X. euvesicatoria.* The amino acid sequence of XpsC (accession number CAJ25390) and the positions of predicted α helices and β sheets predicted by AlphaFold2 are shown [41,71]. Coloured rectangles refer to cytoplasmic, transmembrane and periplasmic regions as well as to the HR and 2P domains as indicated. Numbers indicate amino acid positions. (B) Predicted secondary structure elements in GspC proteins. α helices and β sheets were predicted and indicated as described in (A). The following proteins were analysed: XcpP from *P. aeruginosa* strain PAO1 (accession number CAA48581), OutC from *D. dadantii* strain 3937 (accession number CAA46369) and EpsC from *V. cholerae* strain N16961 (accession number P45777).
(PDF)

S6 Fig Structure predictions of assembly platform components from *X. euvesicatoria, P. aeruginosa, D. dadantii* and *V. cholerae.
* Complexes containing XpsCLM from *X. euvesicatoria* or corresponding proteins from *P. aeruginosa, D. dadantii* and *V. cholerae* were predicted using the AlphaFold2 algorithm and the molecular visualization program UCSF ChimeraX [40,41,71]. Similarly, XpsD as well as complexes between XpsL and XpsE, XpsC and the N-terminal region of XpsD (XpsD_2-252_) were modeled. In the models on the left side, proteins are shown in different colours as indicated. The models on the right side show the per-residue model confidence score (pLDDT, predicted local distance difference test) which is scaled from 0 to 100 with different colours referring to different scores as indicated. In addition, a predicted aligned error (PAE) plot shows regions of high (blue colour, low PAE value) and low (red colour, high PAE value) confidence for the predicted structures. pTM (predicted template modeling) and ipTM (inter-chain predicted template modeling) values are indicated.
(PDF)

S7 Fig Synthesis and immunological detection of T18 and T25 fusions of assembly platform components. Cell extracts from *E. coli* strain JM109 containing expression constructs encoding T18 and T25 fusions of XpsC, XpsL, XpsM and XpsE as indicated were analysed by immunoblotting, using a FLAG epitope-specific antibody.
(PDF)

S8 Fig Introduction of a nonsense mutation upstream of the start codon of *xpsC* prevents alternative translation initiation. (A) The *xpsG* promoter, which was used for the expression of *xpsC-c-myc*, contains several potential ATG or GTG start codons as indicated. To prevent the synthesis of XpsC-c-Myc with additional N-terminal amino acids, the expression construct encoding c-Myc-XpsC,contained a nonsense mutation (C to T exchange) upstream of the translation initiation start site as indicated. The mutated nucleotide is underlined, the start codon of *c-myc-xpsC* in shown in bold letters. (B) Detection of c-Myc-XpsC-containing complexes after *in vivo* crosslinking using the expression construct shown in (A). Derivatives of strain 85-10∆*xps* (∆*xps*) containing the wild-type (WT) modular T2S gene cluster (+pT2S) or a derivative thereof deleted in *xpsC* and encoding c-Myc-XpsC as indicated were grown in NYG medium. Equal amounts of cell cultures were centrifuged and cells were either resuspended in Laemmli buffer at 99°C (total extract) or incubated with formaldehyde and resuspended in Laemmli buffer at 37°C (crosslinked). Proteins were analysed by immunoblotting using antibodies specific for the c-Myc epitope or GroEL to ensure equal loading. Signals corresponding to c-Myc-XpsC and a c-Myc-XpsC-specific protein complex are indicated with a black arrow and an asterisk, respectively. For the analysis of T2S system activity, bacteria were grown on milk protein-containing agar plates to demonstrate extracellular protease activity. Halo formation was documented two days after incubation. Experiments were performed three times with similar results. One representative example is shown. The numbers refer to the width of the halos in millimeters, with mean values calculated from three replicates.
(PDF)

S9 Fig A C-terminally c-Myc epitope-tagged XpsD derivative is functional and forms protein complexes. (A) Complementation studies with XpsC-c-Myc. *Xe* strains 85-10 (WT) and 85-10∆*xpsD* (∆*xpsD*) with or without an expression construct encoding XpsD-c-Myc under control of the *XCV4361* promoter were inoculated into leaves of susceptible ECW (Early Cal Wonder) pepper plants. Disease symptoms were photographed 7 dpi. Dashed lines indicate the infiltrated areas. For the analysis of extracellular protease activity, bacteria were grown on milk protein-containing agar plates and halo formation was documented two days after incubation. Experiments were performed three times with similar results. When compared with the *lac* promoter, the *XCV4361* promoter results in lower expression levels [30,72]. (B) XpsC-c-Myc-specific complexes are detected in the absence of other T2S system components. Strains 85-10 (WT), 85-10∆*xpsE-D* (∆*xpsE-D*) and 85-10∆*xpsD* (∆*xpsD*) containing the XpsD-c-Myc expression construct (XpsD) as indicated were grown in minimal medium at pH 7.0. Equal amounts of bacterial cultures during the exponential growth phase were analysed by immunoblotting using a c-Myc epitope-specific antibody. The blot was reprobed with an antibody against GroEL to demonstrate equal loading. XpsD-c-Myc-specific signals, which likely correspond to oligomeric complexes, were detected in the stacking gel as indicated.
(PDF)

S10 Fig Localization of XopB-mCherry in *X. euvesicatoria.
* *X. euvesicatoria* strain 85*∆*hrp*_fs*HAGX* containing a modular T3S gene cluster encoding the type III effector XopB fused to mCherry was incubated in minimal medium under T3S-permissive conditions. mCherry fluorescence was analysed by fluorescence microscopy. One representative image is shown. The size bar corresponds to 2.5 µm. The picture in the right panel results from an overlay of the fluorescent signals with the images of the brightfield channel. The modular T3S gene cluster was previously generated using Golden Gate cloning and contains the *hrp* (hypersensitive response and pathogenicity) gene cluster and the accessory genes *xopA, hpaH, hrpG* and *hrpX.* A reporter fusion encoding, e.g., XopB-mCherry was included. The modular T3S gene cluster construct was analysed in a *X. euvesicatoria* strain deleted in the native *hrp* gene cluster (∆*hrp*) and containing frameshift (fs) mutations in *xopA, hpaH, hrpG* and *hrpX* (*HAGX*) [36]. XopB-mCherry does not form fluorescent foci and is detected in the bacterial cytoplasm. The cytoplasmic localization of mCherry was previously also reported for other *Xanthomonas* spp. [73].
(PDF)

## References

[1] Abby SS, Cury J, Guglielmini J, Néron B, Touchon M, Rocha EPC. Identification of protein secretion systems in bacterial genomes. Sci Rep. 2016;6:23080. doi: 10.1038/srep23080 26979785 PMC4793230

[2] Cianciotto NP. The type II secretion system as an underappreciated and understudied mediator of interbacterial antagonism. Infect Immun. 2024;92(8):e0020724. doi: 10.1128/iai.00207-24 38980047 PMC11320942

[3] Korotkov KV, Sandkvist M. Architecture, function, and substrates of the Type II secretion system. EcoSal Plus. 2019;8(2):10.1128/ecosalplus.ESP-0034–2018. doi: 10.1128/ecosalplus.ESP-0034-2018 30767847 PMC6638579

[4] Naskar S, Hohl M, Tassinari M, Low HH. The structure and mechanism of the bacterial type II secretion system. Mol Microbiol. 2021;115(3):412–24. doi: 10.1111/mmi.14664 33283907

[5] Cianciotto NP, White RC. Expanding role of Type II secretion in bacterial pathogenesis and beyond. Infect Immun. 2017;85(5):e00014-17. doi: 10.1128/IAI.00014-17 28264910 PMC5400843

[6] Costa TRD, Felisberto-Rodrigues C, Meir A, Prevost MS, Redzej A, Trokter M, et al. Secretion systems in Gram-negative bacteria: structural and mechanistic insights. Nat Rev Microbiol. 2015;13(6):343–59. doi: 10.1038/nrmicro3456 25978706

[7] Desvaux M, Parham NJ, Scott-Tucker A, Henderson IR. The general secretory pathway: a general misnomer?. Trends Microbiol. 2004;12(7):306–9. doi: 10.1016/j.tim.2004.05.002 15223057

[8] Gu S, Shevchik VE, Shaw R, Pickersgill RW, Garnett JA. The role of intrinsic disorder and dynamics in the assembly and function of the type II secretion system. Biochim Biophys Acta Proteins Proteom. 2017;1865(10):1255–66. doi: 10.1016/j.bbapap.2017.07.006 28733198

[9] Yan Z, Yin M, Xu D, Zhu Y, Li X. Structural insights into the secretin translocation channel in the type II secretion system. Nat Struct Mol Biol. 2017;24(2):177–83. doi: 10.1038/nsmb.3350 28067918

[10] Korotkov KV, Gonen T, Hol WGJ. Secretins: dynamic channels for protein transport across membranes. Trends Biochem Sci. 2011;36(8):433–43. doi: 10.1016/j.tibs.2011.04.002 21565514 PMC3155655

[11] Chernyatina AA, Low HH. Core architecture of a bacterial type II secretion system. Nat Commun. 2019;10(1):5437. doi: 10.1038/s41467-019-13301-3 31780649 PMC6882859

[12] Korotkov KV, Krumm B, Bagdasarian M, Hol WGJ. Structural and functional studies of EpsC, a crucial component of the type 2 secretion system from *Vibrio cholerae*. J Mol Biol. 2006;363(2):311–21. doi: 10.1016/j.jmb.2006.08.037 16978643

[13] Pineau C, Guschinskaya N, Robert X, Gouet P, Ballut L, Shevchik VE. Substrate recognition by the bacterial type II secretion system: more than a simple interaction. Mol Microbiol. 2014;94(1):126–40. doi: 10.1111/mmi.12744 25098941

[14] Dazzoni R, Li Y, López-Castilla A, Brier S, Mechaly A, Cordier F, et al. Structure and dynamic association of an assembly platform subcomplex of the bacterial type II secretion system. Structure. 2023;31(2):152–165.e7. doi: 10.1016/j.str.2022.12.003 36586404

[15] Li Y, Santos-Moreno J, Francetic O. The periplasmic coiled coil formed by the assembly platform proteins PulL and PulM is critical for function of the *Klebsiella* type II secretion system. Res Microbiol. 2023;174(7):104075. doi: 10.1016/j.resmic.2023.104075 37141929

[16] Thomassin J-L, Santos Moreno J, Guilvout I, Tran Van Nhieu G, Francetic O. The trans-envelope architecture and function of the type 2 secretion system: new insights raising new questions. Mol Microbiol. 2017;105(2):211–26. doi: 10.1111/mmi.13704 28486768

[17] Michel-Souzy S, Douzi B, Cadoret F, Raynaud C, Quinton L, Ball G, et al. Direct interactions between the secreted effector and the T2SS components GspL and GspM reveal a new effector-sensing step during type 2 secretion. J Biol Chem. 2018;293(50):19441–50. doi: 10.1074/jbc.RA117.001127 30337370 PMC6302157

[18] Ghosal D, Kim KW, Zheng H, Kaplan M, Truchan HK, Lopez AE, et al. *In vivo* structure of the *Legionella* type II secretion system by electron cryotomography. Nat Microbiol. 2019;4(12):2101–8. doi: 10.1038/s41564-019-0603-6 31754273 PMC6879910

[19] Barbat B, Douzi B, Ball G, Tribout M, El Karkouri K, Kellenberger C, et al. Insights into dynamics and gating properties of T2SS secretins. Sci Adv. 2023;9(40):eadg6996. doi: 10.1126/sciadv.adg6996 37792935 PMC10550240

[20] Douzi B, Ball G, Cambillau C, Tegoni M, Voulhoux R. Deciphering the Xcp *Pseudomonas aeruginosa* type II secretion machinery through multiple interactions with substrates. J Biol Chem. 2011;286(47):40792–801. doi: 10.1074/jbc.M111.294843 21949187 PMC3220450

[21] An S-Q, Potnis N, Dow M, Vorhölter F-J, He Y-Q, Becker A, et al. Mechanistic insights into host adaptation, virulence and epidemiology of the phytopathogen *Xanthomonas*. FEMS Microbiol Rev. 2020;44(1):1–32. doi: 10.1093/femsre/fuz024 31578554 PMC8042644

[22] Potnis N, Timilsina S, Strayer A, Shantharaj D, Barak JD, Paret ML, et al. Bacterial spot of tomato and pepper: diverse *Xanthomonas* species with a wide variety of virulence factors posing a worldwide challenge. Mol Plant Pathol. 2015;16(9):907–20. doi: 10.1111/mpp.12244 25649754 PMC6638463

[23] Alvarez-Martinez CE, Sgro GG, Araujo GG, Paiva MRN, Matsuyama BY, Guzzo CR, et al. Secrete or perish: The role of secretion systems in *Xanthomonas* biology. Comput Struct Biotechnol J. 2020;19:279–302. doi: 10.1016/j.csbj.2020.12.020 33425257 PMC7777525

[24] Timilsina S, Potnis N, Newberry EA, Liyanapathiranage P, Iruegas-Bocardo F, White FF, et al. *Xanthomonas* diversity, virulence and plant-pathogen interactions. Nat Rev Microbiol. 2020;18(8):415–27. doi: 10.1038/s41579-020-0361-8 32346148

[25] Thieme F, Koebnik R, Bekel T, Berger C, Boch J, Büttner D, et al. Insights into genome plasticity and pathogenicity of the plant pathogenic bacterium *Xanthomonas campestris* pv. vesicatoria revealed by the complete genome sequence. J Bacteriol. 2005;187(21):7254–66. doi: 10.1128/JB.187.21.7254-7266.2005 16237009 PMC1272972

[26] Büttner D, Bonas U. Regulation and secretion of *Xanthomonas* virulence factors. FEMS Microbiol Rev. 2010;34(2):107–33. doi: 10.1111/j.1574-6976.2009.00192.x 19925633

[27] Deng W, Marshall NC, Rowland JL, McCoy JM, Worrall LJ, Santos AS, et al. Assembly, structure, function and regulation of type III secretion systems. Nat Rev Microbiol. 2017;15(6):323–37. doi: 10.1038/nrmicro.2017.20 28392566

[28] Büttner D. Behind the lines-actions of bacterial type III effector proteins in plant cells. FEMS Microbiol Rev. 2016;40(6):894–937. doi: 10.1093/femsre/fuw026 28201715 PMC5091034

[29] Szczesny R, Jordan M, Schramm C, Schulz S, Cogez V, Bonas U, et al. Functional characterization of the Xcs and Xps type II secretion systems from the plant pathogenic bacterium *Xanthomonas campestris* pv. *vesicatoria*. New Phytol. 2010;187(4):983–1002. doi: 10.1111/j.1469-8137.2010.03312.x 20524995

[30] Solé M, Scheibner F, Hoffmeister A-K, Hartmann N, Hause G, Rother A, et al. *Xanthomonas campestris* pv. *vesicatoria* Secretes Proteases and Xylanases via the Xps Type II Secretion System and Outer Membrane Vesicles. J Bacteriol. 2015;197(17):2879–93. doi: 10.1128/JB.00322-15 26124239 PMC4524030

[31] Katsir L, Bahar O. Bacterial outer membrane vesicles at the plant-pathogen interface. PLoS Pathog. 2017;13(6):e1006306. doi: 10.1371/journal.ppat.1006306 28570683 PMC5453609

[32] Lee H-M, Chen J-R, Lee H-L, Leu W-M, Chen L-Y, Hu N-T. Functional dissection of the XpsN (GspC) protein of the *Xanthomonas campestris* pv. *campestris* type II secretion machinery. J Bacteriol. 2004;186(10):2946–55. doi: 10.1128/JB.186.10.2946-2955.2004 15126454 PMC400604

[33] Engler C, Kandzia R, Marillonnet S. A one pot, one step, precision cloning method with high throughput capability. PLoS One. 2008;3(11):e3647. doi: 10.1371/journal.pone.0003647 18985154 PMC2574415

[34] Weber E, Gruetzner R, Werner S, Engler C, Marillonnet S. Assembly of designer TAL effectors by Golden Gate cloning. PLoS One. 2011;6(5):e19722. doi: 10.1371/journal.pone.0019722 21625552 PMC3098256

[35] Werner S, Engler C, Weber E, Gruetzner R, Marillonnet S. Fast track assembly of multigene constructs using Golden Gate cloning and the MoClo system. Bioeng Bugs. 2012;3(1):38–43. doi: 10.4161/bbug.3.1.18223 22126803

[36] Hausner J, Jordan M, Otten C, Marillonnet S, Büttner D. Modular Cloning of the Type III Secretion Gene Cluster from the Plant-Pathogenic Bacterium *Xanthomonas euvesicatoria*. ACS Synth Biol. 2019;8(3):532–47. doi: 10.1021/acssynbio.8b00434 30694661

[37] Noël L, Thieme F, Nennstiel D, Bonas U. cDNA-AFLP analysis unravels a genome-wide *hrpG*-regulon in the plant pathogen *Xanthomonas campestris* pv. *vesicatoria*. Mol Microbiol. 2001;41(6):1271–81. doi: 10.1046/j.1365-2958.2001.02567.x 11580833

[38] Tsai R-T, Leu W-M, Chen L-Y, Hu N-T. A reversibly dissociable ternary complex formed by XpsL, XpsM and XpsN of the *Xanthomonas campestris* pv. *campestris* type II secretion apparatus. Biochem J. 2002;367(Pt 3):865–71. doi: 10.1042/BJ20020909 12123417 PMC1222915

[39] Lallemand M, Login FH, Guschinskaya N, Pineau C, Effantin G, Robert X, et al. Dynamic interplay between the periplasmic and transmembrane domains of GspL and GspM in the type II secretion system. PLoS One. 2013;8(11):e79562. doi: 10.1371/journal.pone.0079562 24223969 PMC3815138

[40] Goddard TD, Huang CC, Meng EC, Pettersen EF, Couch GS, Morris JH, et al. UCSF ChimeraX: Meeting modern challenges in visualization and analysis. Protein Sci. 2018;27(1):14–25. doi: 10.1002/pro.3235 28710774 PMC5734306

[41] Jumper J, Evans R, Pritzel A, Green T, Figurnov M, Ronneberger O, et al. Highly accurate protein structure prediction with AlphaFold. Nature. 2021;596(7873):583–9. doi: 10.1038/s41586-021-03819-2 34265844 PMC8371605

[42] Bleves S, Gérard-Vincent M, Lazdunski A, Filloux A. Structure-function analysis of XcpP, a component involved in general secretory pathway-dependent protein secretion in *Pseudomonas aeruginosa*. J Bacteriol. 1999;181(13):4012–9. doi: 10.1128/JB.181.13.4012-4019.1999 10383969 PMC93891

[43] Login FH, Fries M, Wang X, Pickersgill RW, Shevchik VE. A 20-residue peptide of the inner membrane protein OutC mediates interaction with two distinct sites of the outer membrane secretin OutD and is essential for the functional type II secretion system in *Erwinia chrysanthemi*. Mol Microbiol. 2010;76(4):944–55. doi: 10.1111/j.1365-2958.2010.07149.x 20444086

[44] Sheng M, Sala C. PDZ domains and the organization of supramolecular complexes. Annu Rev Neurosci. 2001;24:1–29. doi: 10.1146/annurev.neuro.24.1.1 11283303

[45] Murciano-Calles J. The Conformational Plasticity Vista of PDZ Domains. Life (Basel). 2020;10(8):123. doi: 10.3390/life10080123 32726937 PMC7460260

[46] Battesti A, Bouveret E. The bacterial two-hybrid system based on adenylate cyclase reconstitution in *Escherichia coli*. Methods. 2012;58(4):325–34. doi: 10.1016/j.ymeth.2012.07.018 22841567

[47] Karimova G, Pidoux J, Ullmann A, Ladant D. A bacterial two-hybrid system based on a reconstituted signal transduction pathway. Proc Natl Acad Sci U S A. 1998;95(10):5752–6. doi: 10.1073/pnas.95.10.5752 9576956 PMC20451

[48] Shiue S-J, Kao K-M, Leu W-M, Chen L-Y, Chan N-L, Hu N-T. XpsE oligomerization triggered by ATP binding, not hydrolysis, leads to its association with XpsL. EMBO J. 2006;25(7):1426–35. doi: 10.1038/sj.emboj.7601036 16525507 PMC1440322

[49] Hoffman EA, Frey BL, Smith LM, Auble DT. Formaldehyde crosslinking: a tool for the study of chromatin complexes. J Biol Chem. 2015;290(44):26404–11. doi: 10.1074/jbc.R115.651679 26354429 PMC4646298

[50] Tayri-Wilk T, Slavin M, Zamel J, Blass A, Cohen S, Motzik A, et al. Mass spectrometry reveals the chemistry of formaldehyde cross-linking in structured proteins. Nat Commun. 2020;11(1):3128. doi: 10.1038/s41467-020-16935-w 32561732 PMC7305180

[51] Meiresonne NY, van der Ploeg R, Hink MA, den Blaauwen T. Activity-related conformational changes in d,d-carboxypeptidases revealed by in vivo periplasmic förster resonance energy transfer assay in *Escherichia coli*. mBio. 2017;8(5):e01089-17. doi: 10.1128/mBio.01089-17 28900026 PMC5596342

[52] Buddelmeijer N, Krehenbrink M, Pecorari F, Pugsley AP. Type II secretion system secretin PulD localizes in clusters in the *Escherichia coli* outer membrane. J Bacteriol. 2009;191(1):161–8. doi: 10.1128/JB.01138-08 18978053 PMC2612452

[53] Weber E, Engler C, Gruetzner R, Werner S, Marillonnet S. A modular cloning system for standardized assembly of multigene constructs. PLoS One. 2011;6(2):e16765. doi: 10.1371/journal.pone.0016765 21364738 PMC3041749

[54] Michel G, Bleves S, Ball G, Lazdunski A, Filloux A. Mutual stabilization of the XcpZ and XcpY components of the secretory apparatus in *Pseudomonas aeruginosa*. Microbiology (Reading). 1998;144 ( Pt 12):3379–86. doi: 10.1099/00221287-144-12-3379 9884230

[55] Zhang S, Gu S, Rycroft P, Ruaudel F, Delolme F, Robert X, et al. Scaffolding protein GspB/OutB facilitates assembly of the *Dickeya dadantii* type 2 secretion system by anchoring the outer membrane secretin pore to the inner membrane and to the peptidoglycan cell wall. mBio. 2022;13(3):e0025322. doi: 10.1128/mbio.00253-22 35546537 PMC9239104

[56] Wang X, Pineau C, Gu S, Guschinskaya N, Pickersgill RW, Shevchik VE. Cysteine scanning mutagenesis and disulfide mapping analysis of arrangement of GspC and GspD protomers within the type 2 secretion system. J Biol Chem. 2012;287(23):19082–93. doi: 10.1074/jbc.M112.346338 22523076 PMC3365941

[57] Lee HM, Wang KC, Liu YL, Yew HY, Chen LY, Leu WM, et al. Association of the cytoplasmic membrane protein XpsN with the outer membrane protein XpsD in the type II protein secretion apparatus of *Xanthomonas campestris* pv. *campestris*. J Bacteriol. 2000;182(6):1549–57. doi: 10.1128/JB.182.6.1549-1557.2000 10692359 PMC94451

[58] Gu S, Kelly G, Wang X, Frenkiel T, Shevchik VE, Pickersgill RW. Solution structure of homology region (HR) domain of type II secretion system. J Biol Chem. 2012;287(12):9072–80. doi: 10.1074/jbc.M111.300624 22253442 PMC3308744

[59] Lu C, Korotkov KV, Hol WGJ. Crystal structure of the full-length ATPase GspE from the *Vibrio vulnificus* type II secretion system in complex with the cytoplasmic domain of GspL. J Struct Biol. 2014;187(3):223–35. doi: 10.1016/j.jsb.2014.07.006 25092625 PMC4150747

[60] Abendroth J, Mitchell DD, Korotkov KV, Johnson TL, Kreger A, Sandkvist M, et al. The three-dimensional structure of the cytoplasmic domains of EpsF from the type 2 secretion system of *Vibrio cholerae*. J Struct Biol. 2009;166(3):303–15. doi: 10.1016/j.jsb.2009.03.009 19324092 PMC2730350

[61] Lu C, Turley S, Marionni ST, Park Y-J, Lee KK, Patrick M, et al. Hexamers of the type II secretion ATPase GspE from *Vibrio cholerae* with increased ATPase activity. Structure. 2013;21(9):1707–17. doi: 10.1016/j.str.2013.06.027 23954505 PMC3775503

[62] Guilvout I, Samsudin F, Huber RG, Bond PJ, Bardiaux B, Francetic O. Membrane platform protein PulF of the *Klebsiella* type II secretion system forms a trimeric ion channel essential for endopilus assembly and protein secretion. mBio. 2024;15(1):e0142323. doi: 10.1128/mbio.01423-23 38063437 PMC10790770

[63] López-Castilla A, Thomassin J-L, Bardiaux B, Zheng W, Nivaskumar M, Yu X, et al. Structure of the calcium-dependent type 2 secretion pseudopilus. Nat Microbiol. 2017;2(12):1686–95. doi: 10.1038/s41564-017-0041-2 28993624 PMC5705324

[64] Py B, Loiseau L, Barras F. An inner membrane platform in the type II secretion machinery of Gram-negative bacteria. EMBO Rep. 2001;2(3):244–8. doi: 10.1093/embo-reports/kve042 11266368 PMC1083838

[65] Lybarger SR, Johnson TL, Gray MD, Sikora AE, Sandkvist M. Docking and assembly of the type II secretion complex of *Vibrio cholerae*. J Bacteriol. 2009;191(9):3149–61. doi: 10.1128/JB.01701-08 19251862 PMC2681814

[66] Schulze S, Kay S, Büttner D, Egler M, Eschen-Lippold L, Hause G, et al. Analysis of new type III effectors from *Xanthomonas* uncovers XopB and XopS as suppressors of plant immunity. New Phytol. 2012;195(4):894–911. doi: 10.1111/j.1469-8137.2012.04210.x 22738163

[67] Huguet E, Hahn K, Wengelnik K, Bonas U. *hpaA* mutants of *Xanthomonas campestris* pv. *vesicatoria* are affected in pathogenicity but retain the ability to induce host-specific hypersensitive reaction. Mol Microbiol. 1998;29(6):1379–90. doi: 10.1046/j.1365-2958.1998.01019.x 9781876

[68] Otten C, Büttner D. HrpB4 from *Xanthomonas campestris* pv. *vesicatoria* acts similarly to SctK proteins and promotes the docking of the predicted sorting platform to the type III secretion system. Cell Microbiol. 2021;23(6):e13327. doi: 10.1111/cmi.13327 33733571

[69] Karimova G, Dautin N, Ladant D. Interaction network among *Escherichia coli* membrane proteins involved in cell division as revealed by bacterial two-hybrid analysis. J Bacteriol. 2005;187(7):2233–43. doi: 10.1128/jb.187.7.2233-2243.2005 15774864 PMC1065216

[70] Skare JT, Ahmer BM, Seachord CL, Darveau RP, Postle K. Energy transduction between membranes. TonB, a cytoplasmic membrane protein, can be chemically cross-linked in vivo to the outer membrane receptor FepA. J Biol Chem. 1993;268(22):16302–8. doi: 10.1016/s0021-9258(19)85421-2 8344918

[71] Mirdita M, Schütze K, Moriwaki Y, Heo L, Ovchinnikov S, Steinegger M. ColabFold: making protein folding accessible to all. Nat Methods. 2022;19(6):679–82. doi: 10.1038/s41592-022-01488-1 35637307 PMC9184281

[72] Drehkopf S, Scheibner F, Büttner D. Functional characterization of VirB/VirD4 and Icm/Dot type IV secretion systems from the plant-pathogenic bacterium *Xanthomonas euvesicatoria*. Front Cell Infect Microbiol. 2023;13:1203159. doi: 10.3389/fcimb.2023.1203159 37593760 PMC10432156

[73] Pena MM, Teper D, Ferreira H, Wang N, Sato KU, Ferro MIT, et al. mCherry fusions enable the subcellular localization of periplasmic and cytoplasmic proteins in *Xanthomonas* sp. PLoS One. 2020;15(7):e0236185. doi: 10.1371/journal.pone.0236185 32730344 PMC7392301

