# Supplementary material for: Modularization of the type II secretion gene cluster from Xanthomonas euvesicatoria facilitates the identification of a structurally conserved XpsCLM assembly platform complex
Source: PLoS Pathog. 2025 Apr 9;21(4):e1013008. doi: 10.1371/journal.ppat.1013008 (PMC11981180; doi:10.1371/journal.ppat.1013008)
Supplement: S1 Table — (PDF) [file ppat.1013008.s001.pdf]

Table S1: Strains and plasmids used in this study.

Strain/ plasmid	Relevant characteristics[1]	Reference(s)
X. euvesicatoria		
85-10	Pepper race 2, wild type; Rif^R	Canteros, 1990; Kousik and Ritchie, 1998
85-10Δ*xps*	Derivative of strain 85-10 deleted in the entire *xps* gene cluster (nucleotide positions 4212581-4224331 of the genome)	This study
E. coli		
OneShot®TOP10	F^- *mcrA Δ(mrr-hsdRMS-mcrBC) φ80lacZΔM15 ΔlacX74 recA1 araΔ139 Δ(ara-leu)7697 galU galK rpsL endA1 nupG;* Str^R	Invitrogen
DH5alpha λpir	F^- recA hsdR17(r_{K}^-,m_{K}^+) φ80 ΔlacZ DM15 [λpir]	Ménard et al., 1993
JM109	F^-, *traD36 proA$^+$B$^+$ lacI^q Δ(lacZ)M15/ Δ(lac-proAB) glnV44 e14$^-$ gyrA96 recA1 relA1 endA1 thi hsdR17*	Yanisch-Perron et al., 1985
DHM1	F^-, *cya-854, recA1, endA1, gyrA96 (Nal^R), thi1, hsdR17, spoT1, rfbD1, glnV44*	Karimova et al., 2005
**Plasmids**		
pOGG2	Golden Gate-compatible derivative of suicide vector pOK1, *sacB sacQ mobRK2 oriR6K*; Sm^R	Schulze et al., 2012
pOGG2Δxps	Derivative of pOGG2 carrying flanking regions of the *xps* gene cluster including the upstream sequence from nucleotide positions 4224332 - 4225081 and the downstream sequence from nucleotide positions 4212580 – 4213329 of the genome	This study
pRK2013	ColE1 replicon, $TraRK^+$ Mob^+; Km^R	Figurski and Helinski, 1979
pAGM9121	Derivative of pUC19, contains *lacZα* fragment flanked by *Bpi*I sites , CTCA/CGAG overhangs; Sm^R	Addgene #51833; Weber et al., 2011
pUC57Δ*BsaI*	Derivative of pUC57 with *Bsa*I site mutated; Ap^R	Morbitzer et al., 2011
pUC57-$XCV4361_{prom}$	Derivative of pUC57Δ*BsaI* containing the putative promoter of XCV4361 flanked by *BsaI* sites with ATTC and TATG overhangs	Drehkopf et al., 2023
pICH41021	Derivative of pUC19 with mutated *Bsa*I site; Ap^R	Gift from S. Marillonnet
pAGM9121-xpsM	Derivative of pAGM9121 containing *xpsM* flanked by *Bsa*I sites, TATG/GGTG overhangs	This study
pICH41021-xpsC	Derivative of pICH41021 containing *xpsC* flanked by *Bsa*I sites, TATG/GGTG overhangs	This study
pICH41021-xpsL	Derivative of pICH41021 containing *xpsL* flanked by *Bsa*I sites, TATG/GGTG overhangs	This study
pICH41021-xpsF	Derivative of pICH41021 containing *xpsF* flanked by *Bsa*I sites, TATG/GGTG overhangs	This study
pICH41021-$xpsD_{1-527}$	Derivative of pICH41021 containing the first 527 codons of *xpsD* flanked by *Bsa*I sites, TATG/GCTG overhangs	This study
pICH41021-$xpsD_{528-764}$	Derivative of pICH41021 containing the codons 528 - 764 of *xpsD* flanked by *Bsa*I sites, GCTG/GGTG overhangs	This study
pUC57-xpsE	Derivative of pUC57 containing *xpsE* flanked by *Bsa*I sites, TATG/GGTG overhangs	This study
$pKT25_{GG}$	Golden Gate-compatible derivative of pKT25 encoding the T25 fragment of CyaA downstream of a *lac* promoter and in frame with a C-terminal FLAG epitope-encoding sequence; contains *lacP-eforRed* flanked by *Bsa*I sites downstream of the T25-FLAG fragment, TATG/GGTG overhangs; Km^R	Otten and Büttner, 2021

Strain/ plasmid	Relevant characteristics[1]	Reference(s)
pKNT25$_{GG}$	Golden Gate-compatible derivative of pKNT25, encodes the T25 fragment of CyaA in frame with an N-terminal FLAG epitope-encoding sequence downstream of the *lac* promoter, contains *lacP-eforRed* flanked by *Bsa*I sites upstream of the T25-FLAG fragment, TATG/GGTG overhangs; Km^R	Otten and Büttner, 2021
pUT18$_{GG}$	Golden Gate-compatible derivative of pUT18 containing *lacP-eforRed* flanked by *Bsa*I sites upstream of the FLAG-T18 fragment, TATG/GGTG overhangs; Gm^R	Otten and Büttner, 2021
pUT18C$_{GG}$	Golden Gate-compatible derivative of pUT18C containing *lacP-eforRed* flanked by *Bsa*I sites downstream of the T18-FLAG fragment, TATG/GGTG overhangs; Gm^R	Otten and Büttner, 2021
pUT18C$_{GG}$-xpsC	Derivative of pUT18C$_{GG}$ encoding T18-FLAG-XpsC	This study
pKT25$_{GG}$-xpsC	Derivative of pKT25$_{GG}$ encoding T25-FLAG-XpsC	This study
pUT18C$_{GG}$-xpsM	Derivative of pUT18C$_{GG}$ encoding T18-FLAG-XpsM	This study
pKT25$_{GG}$-xpsM	Derivative of pKT25$_{GG}$ encoding T25-FLAG-xpsM	This study
pUT18C$_{GG}$-xpsL	Derivative of pUT18C$_{GG}$ encoding T18-FLAG-XpsL	This study
pKT25$_{GG}$-xpsL	Derivative of pKT25$_{GG}$ encoding T25-FLAG-XpsL	This study
pUT18$_{GG}$-xpsE	Derivative of pUT18$_{GG}$ encoding XpsE-FLAG-T18	This study
pUT18C$_{GG}$-xpsE	Derivative of pUT18C$_{GG}$ encoding T18-FLAG-XpsE	This study
pKT25$_{GG}$-xpsE	Derivative of pKT25$_{GG}$ encoding T25-FLAG-XpsE	This study
pKNT25$_{GG}$-xpsE	Derivative of pKNT25$_{GG}$ encoding XpsE-FLAG-T25	This study
pBRM	Golden Gate-compatible derivative of pBBR1MCS-5 containing the *lac* promoter, a *lacZα* fragment flanked by *Bsa*I recognition sites and a 3 × c-Myc epitope-encoding sequence, TATG/GGTG overhangs; Gm^R	Szczesny et al., 2010
pBRNM	Derivative of pBRM containing the *lac* promoter, an N-terminal 3 × c-Myc epitope-encoding sequence and a *lacZα* fragment flanked by *Bsa*I recognition sites, TATG/GGTG overhangs, Gm^R	
pBRNM-xpsL	Derivative of pBRNM encoding 3×c-Myc-XpsL under control of the *lac* promoter; Gm^R	This study
pBRM-P-stop	Derivative of pBRM lacking the *lac* promoter, contains a stop codon upstream of the 3× c-Myc epitope-encoding sequence, ATTC/GGTG overhangs; Gm^R	Szczesny et al., 2010
pBRM-P-XCV4361$_{prom}$-xpsD	Derivative of pBRM-P encoding XpsD-c-Myc under control of the *XCV4361* promoter	This study
pUC57-ptac-GST	Derivative of pUC57 encoding GST downstream of the *tac* promoter flanked by *Bsa*I sites with ATTC/CATA overhangs; Ap^R	Drehkopf et al., 2020
pGEX-2TKM	derivative of *gst* expression vector pGEX-2TK with polylinker of pDSK604, p$_{tac}$ GST *lacI*^{q} pBR322 *ori;* Ap^R	Stratagene; Escolar et al., 2001
pB-P-stop-ptacGST-xpsE	Derivative of pBRM-P-stop encoding GST-XpsE under control of a *ptac* promoter; Gm^R	This study
**MoClo plasmids**		
pAGM1311	pUC19-derived level -1 vector, *lacZα* fragment flanked by *Bsa*I sites, ACAT/TTGT overhangs; Km^R	Addgene #51833; Weber et al., 2011
pICH41331	pUC19-derived level 0 vector, *lacZα* fragment flanked by *Bpi*I sites, GGAG/CGCT overhangs; Sm^R	Addgene #47999; Weber et al., 2011
pICH41295	pUC19-derived level 0 vector, *lacZα* fragment flanked by BpiI sites, GGAG/AATG overhangs; Sm^R	Addgene #47997 Weber et al., 2011
pICH41308	pUC19-derived level 0 vector, *lacZα* fragment flanked by *Bpi*I sites, AATG/GCTT overhangs; Sm^R	Addgene #47998; Weber et al., 2011

pICH47732	Level 1 destination vector derived from pBIN19 and pUC19, *lacZα* fragment flanked by *Bsa*I- (GGAG/CGCT overhangs) and *Bpi*I sites (AATG/GCTT overhangs), for level M position 1; Ap^R	Addgene #48000; Weber et al., 2011
pICH47742	Level 1 destination vector derived from pBIN19 and pUC19, *lacZα* fragment flanked by *Bsa*I- (GGAG/CGCT overhangs) and *Bpi*I sites (GCAA/ACTA overhangs), for level M position 2; Ap^R	Addgene #48001; Weber et al., 2011
pICH47751	Level 1 destination vector derived from pBIN19 and pUC19, *lacZα* fragment flanked by *Bsa*I (GGAG/CGCT overhangs) and *Bpi*I sites (ACTA/TTAC overhangs), for level M position 3; Ap^R	Addgene #48002; Weber et al., 2011
pICH47761	Level 1 destination vector derived from pBIN19 and pUC19, *lacZα* fragment flanked by *Bsa*I (GGAG/CGCT overhangs) and *Bpi*I sites (TTAC/CAGA overhangs), for level M position 4; Ap^R	Addgene #48003; Weber et al., 2011
pICH47772	Level 1 destination vector derived from pBIN19 and pUC19, *lacZα* fragment flanked by *Bsa*I (GGAG/CGCT overhangs) and *Bpi*I sites (CAGA/TGTG overhangs), for level M position 5; Ap^R	Addgene #48004; Weber et al., 2011
pAGM8031	Level M vector derived from pBIN19 and pUC19, *lacZα* fragment flanked by *Bpi*I sites, TGCC/GGGA overhangs, Sm^R	Addgene #48037; Weber et al., 2011
pICH54011	Dummy module derived from pBIN19 and pUC19, 15-bp insert for level M position 1 with TGCC/GCAA *Bpi*I fusion sites, Ap^R	Addgene #48065; Weber et al., 2011
pICH54022	Dummy module derived from pBIN19 and pUC19, 15-bp insert for level M position 2 with GCAA/ACTA *Bpi*I fusion sites; Ap^R	Addgene #48066; Weber et al., 2011
pICH54033	Dummy module derived from pBIN19 and pUC19, 15-bp insert for level M position 3 with ACTA/TTAC *Bpi*I fusion sites; Ap^R	Addgene #48067; Weber et al., 2011
pICH54044	Dummy module derived from pBIN19 and pUC19, 15-bp insert for level M position 4 with TTAC/CAGA *Bpi*I fusion sites; Ap^R	Addgene #48068; Weber et al., 2011
pICH54055	Dummy module derived from pBIN19 and pUC19, 15-bp insert for level M position 5 with CAGA/TGTG *Bpi*I fusion sites; Ap^R	Addgene #48069; Weber et al., 2011
pICH50872	Level M end-linker for position 6; derived from pUC19, GCAA/- *Bsa*I and GCAA/GGGA *Bpi*I fusion sites; Ap^R	Addgene #48044; Weber et al., 2011
pICH50914	Level M end-linker for position 6; derived from pUC19, TGTG/- *Bsa*I and TGTG/GGGA *Bpi*I fusion sites; Ap^R	Addgene #48048; Weber et al., 2011
**Constructs for the generation of the modular *xps* gene cluster**		
Level -2 constructs		
pT2S011	Derivative of pAGM9121 containing the *xpsG* promoter (213 bp upstream of *xpsG)*; Sm^R	This study
pT2S012	Derivative of pAGM9121 containing *xpsG*; Sm^R	This study
pT2S013	Derivative of pAGM9121 containing *xpsH*; Sm^R	This study
pT2S014	Derivative of pAGM9121 containing *xpsI*; Sm^R	This study
pT2S016	Derivative of pAGM9121 containing bp 1-429 of *xpsJ*; Sm^R	This study
pT2S017	Derivative of pAGM9121 containing bp 426-636 of *xpsJ*; Sm^R	This study
pT2S018	Derivative of pAGM9121 containing *xpsK*; Sm^R	This study
pT2S019	Derivative of pAGM9121 containing bp 1-449 of *xpsL*; Sm^R	This study

pT2S020	Derivative of pAGM9121 containing bp 446-1122 of *xpsL*; Sm^R	This study
pT2S022	Derivative of pAGM9121 containing bp 14-654 of *xpsM*; Sm^R	This study
pT2S023	Derivative of pAGM9121 containing bp 8-227 of *xpsC*; Sm^R	This study
pT2S024	Derivative of pAGM9121 containing bp 223-795 of *xpsC*; Sm^R	This study
pT2S025	Derivative of pAGM9121 containing bp 1-1572 of *xpsD*; Sm^R	This study
pT2S026	Derivative of pAGM9121 containing bp 1568-2292 of *xpsD*; Sm^R	This study
Level -1		
pT2S001	Derivative of pAGM1311 containing *xpsE* and the *xpsE* promoter (365 bp upstream of *xpsE)*; Km^R	This study
pT2S005	Derivative of pAGM1311 containing *xpsF* and the *xpsF* promoter (175 bp upstream of *xpsF)*; Km^R	This study
pT2S015	Derivative of pAGM1311 containing *xpsG*, *xpsH, xpsI* downstream of the *xpsG* promoter; Km^R	This study
pT2S021	Derivative of pAGM1311 containing *xpsJ*, *xpsK* and *xpsL*; Km^R	This study
pT2S028	Derivative of pAGM1311 containing *xpsM*, *xpsC* and *xpsD*; Km^R	This study
Level 0		
pT2S002	Derivative of pICH41331 containing *xpsE* downstream of the *xpsE* promoter; Sm^R	This study
pT2S006	Derivative of pICH41331 containing *xpsF* downstream of the *xpsF* promoter; Sm^R	This study
pT2S030	Derivative of pICH41331 containing the *xpsG* - *xpsD* operon downstream of the *xpsG* promoter; Sm^R	This study
Level 1		
pT2S032	Derivative of pICH47742 containing *xpsE* downstream of the *xpsE* promoter; Ap^R	This study
pT2S034	Derivative of pICH47751 containing *xpsF* downstream of the *xpsF* promoter; Ap^R	This study
pT2S036	Derivative of pICH47761 containing the *xpsG* - *xpsD* operon downstream of the *xpsG* promoter; Ap^R	This study
Level M		
pT2S038	Derivative of pAGM8031 containing *xpsE* - *xpsD* with native promoters; Sm^R	This study
**Constructs for deletion of *xps* genes**		
Level -2		
pT2S091	Derivative of pAGM9121 encoding amino acids 259 - 373 of XpsL and XpsM; Sm^R	This study
pT2S096	Derivative of pAGM9121 encoding XpsK and amino acids 1 - 32 of XpsL; Sm^R	This study
pT2S174	Derivative of pAGM9121 encoding amino acids 1 - 146 of XpsL with codon 147 mutated to a stop ($XpsL_{D147stop}$); Sm^R	This study
pT2S097	Derivative of pAGM9121 encoding amino acids 149 - 373 of XpsL and amino acids 1 - 33 of XpsM; Sm^R	This study
pT2S092	Derivative of pAGM9121 encoding amino acids 179 - 217 of XpsM and XpsC; Sm^R	This study
pT2S051	Derivative of pAGM9121 encoding amino acids 162 - 265 of XpsC; Sm^R	This study
pT2S056	Derivative of pAGM9121 encoding amino acids 1 - 102 of XpsD; Sm^R	This study

pT2S175	Derivative of pAGM9121 encoding amino acids 179 - 217 of XpsM and $XpsC_{\Delta 73\text{-}162}$; Sm^R	This study
Level -1		
pT2S103	Derivative of pAGM1311 encoding XpsJ, XpsK and amino acids 1 - 32 of XpsL; Km^R	This study
pT2S099	Derivative of pAGM1311 encoding amino acids 259 - 373 of XpsL, XpsM, XpsC and XpsD; Km^R	This study
pT2S178	Derivative of pAGM1311 encoding XpsJ, XpsK and $XpsL_{D147stop}$; Km^R	This study
pT2S104	Derivative of pAGM1311 encoding XpsJ, XpsK, XpsL and amino acids 1 - 33 of XpsM; Km^R	This study
pT2S100	Derivative of pAGM1311 encoding amino acids 179 - 217 of XpsM, XpsC and XpsD; Km^R	This study
pT2S052	Derivative of pAGM1311 encoding XpsM, $XpsC_{\Delta 73\text{-}162}$ and XpsD; Km^R	This study
pT2S57	Derivative of pAGM1311 encoding XpsM, XpsC and $XpsD_{\Delta 103\text{-}523}$; Km^R	This study
pT2S179	Derivative of pAGM1311 encoding amino acids 179 - 217 of XpsM, $XpsC_{\Delta 73\text{-}162}$ and XpsD; Km^R	This study
pT2S188	Derivative of pAGM1311 encoding XpsJ, XpsK, $XpsL_{D147stop}$ and amino acids 1 - 33 of XpsM; Km^R	This study
pT2S137	Derivative of pAGM1311 encoding XpsM, $XpsC_{\Delta 73\text{-}162}$ and $XpsD_{\Delta 103\text{-}523}$; Km^R	This study
pT2S065	Derivative of pAGM1311 encoding XpsC for the generation of N-terminal fusions; Km^R	This study
Level 0		
pT2S107	Derivative of pICH41331 encoding XpsG, H, I, J, K, $L_{\Delta 33\text{-}258}$, M, C and D under control of the *xpsG* promoter; Sm^R	This study
pT2S187	Derivative of pICH41331 encoding XpsG, H, I, J, K, $XpsL_{D147stop}$, M, C and D under control of the *xpsG* promoter; Sm^R	This study
pT2S108	Derivative of pICH41331 encoding XpsG, H, I, J, K, L, $M_{\Delta 34\text{-}178}$, C and D under control of the *xpsG* promoter; Sm^R	This study
pT2S053	Derivative of pICH41331 encoding XpsG, H, I, J, K, L, M, $C_{\Delta 73\text{-}162}$ and D under control of the *xpsG* promoter; Sm^R	This study
pT2S058	Derivative of pICH41331 encoding XpsG, H, I, J, K, L, M, C and $XpsD_{\Delta 103\text{-}523}$ under control of the *xpsG* promoter; Sm^R	This study
pT2S170	Derivative of pICH41331 encoding XpsG, H, I, J, K, XpsL, M, $C_{\Delta 73\text{-}162}$ and $XpsD_{\Delta 103\text{-}523}$ under control of the *xpsG* promoter; Sm^R	This study
pT2S180	Derivative of pICH41331 encoding XpsG, H, I, J, K, L, $M_{\Delta 34\text{-}178}$, $C_{\Delta 73\text{-}162}$, D under control of the *xpsG* promoter; Sm^R	This study
pT2S181	Derivative of pICH41331 encoding XpsG, H, I, J, K, $XpsL_{D147stop}$, M, $C_{\Delta 73\text{-}162}$, D under control of the *xpsG* promoter; Sm^R	This study
pT2S182	Derivative of pICH41331 encoding XpsG, H, I, J, K, $XpsL_{D147stop}$, M, C and $XpsD_{\Delta 103\text{-}523}$ under control of the *xpsG* promoter; Sm^R	This study
pT2S193	Derivative of pICH41331 encoding XpsG, H, I, J, K, $XpsL_{D147stop}$, $M_{\Delta 34\text{-}178}$, C and D under control of the *xpsG* promoter; Sm^R	This study
pT2S294	Derivative of pICH41308 encoding c-Myc-XpsC; Sm^R	This study

pT2S352	Derivative of pICH41295 containing 209 bp of the *xpsG* promoter with a nonsense mutation at position -21 to prevent translation of additional upstream start codon; Sm^R	This study
Level1		
pT2S126	Derivative of pICH47761 encoding XpsG, H, I, J, K, $L_{Δ33-258}$, M, C and D under control of the *xpsG* promoter; Ap^R	This study
pT2S192	Derivative of pICH47761 encoding XpsG, H, I, J, K, $XpsL_{D147stop}$, M, C and D under control of the *xpsG* promoter; Ap^R	This study
pT2S127	Derivative of pICH47761 encoding XpsG, H, I, J, K, L, $M_{Δ34-178}$, C and D under control of the *xpsG* promoter; Ap^R	This study
pT2S054	Derivative of pICH47761 encoding XpsG, H, I, J, K, L, M, $C_{Δ73-162}$ and D under control of the *xpsG* promoter; Ap^R	This study
pT2S059	Derivative of pICH47761 encoding XpsG, H, I, J, K, L, M, C and $XpsD_{Δ103-523}$ under control of the *xpsG* promoter; Ap^R	This study
pT2S172	Derivative of pICH47761 encoding XpsG, H, I, J, K, XpsL, M, $C_{Δ73-162}$ and $XpsD_{Δ103-523}$ under control of the *xpsG* promoter; Ap^R	This study
pT2S189	Derivative of pICH47761 encoding XpsG, H, I, J, K, L, $M_{Δ34-178}$, $C_{Δ73-162}$ and D under control of the *xpsG* promoter; Ap^R	This study
pT2S194	Derivative of pICH47761 encoding XpsG, H, I, J, K, $XpsL_{D147stop}$, M, $C_{Δ73-162}$, D under control of the *xpsG* promoter; Ap^R	This study
pT2S190	Derivative of pICH47761 encoding XpsG, H, I, J, K, $XpsL_{D147stop}$, M, C and $XpsD_{Δ103-523}$ under control of the *xpsG* promoter; Ap^R	This study
pT2S198	Derivative of pICH47761 encoding XpsG, H, I, J, K, $XpsL_{D147stop}$, $M_{Δ34-178}$, C and D under control of the *xpsG* promoter; Ap^R	This study
pT2S495	Derivative of pICH47732 encoding 4×c-Myc-XpsC under control of the modified *xpsG* promoter without additional ORFs; Ap^R	This study
Level M		
pT2S069	Derivative of pAGM8031 encoding XpsF and XpsG, H, I, J, K, L, M, C and D with native promoters; Sm^R	This study
pT2S085	Derivative of pAGM8031 encoding XpsE and XpsG, H, I, J, K, L, M, C and D with native promoters; Sm^R	This study
pT2S156	Derivative of pAGM8031 encoding XpsE, XpsF and XpsG, H, I, J, K, $L_{Δ33-258}$, M, C and D with native promoters; Sm^R	This study
pT2S200	Derivative of pAGM8031 encoding XpsE, XpsF and XpsG, H, I, J, K, $XpsL_{D147stop}$, M, C and D with native promoters; Sm^R	This study
pT2S138	Derivative of pAGM8031 encoding XpsE, XpsF and XpsG, H, I, J, K, L, $M_{Δ34-178}$, C and D with native promoters; Sm^R	This study
pT2S055	Derivative of pAGM8031 encoding XpsE, XpsF and XpsG, H, I, J, K, L, M, $C_{Δ73-162}$ and D with native promoters; Sm^R	This study
pT2S060	Derivative of pAGM8031 encoding XpsE, XpsF and XpsG, H, I, J, K, L, M, C and $XpsD_{Δ103-523}$ with native promoters; Sm^R	This study

pT2S503	Level M module with the *xps* gene cluster with a deletion in *xpsC* and encoding 4×c-Myc-XpsC outside the *xps* gene cluster under control of the modified *xpsG* promoter; Sm^R	This study
Level P		
pAGB1136	Level P module containing the native T3S gene cluster, the accessory genes *xopA, hpaH, hrpG, hrpX* and *xopB-mCherry* under control of the *lac* promoter; Km^R	Hausner et al., 2019; C. Otten & D. Büttner unpublished
**Constructs for complementation studies**		
Level -1		
pT2S003	Derivative of pAGM1311 encoding XpsE-FLAG under control of the *xpsE* promoter; Km^R	This study
pT2S044	Derivative of pAGM1311 containing 209 bp of the *xpsG* promoter; Km^R	This study
pT2S045	Derivative of pAGM1311 encoding XpsC; Km^R	This study
pT2S167	Derivative of pAGM1311 encoding $XpsC_{\Delta 234\text{-}265}$; Km^R	This study
Level 0		
pT2S004	Derivative of pICH41331 encoding XpsE-FLAG under control of the *xpsE* promoter; Sm^R	This study
pT2S075	Derivative of pICH41331 containing 209 bp of the *xpsG* promoter; Sm^R	This study
pT2S119	Derivative of pAGM9121 encoding XpsL; Sm^R	This study
pT2S203	Derivative of pAGM9121 containing *xpsL* and 21 upstream bp encompassing the native *xpsL* Shine Dalgarno sequence; Sm^R	This study
pT2S114	Derivative of pAGM9121 encoding XpsM; Sm^R	This study
pT2S046	Derivative of pICH41331 encoding XpsC under control of the *xpsG* promoter; Sm^R	This study
pT2S209	Derivative of pAGM9121 encoding XpsD; Sm^R	This study
Level 1		
pT2S072	Derivative of pICH47732 encoding XpsE-FLAG under control of the *xpsE* promoter; Ap^R	This study
pT2S084	Derivative of pICH47732 encoding XpsF under control of the *xpsF* promoter; Ap^R	This study
pT2S135	Derivative of pICH47732 encoding XpsL under control of the *xpsG* promoter; Ap^R	This study
pT2S206	Derivative of pICH47732 containing *xpsL* and 21 upstream bp encompassing the native *xpsL* Shine Dalgarno sequence under control of the *xpsG* promoter; Ap^R	This study
pT2S125	Derivative of pICH47732 encoding XpsM under control of the *xpsG* promoter; Ap^R	This study
pT2S049	Derivative of pICH47732 encoding XpsC under control of the *xpsG* promoter; Ap^R	This study
pT2S225	Derivative of pICH47732 encoding XpsD under control of the *xpsG* promoter; Ap^R	This study
pT2S171	Derivative of pICH47732 encoding $XpsC_{\Delta 234\text{-}265}$ under control of the *xpsG* promoter; Ap^R	This study
Level M		
pT2S077	Derivative of pAGM8031 encoding XpsF and XpsG, H, I, J, K, L, M, C and D under control of native promoters and XpsE-FLAG under control of the native promoter inserted outside the *xps*-gene cluster; Sm^R	This study

pT2S086	Derivative of pAGM8031 encoding XpsE and XpsG, H, I, J, K, L, M, C and D under control of native promoters and XpsF under control of the native promoter inserted outside the *xps* gene cluster; Sm^R	This study
pT2S157	Derivative of pAGM8031 encoding XpsE, XpsF and XpsG, H, I, J, K, $L_{\Delta 33\text{-}258}$, M, C and D under control of native promoters and encoding XpsL under control of the native promoter outside the *xps* gene cluster; Sm^R	This study
pT2S208	Derivative of pAGM8031 encoding XpsE, XpsF and XpsG, H, I, J, K, $L_{\Delta 33\text{-}258}$, M, C and D under control of native promoters and encoding XpsL under control of the native promoter containing the *xpsL* Shine Dalgarno sequence outside the *xps* gene cluster; Sm^R	This study
pT2S201	Derivative of pAGM8031 encoding XpsE, XpsF and XpsG, H, I, J, K, $XpsL_{D147stop}$, M, C and D under control of native promoters and encoding XpsL under control of the native promoter outside the *xps* gene cluster; Sm^R	This study
pT2S207	Derivative of pAGM8031 encoding XpsE, XpsF and XpsG, H, I, J, K, $XpsL_{D147stop}$, M, C and D under control of native promoters and encoding XpsL under control of the native promoter containing the *xpsL* Shine Dalgarno sequence outside the *xps* gene cluster; Sm^R	This study
pT2S158	Derivative of pAGM8031 encoding XpsE, XpsF and XpsG, H, I, J, K, L, $M_{\Delta 34\text{-}178}$, C and D under control of native promoters and encoding XpsM under control of the native promoter outside the *xps* gene cluster; Sm^R	This study
pT2S073	Derivative of pAGM8031 encoding XpsE, XpsF and XpsG, H, I, J, K, L, M, $C_{\Delta 73\text{-}162}$ and D under control of native promoters and encoding XpsC under control of the native promoter outside the *xps* gene cluster; Sm^R	This study
pT2S230	Derivative of pAGM8031 encoding XpsE, XpsF and XpsG, H, I, J, K, L, M, C and $XpsD_{\Delta 103\text{-}523}$ under control of native promoters and encoding XpsD under control of the native promoter outside the *xps* gene cluster; Sm^R	This study
pT2S176	Derivative of pAGM8031 encoding XpsE, XpsF and XpsG, H, I, J, K, L, M, $C_{\Delta 73\text{-}162}$ and D under control of native promoters and encoding $XpsC_{\Delta 234\text{-}265}$ under control of the native promoter outside the *xps* gene cluster; Sm^R	This study
**Gene fusions with epitope-encoding sequences**		
Level -1		
pAGB1000	Derivative of pICH41021 containing a linker (2× AKLEGPAGL)-encoding sequence; Ap^R	Otten et al., 2021
pAGB1048	Derivative of pICH41021 containing *mcherry* for generation of C-terminal translational fusions; Ap^R	Drehkopf et al., 2022
pT2S210	Derivative of pAGM1311 encoding XpsD lacking a stop codon for the generation of C-terminal translational fusions; Km^R	This study
pICSL50010	Derivative of pAGM1301 encoding a C terminal 4×c-Myc epitope, Sm^R	Addgene #50310; Engler et al., 2014
pAGB872	Derivative of pICH41021 encoding a 4×c-Myc epitope for generation of N-terminal fusions; Ap^R	This study
pAGB873	Derivative of pICH41021 encoding a 4×c-Myc epitope for generation of C-terminal fusions; Ap^R	This study
pT2S066	Derivative of pAGM1311 encoding XpsC lacking a stop codon for the generation of C-terminal fusions; Km^R	This study

pT2S205	Derivative of pAGM1311 encoding XpsL for the generation of N-terminal fusions; Km^R	This study
Level 0		
pT2S219	Derivative of pICH41308 encoding XpsD-2×AKLEGPAGL-mCherry; Sm^R	This study
pAGB232	Derivative of pICH41276 containing a transcriptional terminator from *X. euvesicatoria*; Sm^R	Drehkopf et al., 2022
pT2S285	Derivative of pUC57 containing 209 bp of the *xpsG* promoter followed by 21 bp upstream region of *xpsL* encompassing the native *xpsL* Shine Dalgarno sequence; Ap^R	This study
pT2S289	derivative of pICH41308 encoding 4×c-Myc-XpsL; Sm^R	This study
pT2S293	derivative of pICH41308 encoding XpsC-4×c-Myc; Sm^R	This study
Level 1		
pT2S236	Derivative of pICH47732 encoding XpsD-2×AKLEGPAGL-mCherry under control of the *xpsG* promoter; Ap^R	This study
pT2S290	Derivative of pICH47732 encoding 4×c-Myc-XpsL under control of the *xpsG* promoter with the native *xpsL* Shine Dalgarno sequence; Ap^R	This study
pT2S301	Derivative of pICH47732 encoding XpsC-4×c-Myc under control of the *xpsG* promoter; Ap^R	This study
Level M		
pT2S238	Derivative of pAGM8031 encoding XpsE, XpsF and XpsG, H, I, J, K, L, M, C and $XpsD_{\Delta103\text{-}523}$ under control of native promoters and encoding XpsD-mCherry under control of the native promoter outside the *xps* gene cluster; Sm^R	This study
pT2S253	Derivative of pAGM8031 encoding XpsD-mCherry under control of the native promoter; Sm^R	This study
pT2S296	Derivative of pAGM8031 encoding XpsE, XpsF and XpsG, H, I, J, K, $XpsL_{D147stop}$, M, C and D under control of the native promoters and encoding 4×c-Myc-XpsL under control of the native promoter and containing the *xpsL* Shine Dalgarno sequence outside the *xps* gene cluster; assembled with pT2S290, pT2S032, pT2S034, pT2S192, pICH54055, pICH50914 and pAGM8031; Sm^R	This study
pT2S318	Derivative of pAGM8031 encoding 4×c-Myc-XpsL under control of the native promoter and containing the *xpsL* Shine Dalgarno sequence; assembled with pT2S290, pICH50872 and pAGM8031; Sm^R	This study
pT2S297	Derivative of pAGM8031 encoding XpsE, XpsF and XpsG, H, I, J, K, $XpsL_{D147stop}$, $M_{\Delta34\text{-}178}$, C and D under control of the native promoters and encoding 4×c-Myc-XpsL under control of the native promoter and containing the *xpsL* Shine Dalgarno sequence outside the *xps*-gene cluster; assembled with pT2S290, pT2S032, pT2S034, pT2S198, pICH54055, pICH50914 and pAGM8031; Sm^R	This study
pT2S298	Derivative of pAGM8031 encoding XpsE, XpsF and XpsG, H, I, J, K, $XpsL_{D147stop}$, M, $C_{\Delta73\text{-}162}$ and D under control of the native promoters and encoding 4×c-Myc-XpsL under control of the native promoter and containing the *xpsL* Shine Dalgarno sequence outside the *xps* gene cluster; assembled with pT2S290, pT2S032, pT2S034, pT2S194, pICH54055, pICH50914 and pAGM8031; Sm^R	This study

pT2S299	Derivative of pAGM8031 encoding XpsF and XpsG, H, I, J, K, XpsL$_{D147stop}$, M, C and D under control of the native promoters and encoding 4×c-Myc-XpsL under control of the native promoter and containing the *xpsL* Shine Dalgarno sequence outside the *xps* gene cluster, assembled with pT2S290, pICH54022, pT2S034, pT2S192, pICH54055, pICH50914 and pAGM8031; Sm^{R}	This study
pT2S300	Derivative of pAGM8031 encoding XpsE, XpsF and XpsG, H, I, J, K, XpsL$_{D147stop}$, M, C and XpsD$_{\Delta 103-523}$ under control of the native promoters and encoding 4×c-Myc-XpsL under control of the native promoter and containing the *xpsL* Shine Dalgarno sequence outside the *xps* gene cluster, assembled from pT2S290, pT2S032, pT2S034, pT2S190, pICH54055, pICH50914 and pAGM8031; Sm^{R}	This study
pT2S322	Derivative of pAGM8031 encoding XpsE, and XpsG, H, I, J, K, XpsL$_{D147stop}$, M, C and D under control of native promoters and encoding 4×c-Myc-XpsL under control of the native promoter and containing the *xpsL* Shine Dalgarno sequence outside the *xps* gene cluster, pT2S290, pT2S032, pICH54033 pT2S192, pICH54055, pICH50914 and pAGM8031; Sm^{R}	This study
pT2S306	Derivative of pAGM8031 encoding XpsE, XpsF and XpsG, H, I, J, K, L, M, C$_{\Delta 73-162}$, D under control of the native promoters and encoding XpsC-4×c-Myc downstream of the native promoter outside the *xps* gene cluster, assembled with pT2S301, pT2S032, pT2S034, pT2S054, pICH54055, pICH50914 and pAGM803; Sm^{R}	This study
pT2S319	Derivative of pAGM8031 encoding XpsC-4×c-Myc under control of the native promoter without other *xps* genes, assembled with pT2S301, pICH50872 and pAGM8031; Sm^{R}	This study
pT2S307	Derivative of pAGM8031 encoding XpsE, XpsF and XpsG, H, I, J, K, XpsL$_{D147stop}$, M, C$_{\Delta 73-162}$ and D under control of the native promoters and encoding XpsC-4×c-Myc under control of the native promoter outside the *xps* gene cluster, assembled with pT2S301, pT2S032, pT2S034, pT2S194, pICH54055, pICH50914 and pAGM8031; Sm^{R}	This study
pT2S317	Derivative of pAGM8031 encoding XpsE, XpsF and XpsG, H, I, J, K, L, M$_{\Delta 34-178}$, C$_{\Delta 73-162}$ and D under control of the native promoters and encoding XpsC-4×c-Myc under control of the native promoter outside the *xps* gene cluster, assembled with pT2S301, pT2S032, pT2S034, pT2S189, pICH54055, pICH50914 and pAGM8031; Sm^{R}	This study
pT2S333	Derivative of pAGM8031 encoding XpsE, XpsF and XpsG, H, I, J, K, L, M, C$_{\Delta 73-162}$ and XpsD$_{\Delta 103-523}$ under control of the native promoters and encoding XpsC-4×c-Myc under control of the native promoter outside the *xps* gene cluster, assembled with pT2S301, pT2S032, pT2S034, pT2S172, pICH54055, pICH50914 and pAGM8031; Sm^{R}	This study
[1] Ap, ampicillin; Gm, gentamycin; Km, kanamycin; r, resistant; Sm, spectinomycin		

References

Canteros, B.I. (1990). Diversity of plasmids and plasmid-encoded phenotypic traits in *Xanthomonas campestris* pv. *vesicatoria*. PhD thesis. University of Florida.

Drehkopf, S., Otten, C., and Büttner, D. (2022). Recognition of a translocation motif in the regulator HpaA from *Xanthomonas euvesicatoria* is controlled by the type III secretion chaperone HpaB. Front Plant Sci 13, 955776.

Drehkopf, S., Otten, C., Hausner, J., Seifert, T., and Büttner, D. (2020). HrpB7 from *Xanthomonas campestris* pv. *vesicatoria* is an essential component of the type III secretion system and shares features of HrpO/FliJ/YscO family members. Cell Microbiol 22, e13160.

Engler, C., Youles, M., Gruetzner, R., Ehnert, T.M., Werner, S., Jones, J.D., Patron, N.J., and Marillonnet, S. (2014). A golden gate modular cloning toolbox for plants. ACS Synth Biol 3, 839-843.

Escolar, L., Van den Ackerveken, G., Pieplow, S., Rossier, O., and Bonas, U. (2001). Type III secretion and *in planta* recognition of the *Xanthomonas* avirulence proteins AvrBs1 and AvrBsT. Mol Plant Pathol 2, 287-296.

Figurski, D., and Helinski, D.R. (1979). Replication of an origin-containing derivative of plasmid RK2 dependent on a plasmid function provided *in trans*. Proc Natl Acad Sci USA 76, 1648-1652.

Hausner, J., Jordan, M., Otten, C., Marillonnet, S., and Büttner, D. (2019). Modular cloning of the type III secretion gene cluster from the plant-pathogenic bacterium *Xanthomonas euvesicatoria*. ACS Synth Biol 8, 532-547.

Karimova, G., Dautin, N., and Ladant, D. (2005). Interaction network among *Escherichia coli* membrane proteins involved in cell division as revealed by bacterial two-hybrid analysis. J Bacteriol 187, 2233-2243.

Kousik, C.S., and Ritchie, D.F. (1998). Response of bell pepper cultivars to bacterial spot pathogen races that individually overcome major resistance genes. Plant Disease 82, 181-186.

Ménard, R., Sansonetti, P.J., and Parsot, C. (1993). Nonpolar mutagenesis of the *ipa* genes defines IpaB, IpaC, and IpaD as effectors of *Shigella flexneri* entry into epithelial cells. J Bacteriol 175, 5899-5906.

Morbitzer, R., Elsässer, J., Hausner, J., and Lahaye, T. (2011). Assembly of custom TALE-type DNA binding domains by modular cloning. Nucleic Acids Res 39, 5790-5799.

Otten, C., and Büttner, D. (2021). HrpB4 from *Xanthomonas campestris* pv. *vesicatoria* acts similarly to SctK proteins and promotes the docking of the predicted sorting platform to the type III secretion system. Cell Microbiol 23, e13327.

Otten, C., Seifert, T., Hausner, J., and Büttner, D. (2021). The contribution of the predicted sorting platform component HrcQ to type III secretion in *Xanthomonas campestris* pv. *vesicatoria* depends on an internal translation start site. Front Microbiol 12, 752733.

Schulze, S., Kay, S., Büttner, D., Egler, M., Eschen-Lippold, L., Hause, G., Krüger, A., Lee, J., Müller, O., Scheel, D., Szczesny, R., Thieme, F., and Bonas, U. (2012). Analyses of new type III effectors from *Xanthomonas* uncover XopB and XopS as suppressors of plant immunity. New Phytol 195, 894-911.

Szczesny, R., Jordan, M., Schramm, C., Schulz, S., Cogez, V., Bonas, U., and Büttner, D. (2010). Functional characterization of the Xps and Xcs type II secretion systems from the plant pathogenic bacterium *Xanthomonas campestris* pv. *vesicatoria*. New Phytol 187, 983-1002.

Weber, E., Grützner, R., Werner, S., Engler, C., and Marillonnet, S. (2011). Assembly of designer TAL effectors by Golden Gate cloning. PLoS ONE 6, e19722.

Yanisch-Perron, C., Vieira, J., and Messing, J. (1985). Improved M13 phage cloning vectors and host strains: nucleotide sequences of the M13mp18 and pUC19 vectors. Gene 33, 103-119.
