# Supplementary material for: Modularization of the type II secretion gene cluster from Xanthomonas euvesicatoria facilitates the identification of a structurally conserved XpsCLM assembly platform complex
Source: PLoS Pathog. 2025 Apr 9;21(4):e1013008. doi: 10.1371/journal.ppat.1013008 (PMC11981180; doi:10.1371/journal.ppat.1013008)
Supplement: S2 Table — (PDF) [file ppat.1013008.s002.pdf]

Table S2: Primers used in this study:

**Name[1]**	**Sequence (5‘-3‘) [2]**
**Deletion of the *xps* gene cluster**	
Δ*xps*-5'-fw	TTT **GGTCTC** T *CGAC* GCTGAGCATTTCGTCCAGAT
Δ*xps*-5'-rv	TTT **GGTCTC** T *ATGC* AGCCACTTGAGAAGGGT
Δ*xps*-3'-fw	TTT **GGTCTC** T *GCAT* CGATGCTCGATTGGATCGAC
Δ*xps*-3'-rv	TTT **GGTCTC** T *ATGG* CTCCGGTAGCTATACCTATA
**Modularization of the *xps* gene cluster**	
Amplification of *xpsE* including the native promoter	
Promoter 1 nt -365 fw	TTT **GGTCTC** A *ACAT* GGAGGCTTCGAGGCCAAGGCCGGC
Promoter 1 nt +4 rv	TTT **GGTCTC** A *TCAC* ACTCGTCTCCACTGGT
xpsE nt 1 fw	TTT **GGTCTC** A *GTGA* ACGCGGTTGCCGTCGA
xpsE nt 936 rv	TTT **GGTCTC** A *CTCG* GTAAAGCCCAGCTTGTA
xpsE nt 932 fw	TTT **GGTCTC** A *CGAG* GACTTCTTGCCGCAGTTCCG
xpsE nt 1069 rv	TTT **GGTCTC** A *ATCT* TCGACGGTGATGATTTT
xpsE nt 1065 fw	TTT **GGTCTC** A *AGAT* CCGGTGGAATACCAGATCGA
xpsE nt 1728 rv	TTT **GGTCTC** A *ACAA* AGCGTCACGCATCCTCCGTGACGC
Amplification of *xpsF* including the native promoter	
Promoter 2 nt -175 fw	TTT **GGTCTC** A *ACAT* GGAGTCGGTAAGGCAGCAGAAACA
Promoter 2 nt +4 rv	TTT **GGTCTC** A *GCAT* CAGGCTGGGAGGCCGG
xpsF nt 1 fw	TTT **GGTCTC** A *ATGC* CCCTCTACCGTTACAA
xpsF nt 266 rv	TTT **GGTCTC** A *CTCG* GGCAGATCCATCAGAAT
xpsF nt 270 fw	TTT **GGTCTC** A *CGAG* GACGAAAAAAGCCGGCGGGT
xpsF nt 1218 rv	TTT **GGTCTC** A *ACAA* AGCGTCAGCCGATTGCATTGGTGA
Amplification of *xpsG - xpsD* including the native promoter	
Promoter 3 nt -209 fw	TTT **GAAGAC** AA *CTCA* ACATGGAGGAGCGGAGCTCTGCGCAACGGTGCACTCTTCAC
Promoter 3 nt +4 rv	TTT **GAAGAC** AA *CTCG* GCATTGCAAAGGTTCCCGCA
xpsG nt 1 fw	TTT **GAAGAC** AA *CTCA* ATGCCCGCCGTGTCATGTGT
xpsG nt 492 rv	TTT **GAAGAC** AA *CTCG* TTATTGGTACTTGATGTCCG
xpsH nt -13 fw	TTT **GAAGAC** AA *CTCA* ATAAGCGCTCGCCATGCGCG
xpsH nt 510 rv	TTT **GAAGAC** AA *CTCG* TCATGGCGCCGGCGTCCGCA
xpsI nt 1 fw	TTT **GAAGAC** AA *CTCA* ATGAAGCGTCAGCGCGGTTA
xpsI nt 417 rv	TTT **GAAGAC** AA *CTCG* ACAACTCATGGCGGGCTGCCCTGC
xpsJ nt 1 fw	TTT **GAAGAC** AA *CTCA* ACATTGAGCCGTTCGCGCAGCGCA
xpsJ nt 429 rv	TTT **GAAGAC** AA *CTCG* TTCCGGCGGCAGCGGGCTGCT
xpsJ nt 426 fw	TTT **GAAGAC** AA *CTCA* GGAAACCCTTGCCCAAGGCGTGCA
xpsJ nt 636 rv	TTT **GAAGAC** AA *CTCG* TCATTGCCCTGCCCCCGGGT
xpsK nt 1 fw	TT **GAAGAC** AA *CTCA* ATGAGTCGTTCGCGCGGTGC
xpsK nt 852 rv	TTT **GAAGAC** AA *CTCG* GTCATTGCACTGCTGCTCCC
xpsL nt 1 fw	TTT **GAAGAC** AA *CTCA* TGACCGCATGGCGGGACACC
xpsL nt 449 rv	TTT **GAAGAC** AA *CTCG* CTCGCGCACATCGAGCACGCG
xpsL nt 446 fw	TTT **GAAGAC** AA *CTCA* CGAGGACGGCCAGCTCGATGCCGA
xpsL nt 1122 rv	AAA **GAAGAC** AA *CTCG* ACAATTAACGCTGCGTGGCATCGG
xpsM nt14 fw	TTT **GAAGAC** AA *CTCA* ACATTTAAGCGCGATCGCTGGATC
xpsM nt 654 rv	TTT **GAAGAC** AA *CTCG* TCAAGGCGCATTGCTGGCCT

Name[1]	Sequence (5‘-3‘) [2]
xpsC nt 8 fw	TTT **GAAGAC** AA *CTCA* TTGACATGATCGGCCTGCGC
xpsC nt 227 rv	TTT **GAAGAC** AA *CTCG* CTCGGCAAACGCCGGGTGCGC
xpsC nt 223 fw	TTT **GAAGAC** AA *CTCA* CGAGGACCGCCTGCCGCATCCGTT
xpsC nt 795 rv	TTT **GAAGAC** AA *CTCG* TCATTGGGTCTGACCGGGGG
xpsC tagN fw	TT **GGTCTC** A *ACAT* CCTGATGCGCCTTGACATGATCGG
xpsC tagN rv	TT **GGTCTC** A *ACAA* AAGCTCATTGGGTCTGACCGGGGG
xpsD nt 1 fw	TTT **GAAGAC** AA *CTCA* ATGAGTGAACGCATGACGCC
xpsD nt 1572 rv	TTT **GAAGAC** AA *CTCG* GCCAGCACCGCCCGTACCATC
xpsD nt 1568 fw	TTT **GAAGAC** AA *CTCA* TGGCCTCCCATCAGCTGCTGGTAG
xpsD nt 2292 rv	TTT **GAAGAC** AA *CTCG* ACAAAGCGCTATCTACCCTTCTCAAGTG
**Deletion constructs of individual *xps* genes**	
dxpsL1 rv	AAA **GAAGAC** AA *CTCG* ACAAGGCCAGCAGCGATTGTTGCC
dxpsL2 fw	TTT **GAAGAC** AA *CTCA* ACATGGCCGAACGGCAGCAATTGATG
dxpsM1 rv	AAA **GAAGAC** AA *CTCG* ACAAGGGTGAACCATGGGTGCACCAG
dxpsM2 fw	TTT **GAAGAC** AA *CTCA* ACATACCCTGGATATCGCCTTCGAGCTG
dxpsL 147stop rv	TTT **GAAGAC** AA *CTCG* CTCGCGCACATCAGAGCACGCG
dxpsC nt 485 fw	TTT **GAAGAC** AA *CTCA* CGAGGGGCGGGCAGCCGCCCACCG
dxpsD rv	TTT **GAAGAC** AA *CTCG* GCCAGCGAAATTGAAGGTGGCACTG
**Expression constructs for complementation studies**	
xpsE-FLAG rv	T **GGTCTC** A *ACAA* AGCGTC ACTTATCGTCGTCATCTTTGTAATCCGCATCCTCCGTGACGC
P3 comp fw	TTT **GGTCTC** A *GGAG* GAGCGGAGCTCTGCGCAACG
P3 comp rv	TTT **GGTCTC** A *CATT* TGCAAAGGTTCCCGCATGGG
P3 mut fw	TT **GAAGAC** TT *GGAG* CGCTTTACGAATTCCCATGGGGAG
P3 mut rv	TT **GAAGAC** TT *CATT* AACGATTTCCTTGCACGTCAATG
xpsL comp fw	TTT **GAAGAC** AA *CTCA* AATGACCGCATGGCGGGACAC
xpsL comp rv	TTT **GAAGAC** AA *CTCG* AGCGTTAACGCTGCGTGGCATCGGC
xpsL+SD comp fw	TT **GGTCTC** A *TATG* GGAAGAGGGAGCAGCAGTGC
xpsM comp fw	TTT **GAAGAC** AA *CTCA* AATGCCACGCAGCGTTAAGCGC
xpsM comp rv	TTT **GAAGAC** AA *CTCG* AGCGTCAAGGCGCATTGCTGGCCTC
P3 compC fw	TT **GGTCTC** A *ACAT* GGAGGAGCGGAGCTCTGCGCAACG
P3 compC rv	TT **GGTCTC** A *ACAA* GCATTGCAAAGGTTCCCGCATGGG
xpsC comp fw	TT **GGTCTC** A *ACAT* ATGCGCCTTGACATGATCGG
xpsC comp rv	TT **GGTCTC** A *ACAA* AGCGTCATTGGGTCTGACCGGGGG
xpsD comp fw	TTT **GAAGAC** AA *CTCA* AATGAGTGAACGCATGACGCC
xpsD comp rv	TTT **GAAGAC** AA *CTCG* AGCG CTATCTACCCTTCTCAAGTGG
xpsD BsaI fw	TTT **GGTCTC** T *TATG* ACGCCGCGCCTGTTTCCCGTGTC
xpsD BsaI rv	TTT **GGTCTC** T *CACC* TCTACCCTTCTCAAGTGGCTGC
xpsD 528 fw	TTT **GGTCTC** T *GCTG* CTGGTAGAGGTATTTG
xpsD 528 rv	TTT **GGTCTC** T *CAGC* TGATGGGAGTCCAGCAC
**Generation of fluorescence- and epitope-tagged constructs**	
4xMyc-N fw	TTT **GAAGAC** AA *AATG* GAACAAAAGTTGATCTCTGAAGAGG
4xMyc-N rv	TTT **GAAGAC** AA *CAGG* TTAAGGTCCTCTTCAGAAATAAGTTTTTG
4xMyc-C fw	TTT **GAAGAC** AA *GCTA* AGGAACAAAAGTTGATCTCTGAAGAGGAC
4xMyc-C rv	TTT **GAAGAC** AA *AAGC* TCAAAGGTCCTCTTCAGAAATAAG

Name[1]	Sequence (5‘-3‘) [2]
xpsL tagN fw	TT **GGTCTC** A *ACAT* CCTGATGACCGCATGGCGGGACAC
xpsL tagN rv	TT **GGTCTC** A *ACAA* AAGCTTAACGCTGCGTGGCATCGG
P3xpsLSD rv	TTT **GGTCTC** A *CATT* GCACTGCTGCTCCCTCTCC
xpsD tagC fw	TT **GGTCTC** A *ACAT* AATGAGTGAACGCATGACGCC
xpsD_tagC rv	TT **GGTCTC** A *ACAA* TAGCTCTACCCTTCTCAAGTGGCT
xpsC tagC fw	TT **GGTCTC** A *ACAT* AATGATGCGCCTTGACATGATCGG
xpsC tagC rv	TT **GGTCTC** A *ACAA* TAGCTTGGGTCTGACCGGGGGTAG
**Expression constructs for BACTH and pull-down assays**	
xpsL BACTH fw	TT **GGTCTC** A *TATG* ACCGCATGGCGGGACACC
xpsL BACTH rv	TT **GGTCTC** G *CACC* ACGCTGCGTGGCATCGGCC
xpsF BACTH fw	TT **GGTCTC** A *TATG* CCCCTCTACCGTTACAAGG
xpsF BACTH rv	TT **GGTCTC** G *CACC* GCCGATTGCATTGGTGAGGTC
xpsM BACTH fw	T **GAAGAC** AA *CTCA* TATGCCACGCAGCGTTAAGCGCG
xpsM BACTH rv	T **GAAGAC** AA *CTCG* CACCAGGCGCATTGCTGGCCTCCG
xpsC BACTH fw	TTT **GGTCTC** T *TATG* CGCCTTGACATGATC
xpsC BACTH rv	TTT **GGTCTC** T *CACC* TTGGGTCTGACCGGGGGTAG
xpsE BACTH fw	TTT **GGTCTC** T *TATG* GTGAACGCGGTTGCCGTC
xpsE BACTH rv	TTT **GGTCTC** T *CACC* CGCATCCTCCGTGAC

[1] fw, forward; rv, reverse.
[2] *Bpi*I and *Bsa*I sites are written in bold, overhangs are shown in italics.
