# Supplementary material for: Modularization of the type II secretion gene cluster from Xanthomonas euvesicatoria facilitates the identification of a structurally conserved XpsCLM assembly platform complex
Source: PLoS Pathog. 2025 Apr 9;21(4):e1013008. doi: 10.1371/journal.ppat.1013008 (PMC11981180; doi:10.1371/journal.ppat.1013008)
Supplement: S1 Appendix — (PDF) [file ppat.1013008.s003.pdf]

Appendix S1

Golden Gate assembly of modular T2S gene cluster constructs

Deletion of *xps* genes in the modular T2S gene cluster

To delete *xpsE* in the modular *xps*-T2S gene cluster, the level M construct was assembled as described above replacing level 1 construct pT2S032 (containing *xpsE* and its native promoter) by the dummy module pICH54022, thus resulting in level M construct pT2S069. Similarly, level 1 construct pT2S038 (containing *xpsF* and its native promoter) was replaced by the dummy module pICH54033 to delete *xpsF*, leading to level M construct pT2S085. For the introduction of an in-frame deletion of codons 33 - 258 in *xpsL*, flanking regions were amplified by PCR using primer pairs xpsK nt 1 fw / dxpsL1 rv and dxpsL2 fw / xpsM nt 654 rv (Table S2). The resulting amplicons were inserted into vector pAGM9121 using *Bpi*I and T4 ligase, leading to constructs pT2S096 and pT2S091. The corresponding modules were assembled with modules containing *xpsJ* (pT2S016 and pT2S017) as well as *xpsM, xpsC* and *xpsD* (pT2S023, pT2S024 and pT2S026) in the level -1 vector pAGM1311 using *Bsa*I and T4 ligase to generate pT2S103 and pT2S099, respectively. Both modules were assembled with pT2S015 (*xpsG, xpsH* and *xpsI* downstream of the native promoter) in vector pAGM41331 using *Bpi*I and T4 ligase to generate the level 0 construct pT2S107 which contains the *xpsG-xpsD* operon with a deletion of codons 33-258 in *xpsL*. The corresponding module was transferred into the level 1 vector pICH47761 using *Bsa*I and T4 ligase to generate pT2S126. Assembly with additional level 1 modules led to the generation of the final level M construct pT2S156, which encodes the complete *xps* gene cluster with a deletion in *xpsL*.

To introduce a nonsense mutation at codon 147 of *xpsL* (GAT → TGA), the 5' region of *xpsL* was amplified by PCR using primers xpsL part 1 fw / dxpsL 147stop rv and the resulting amplicon was inserted into pAGM9121 using *Bpi*I and T4 ligase, leading to the level -2 construct pT2S174. The corresponding module containing the first 449 nucleotides of *xpsL* with a stop codon was assembled with modules containing *xpsJ, xpsK* and the 3' region of *xpsL* in the level -1 vector pAGM1311 and subsequently transferred into the level 0 vector pAGM41331, thus generating construct pT2S187 which contains the *xpsG - xpsD* operon. The corresponding module was inserted into the level 1 vector pICH47761 to generate construct pT2S192 and used to assemble the final *xps* gene cluster in the level M vector pAGM8031 with a stop codon in *xpsL* (construct pT2S200).

To introduce a deletion into *xpsM*, flanking regions were amplified with primers xpsL nt 446 fw / dxpsM1 rv and dxpsM2 fw / xpsC nt 795 rv and the resulting amplicons were cloned into vector pAGM9121 using *Bpi*I and T4 ligase, thus leading to constructs pT2S097 and pT2S092. The corresponding modules were assembled with modules containing *xpsJ, xpsK*, *xpsL* and *xpsM* in the level -1 vector pAGM1311 using *Bsa*I and T4 ligase to generate pT2S104 and pT2S100. Both constructs were used to assemble the *xpsG - xpsD* operon with a deletion of

codons 34 - 178 in *xpsM* in vector pAGM41331, resulting in the level 0 construct pT2S108. The operon was subsequently transferred into the level 1 vector pICH47761 to generate construct pT2S127 and subsequently inserted into the final level M vector, leading to construct pT2S138 which contains the complete *xps* gene cluster with a deletion in *xpsM*.

To introduce a deletion into *xpsC*, the 3' region was amplified by PCR using primers dxpsC fw / xpsC part 2 rv and the amplicon was cloned into vector pAGM9121 using *Bpi*I and T4 ligase. The resulting construct pT2S051 was used to assemble the level -1 module pT2S052, which contains *xpsM, xpsC* with a deletion of codons 77-162 and *xpsD*, and was generated by Golden Gate assembly of modules from constructs pT2S023 (codons 1 – 76 of *xpsC*), pT2S051 (codons 163 - 265 of *xpsC*), pT2S025 (5' region of *xpsD*) and pT2S026 (3' region of *xpsD*). Construct pT2S052 was used to assemble the *xpsG* – *xpsD* operon containing the deletion in *xpsC* in the level 0 vector pICH41331 (construct pT2S053). The corresponding module was subsequently cloned into the level 1 vector pICH47761 using *Bsa*I and T4 ligase to generate construct pT2S054. The modular *xps* gene cluster with the mutation in *xpsC* was generated as described above and shown in Fig. 1.

To introduce a deletion into *xpsD*, a corresponding gene fragment lacking codon 103 - 523 was amplified by PCR using primers xpsD nt 1 fw / dxpsD rv and the amplicon was inserted into vector pAGM9121 using *Bpi*I and T4 ligase, resulting in construct pT2S056. The corresponding module was assembled with modules from constructs pT2S023 (5' region of *xpsC*), pT2S024 (3' region of *xpsC*) and pT2S026 (3' region of *xpsD*) in the level -1 vector pAGM1311 using *Bsa*I and T4 ligase (construct pT2S057) and subsequently combined with modules from constructs pT2S015 and pT2S021 in the level 0 vector pAGM41331, thus generating construct pT2S058 which contains the *xpsG* - *xpsD* operon with a deletion in *xpsD.* The operon was transferred to the level 1 vector pICH47761, resulting in construct pT2S059, and used to assemble the final level M construct pT2S060 with a deletion in *xpsD* (construct pT2S060).

Generation of expression cassettes for complementation studies

For complementation of the *xpsE* mutant phenotype, *xpsE* and the corresponding promoter were amplified in four PCR reactions using primers Promoter 1 nt -355 fw / Promoter 1 nt -1 rv, xpsE nt 1 fw / xpsE nt 936 rv, xpsE nt 932 fw / xpsE nt 1069 rv and xpsE nt 1065 fw / xpsE-FLAG rv (Table S2). A C-terminal FLAG epitope-encoding sequence was introduced by primer xpsE-FLAG rv. The resulting PCR amplicons were cloned into vector pAGM1311 using *BsaI* and T4 ligase, thus generating pT2S003. The corresponding module was inserted into level 0 vector pICH41331, leading to construct pT2S004 and subsequently transferred into level 1 vector pICH47732. The resulting construct pT2S071 was used to replace construct pICH54011

containing the dummy module at position 1 of the level M construct pT2S077, which contains the deletion in *xpsE*.

For complementation of the *xpsF* mutant, *xpsF* and the native promoter in construct pT2S006 were inserted into the level 1 vector pICH47732 using *Bsa*I and T4 ligase. The resulting construct pT2S084 was used to replace construct pICH54011 (dummy module) at position 1 of the level M construct pT2S086 containing the deletion in *xpsF*.

To complement the *xpsL* deletion mutant phenotype, *xpsL* and the promoter of *xpsG* from construct pT2S038 were amplified by PCR using primers xpsL comp fw / xpsL comp rv and P3 comp fw / P3 comp rv and the amplicons were cloned into vector pAGM9121 using *BpiI* and T4 ligase to generate constructs pT2S119 (*xpsL*) and pT2S075 (promoter of *xpsG*). These modules were assembled in the level 1 vector pICH47732 to generate construct pT2S135. The resulting *xpsL* expression cassette was used to replace the dummy module at position 1 in the level M plasmid pT2S157 containing the deletion in *xpsL.* To introduce the *xpsL* expression cassette into the modular *xps* gene cluster containing the nonsense mutation in *xpsL*, the corresponding module from construct pT2S135 was inserted into level M constructs containing a stop codon in *xpsL*, resulting in construct pT2S201. For additional complementation studies, *xpsL* and 21 bp upstream region containing the putative Shine Dalgarno sequence were amplified by PCR using construct pT2S038 as template and primers xpsL+SD comp fw / xpsL comp rv. The resulting amplicon was cloned into vector pAGM9121 using *BpiI* and T4 ligase to generate construct pT2S203. The corresponding module was assembled with construct pT2S075 in the level 1 vector pICH47732, thus generating construct pT2S206. The resulting *xpsL* expression cassette was inserted into the level M constructs containing either an in-frame deletion or a stop codon, thus replacing the dummy module at position 1 and leading to constructs pT2S207 and pT2S208.

To complement the *xpsM* mutant phenotype, *xpsM* was amplified by PCR using primers xpsM comp fw / xpsM comp rv and the corresponding PCR product was cloned into the level 0 vector pAGM9121 using *BpiI* and T4 ligase to generate construct pT2S114. Constructs pT2S114 and pT2S075 were used to assemble the *xpsM* expression cassette in the level 1 vector pICH47732, resulting in construct pT2S125. The corresponding insert was inserted at position 1 in the level M construct pT2S158 which contains the modular T2S gene cluster with a deletion in *xpsM*.

For the complementation of the *xpsC* mutant phenotype, the native promoter upstream of *xpsG* and the *xpsC* gene were amplified by PCR using construct pT2S038 as template and primers P3 compC fw / P3 compC rv and xpsC comp fw / xpsC comp rv, respectively, and the resulting PCR products were cloned into vector pUC57 using *EcoRV* and T4 ligase, resulting in constructs pT2S044 and pT2S045. The corresponding modules were assembled in the level 0 vector pICH41331 and subsequently inserted into the level 1 vector pICH47732 using *Bsa*I

and ligase, resulting in construct pT2S049. The *xpsC* expression cassette was inserted into the final level M construct containing the modular *xps* gene cluster with the deletion in *xpsC.*
To complement the *xpsD* mutant phenotype, *xpsD* was amplified by PCR using construct pT2S038 as template and primers xpsD comp fw / xpsD comp rv. The resulting amplicon was cloned into vector pAGM9121 using *BpiI* and T4 ligase to generate construct pT2S209. The corresponding module was assembled with the native promoter upstream of *xpsG* in the level 1 vector pICH47732, resulting in construct pT2S225. The *xpsD* expression cassette was inserted at position 1 of the level M construct containing the modular T2S gene cluster with the deletion in *xpsD.*

Introduction of epitope- and mCherry-encoding sequences into the modular T2S gene cluster

To generate an XpsD-mCherry fusion, *xpsD* was amplified by PCR using construct pT2S038 as template and primers xpsD tagC fw / xpsD tagC rv. The resulting PCR product was cloned into vector pAGM1311 using *Bsa*I and T4 ligase, thus generating construct pT2S210. The *xpsD-mCherry* cassette was generated by assembly of the level -1 modules pT2S210 (*xpsD*), pAGB1000 (encodes a 2×AKLEGPAGL linker sequence) and pAGB1048 (encodes mCherry) in the level 0 vector pICH41308, resulting in construct pT2S219. The corresponding insert was assembled with the *xpsG* promoter (pT2S075) and a transcriptional terminator (pAGB232) in the level 1 vector pICH47732, thus generating construct pT2S236. The corresponding expression cassette encoding XpsD-2×AKLEGPAGL-mCherry under control of the *xpsG* promoter was inserted at position 1 of a modular *xps* gene cluster deleted in *xpsD*. The level M plasmid pT2S253 encoding XpsD-2×AKLEGPAGL-mCherry under control of the *xpsG* promoter without other *xps* genes was generated by assembly of constructs pT2S236, pICH50872 and pAGM8031 using *Bpi*I and T4 ligase.
To generate N- and C-terminal translational fusions of Xps proteins with a 4×c-Myc epitope, the 4×c-Myc-encoding sequence was amplified by PCR using construct pICSL50010 as template and primers 4xMyc-N fw / 4xMyc-N rv and 4xMyc-C fw / 4xMyc-C rv. The resulting PCR products were cloned into vector pICH41021 using *Sma*I and T4 ligase to generate constructs pAGB872 and pAGB873, respectively. For the generation of *xpsL* and *xpsC* expression constructs, both genes were amplified by PCR using primers xpsL tagN fw / xpsL tagN rv and xpsC tagC fw / xpsC tagC rv, respectively. The corresponding PCR products were cloned into vector pAGM1311 using *BsaI* and T4 ligase to generate pT2S205 and pT2S066, respectively. In addition, the promoter of the *xpsG* - *xpsD* operon and the predicted Shine Dalgarno sequence of *xpsL* were amplified by PCR using pT2S206 as template and primers P3 comp fw / P3xpsLSD rv. The PCR product was cloned into vector pUC57 using *EcoRV* and T4 ligase to generate construct pT2S285. *xpsL* (pT2S205) was assembled with the 4×c-Myc-encoding sequence in vector pICH41308, leading to construct pT2S289 (encodes 4×c-Myc-

XpsL). Similarly, *xpsC* (pT2S066) was assembled with the 4×c-Myc-encoding sequence in vector pICH41308, leading to construct pT2S293 (encodes XpsC-4×c-Myc). Both gene fusions were cloned downstream of the *xpsG* promoter and upstream of a transcriptional terminator in the level 1 vector pICH47732 using (a) level 0 constructs pT2S285 (*xpsG* promoter with predicted Shine Dalgarno sequence of *xpsL*), pT2S289 (encodes 4×c-Myc-XpsL), pAGB232 (transcriptional terminator) or (b) level 0 constructs pT2S293 (encodes XpsC-4×c-Myc), pT2S075 (*xpsG* promoter) and pAGB232 (transcriptional terminator). The resulting level 1 constructs pT2S290 (encodes 4×c-Myc-XpsL under control of the *xpsG* promoter with the predicted Shine-Dalgarno sequence of *xpsL*) and pT2S301 (encodes XpsC-4×c-Myc under control of the *xpsG* promoter) were used to insert both expression cassettes into level M constructs containing mutations in *xpsC/xpsD*, *xpsM/xpsC, xpsL/xpsC, xpsL/xpsD* or *xpsL/xpsM.* For this, level 1 constructs containing the *xpsG* - *xpsD* operon with corresponding mutations were introduced into the final level M construct as described above and assembled as follows: level 1 construct pT2S172 containing the *xpsG* - *xpsD* operon with deletions in *xpsC* and *xpsD* was generated by assembly of constructs pT2S022, pT2S023, pT2S051, pT2S056 and pT2S026 in vector pAGM1311, thus generating level -1 construct pT2S137 containing *xpsM* – *xpsD* with deletions in *xpsC* and *xpsD*. The corresponding module was assembled with pT2S015 and pT2S021 in level 0 vector pICH41331, leading to construct pT2S170 (contains the *xpsG* - *xpsD* operon with the native promoter and deletions in *xpsC* and *xpsD*), which was subsequently transferred to the level 1 vector pICH47761 using *BsaI* and T4 ligase to generate construct pT2S172.

For the assembly of the level 1 construct pT2S189 carrying the *xpsG* - *xpsD* operon with deletions in *xpsM* and *xpsC,* a PCR fragment was generated using construct pT2S052 as template and primers dxpsM2 fw / dxpsC1rv and cloned into vector pAGM9121 using *Bpi*I and ligase. The corresponding construct pT2S175 was assembled with pT2S025 and pT2S026 (containing *xpsD* fragments) in level -1 vector pAGM1311, thus generating construct pT2S179. The corresponding module was subsequently assembled with pT2S015 (*xpsG, xpsH* and *xpsI* including the *xpsG* promoter) and pT2S104 (*xpsJ, xpsK, xpsL* and codons 1 – 33 of *xpsM*) in the level 0 vector pICH41331, leading to construct pT2S180. The corresponding insert was transferred into level 1 vector pICH47761 to generate pT2S189 (contains the *xpsG* – *xpsD* operon with the native promoter and deletions in *xpsM* and *xpsC*).

For the generation of the level 1 construct pT2S194 carrying the *xpsG* - *xpsD* operon with a nonsense mutation in *xpsL* and an in-frame deletion in *xpsC*, constructs pT2S015 (contains *xpsG, xpsH* and *xpsI* and the *xpsG* promoter), pT2S178 (contains *xpsJ, xpsK* and *xpsL* with a nonsense mutation) and pT2S052 (contains *xpsM*, *xpsC* with a deletion of codons 73 – 162 and *xpsD*) were cloned into level 0 vector pICH41331, leading to construct pT2S181, which was subsequently transferred to the level 1 vector pICH47761 to generate construct pT2S194

(contains the *xpsG* – *xpsD* operon with the native promoter, a deletion in *xpsC* and a nonsense mutation in *xpsL*).

The level 1 construct pT2S190 carrying the *xpsG* - *xpsD* operon with a nonsense mutation in *xpsL* and an in-frame deletion in *xpsD* was assembled from constructs pT2S015 (*xpsG, xpsH* and *xpsI* including the *xpsG* promoter), pT2S178 (*xpsJ, xpsK* and *xpsL* with a nonsense mutation) and pT2S057 (*xpsM*, *xpsC* and *xpsD* with deletion of codons 103 - 523) in vector pICH41331, leading to construct pT2S182. The corresponding insert was subsequently cloned into level 1 vector pICH47761 using *BsaI* and T4 ligase to generate pT2S190.

The level 1 construct pT2S198 carrying the *xpsG* - *xpsD* operon with a nonsense mutation in *xpsL* and an in-frame deletion in *xpsM*, was assembled from constructs pT2S016, pT2S017, pT2S018, pT2S174 and pT2S097 in vector pAGM1311 to generate the level -1 construct pT2S188 which encodes XpsJ, XpsK, $XpsL_{D147stop}$ and amino acids 1-33 of XpsM. The corresponding module was assembled with pT2S015 (contains *xpsG, xpsH* and *xpsI* including the *xpsG* promoter) and pT2S100 (contains codons 179-217 of *xpsM* as well as *xpsC* and *xpsD*) in vector pICH41331, thus leading to construct pT2S193. The corresponding insert was cloned into vector pICH47761 to generate the level 1 construct pT2S198.

To insert a nonsense mutation upstream of *xpsC*, the *xpsG* promoter was amplified with primers P3 mut fw/ P3 mut rv and cloned into level 0 vector pICH41295 by blunt-end cloning. *xpsC* was amplified using primers xpsC tagN fw/xpsC tagN rv and the PCR product was cloned into level -1 vector pAGM1311, using *Sma*I and ligase. *xpsC* was assembled with the c-Myc epitope-encoding module in level 0 vector pICH41308 and subsequently cloned together with the *xpsG* promoter into level 1 vector pICH47732 and finally into the level M module containing the *xps* gene cluster with a deletion in *xpsC.*

<u>Introduction of epitope-encoding sequences into the modular T2S gene cluster for crosslinking studies</u>

For crosslinking studies, the expression cassette encoding 4×c-Myc-XpsL was inserted into level M constructs containing the modular *xps* gene cluster with a nonsense mutation in *xpsL* (pT2S296), a deletion of *xpsE* – *xpsD* (pT2S318), a nonsense mutation in *xpsL* and a deletion in *xpsM* (pT2S297), a nonsense mutation in *xpsL* and a deletion in *xpsC* (pT2S298), a nonsense mutation in *xpsL* and a deletion in *xpsE* (pT2S299), a nonsense mutation in *xpsL* and a deletion in *xpsD* (pT2S300) and a nonsense mutation in *xpsL* and a deletion in *xpsF* (pT2S322). Similarly, the expression cassette encoding XpsC-4×c-Myc fusion was inserted into level M constructs containing the modular *xps* gene cluster with a deletion in *xpsC* (pT2S306), a deletion of *xpsE* – *xpsD* (pT2S319), a deletion in *xpsC* and *xpsM* (pT2S317), a deletion in *xpsC* and *xpsL* (pT2S307) and a deletion in *xpsC* and *xpsD* (pT2S333). Level 1 constructs used to assemble the level M vectors for crosslinking studies are listed in Table S1.
