# Supplementary material for: Modularization of the type II secretion gene cluster from Xanthomonas euvesicatoria facilitates the identification of a structurally conserved XpsCLM assembly platform complex
Source: PLoS Pathog. 2025 Apr 9;21(4):e1013008. doi: 10.1371/journal.ppat.1013008 (PMC11981180; doi:10.1371/journal.ppat.1013008)
Supplement: S2 Appendix — (PDF) [file ppat.1013008.s004.pdf]

Appendix S2: Supplementary data sets

Measurements of halo sizes for protease activity assays

Strain	Halo width in mm				
	Replicates			Average	Standard deviation
	1	2	3		
**Figure 2**					
85-10	1	2	2	1.67	0.58
Δ*xps*	0	0	0	0	0
Δ*xps* + pT2S	3	4	4	3.67	0.58
Δ*xps* + pT2S	3	4	4	3.67	0.58
Δ*xps* + pT2SΔ*xpsD*	0	0	0	0	0
Δ*xps* + pT2SΔ*xpsD* + XpsD	3	4	4	3.67	0.58
Δ*xps* + pT2S	3	4	4	3.67	0.58
Δ*xps* + pT2SΔ*xpsE*	0	0	0	0	0
Δ*xps* + pT2SΔ*xpsE* + XpsE	3	4	4	3.67	0.58
**Figure 3**					
Δ*xps* + pT2S	4	4	3	3.67	0.58
Δ*xps* + pT2SΔ*xpsC*	0	0	0	0	0
Δ*xps* + pT2SΔ*xpsC* + XpsC	4	4	3	3.67	0.58
Δ*xps* + pT2SΔ*xpsM*	0	0	0	0	0
Δ*xps* + pT2SΔ*xpsM* + XpsM	4	4	3	3.67	0.58
Δ*xps* + pT2S	4	4	3	3.67	0.58
Δ*xps* + pT2S$xpsL_{\Delta 33\text{-}258}$	0	0	0	0	0
Δ*xps* + pT2S$xpsL_{\Delta 33\text{-}258}$+ XpsL	0	0	0	0	0
Δ*xps* + pT2S$xpsL_{\Delta 33\text{-}258}$ + $XpsL_{+SD}$	0	0	0	0	0
Δ*xps* + pT2S$xpsL_{D147stop}$	0	0	0	0	0
Δ*xps* + pT2S$xpsL_{D147stop}$ + XpsL	0	0	0	0	0
Δ*xps* + pT2S$xpsL_{D147stop}$ + $XpsL_{+SD}$	4	4	3	3.67	0.58
**Figure 6**					
Δ*xps* + pT2S	4	4	3	3.67	0.58
Δ*xps* + pT2S$xpsL_{D147stop}$ + c-Myc-XpsL	4	4	3	3.67	0.58
Δ*xps* + pT2S$xpsL_{D147stop}$ + c-Myc-XpsL/Δ*xpsE-D*	0	0	0	0	0
Δ*xps* + pT2S$xpsL_{D147stop}$ + c-Myc-XpsL/Δ*xpsC*	0	0	0	0	0
Δ*xps* + pT2S$xpsL_{D147stop}$ + c-Myc-XpsL/Δ*xpsM*	0	0	0	0	0
Δ*xps* + pT2S$xpsL_{D147stop}$ + c-Myc-XpsL/Δ*xpsE*	0	0	0	0	0
Δ*xps* + pT2S$xpsL_{D147stop}$ + c-Myc-XpsL/Δ*xpsF*	0	0	0	0	0
Δ*xps* + pT2S$xpsL_{D147stop}$ + c-Myc-XpsL/Δ*xpsD*	0	0	0	0	0
Δ*xps* + pT2S	4	3	4	3.67	0.58
Δ*xps* + pT2SΔ*xpsC* + XpsC-c-Myc	2	1	2	1.67	0.58
Δ*xps* + pT2SΔ*xpsC* + XpsC-c-Myc/Δ*xpsE-D*	0	0	0	0	0

Δ*xps* + pT2SΔ*xpsC* + XpsC-c-Myc/Δ*xpsM*	0	0	0	0	0
Δ*xps* + pT2SΔ*xpsC* + XpsC-c-Myc/*xpsL*$_{D147stop}$	0	0	0	0	0
Δ*xps* + pT2SΔ*xpsC* + XpsC-c-Myc/Δ*xpsD*	0	0	0	0	0
**Figure 7**					
Δ*xps* + pT2S	3	4	4	3.67	0.58
Δ*xps* + pT2SΔ*xpsD*	0	0	0	0	0
Δ*xps* + pT2SΔ*xpsD* + XpsD-mCherry	3	4	4	3.67	0.58
Δ*xps* + pT2SΔ*xps* + XpsD-mCherry	0	0	0	0	0
**Figure S8**					
Δ*xps* + pT2S	4	3	4	3.67	0.58
Δ*xps* + pT2SΔ*xpsC* + XpsC	4	3	4	3.67	0.58

Quantification of disease symptoms
Figure S2

	**Spots/ cm^2 leaf area**		
	**Strain**		
**Replicate**	**85-10**	**85-10Δ*xps***	**85-10Δ*xps* + pT2S**
1	13	1	11
2	14	3	16
3	21	4	14
4	11	5	15
5	23	9	25
6	20	5	20
7	17	10	19
average	17	5.29	17.14
Standard deviation	4.51	3.20	4.60

Number of fluorescent foci
Figure 7D

**Strain**	**Replicate**	**Number of cells with**			
		**0 foci**	**1 foci**	**2 foci**	**3 or more foci**
85-10Δ*xps* + pT2SΔ*xpsD* + XpsD-mCherry	1	0	124	324	197
	2	0	92	242	74
	3	0	85	319	119
85-10Δ*xps* + pT2SΔ*xps* + XpsD-mCherry	1	0	184	481	236
	2	0	49	138	45
	3	0	85	217	46
