# Supplementary material for: Modularization of the type II secretion gene cluster from Xanthomonas euvesicatoria facilitates the identification of a structurally conserved XpsCLM assembly platform complex
Source: PLoS Pathog. 2025 Apr 9;21(4):e1013008. doi: 10.1371/journal.ppat.1013008 (PMC11981180; doi:10.1371/journal.ppat.1013008)
Supplement: S1 Fig — The T2S system is composed of the assembly platform (shown in green; GspC, M, L and F and the hexameric ATPase GspE), the pseudopilus (blue; GspG, H, I J and K) and the OM secretin complex (yellow; GspD). Pseudopilins are processed by GspO (light grey), which is encoded outside the xps-T2S gene cluster of X. euvesicatoria. T2S substrates are exported into the periplasm via the general secretory (Sec) system or the twin-arginine translocation (Tat) system (dark grey) and subsequently recruited by the T2S system. The HR domain of GspC interacts with the secretin GspD. A second periplasmic domain of GspC is indicated as 2P domain. Letters refer to the nomenclature of Gsp proteins. IM, inner membrane; OM, outer membrane. (PDF) [file ppat.1013008.s005.pdf]

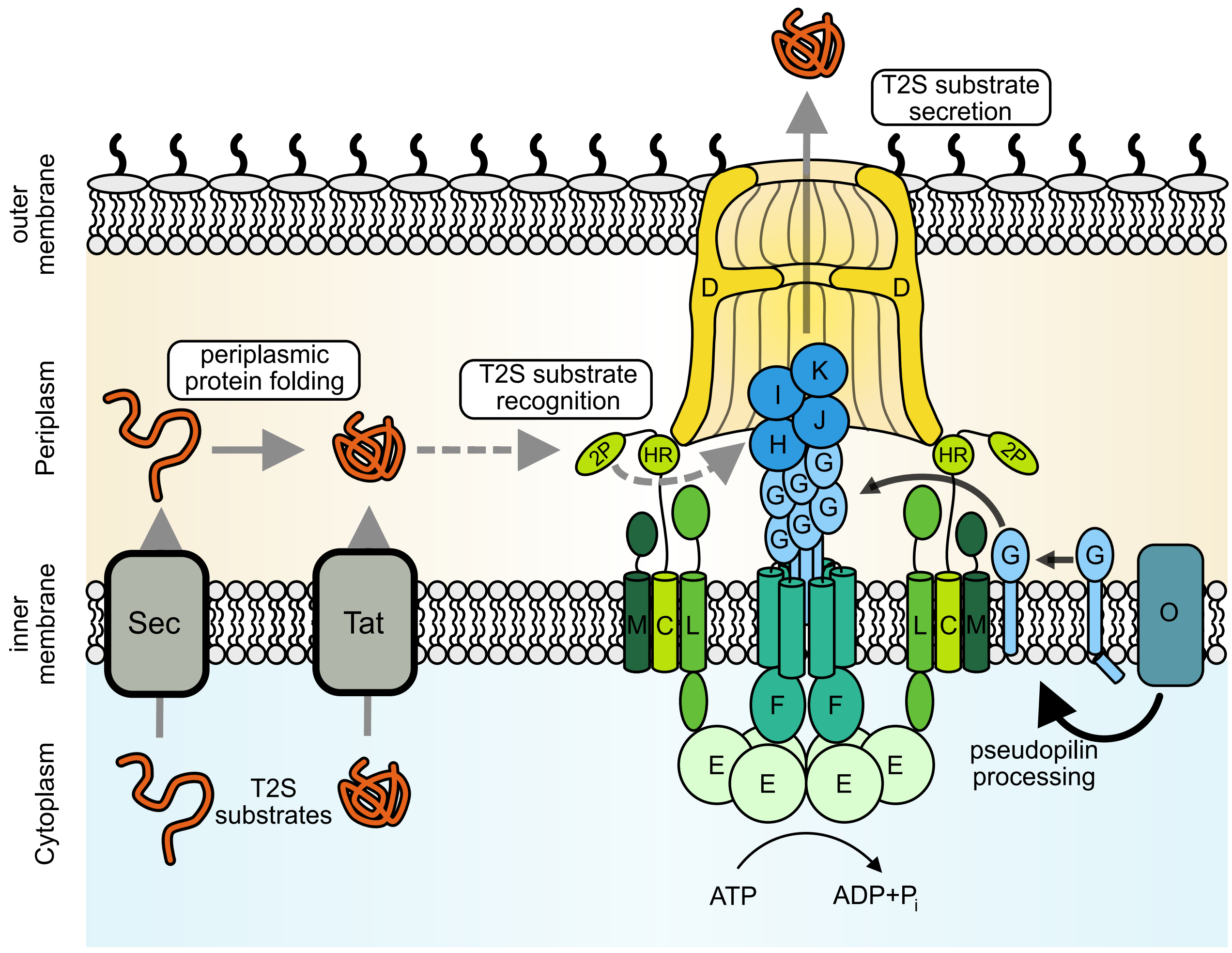

Supplemental figure 1

Goll *et al.*

Figure S1: Schematic representation of the T2S system.

The T2S system is composed of the assembly platform (shown in green; GspC, M, L and F and the hexameric ATPase GspE), the pseudopilus (blue; GspG, H, I J and K) and the OM secretin complex (yellow; GspD). Pseudopilins are processed by GspO (light grey), which is encoded outside the *xps*-T2S gene cluster of *X. euvesicatoria*. T2S substrates are exported into the periplasm via the general secretory (Sec) system or the twin-arginine translocation (Tat) system (dark grey) and subsequently recruited by the T2S system. The HR domain of GspC interacts with the secretin GspD. A second periplasmic domain of GspC is indicated as 2P domain. Letters refer to the nomenclature of Gsp proteins. IM, inner membrane; OM, outer membrane.
