# Supplementary material for: Modularization of the type II secretion gene cluster from Xanthomonas euvesicatoria facilitates the identification of a structurally conserved XpsCLM assembly platform complex
Source: PLoS Pathog. 2025 Apr 9;21(4):e1013008. doi: 10.1371/journal.ppat.1013008 (PMC11981180; doi:10.1371/journal.ppat.1013008)
Supplement: S2 Fig — (A) Leaves of susceptible ECW pepper plant were dip-infected with the wild-type strain 85-10 and the T2S deletion mutant 85-10∆xps (∆xps) with (+) or without (−) the modular xps-T2S expression construct (pT2S). Disease symptom formation was photographed seven weeks after infection. The experiment was performed three times with similar results. Spot formation on one representative leaf per strain is shown. (B) Quantitative analysis of spot formation. Spots were counted in seven different leaf areas per strain. Mean values of the number of spots per cm2 of leaf area and standard deviations are shown. Statistical significance was determined using an ANOVA with post-hoc HSD (Honestly Significant Difference) test (P<0.001). (PDF) [file ppat.1013008.s006.pdf]

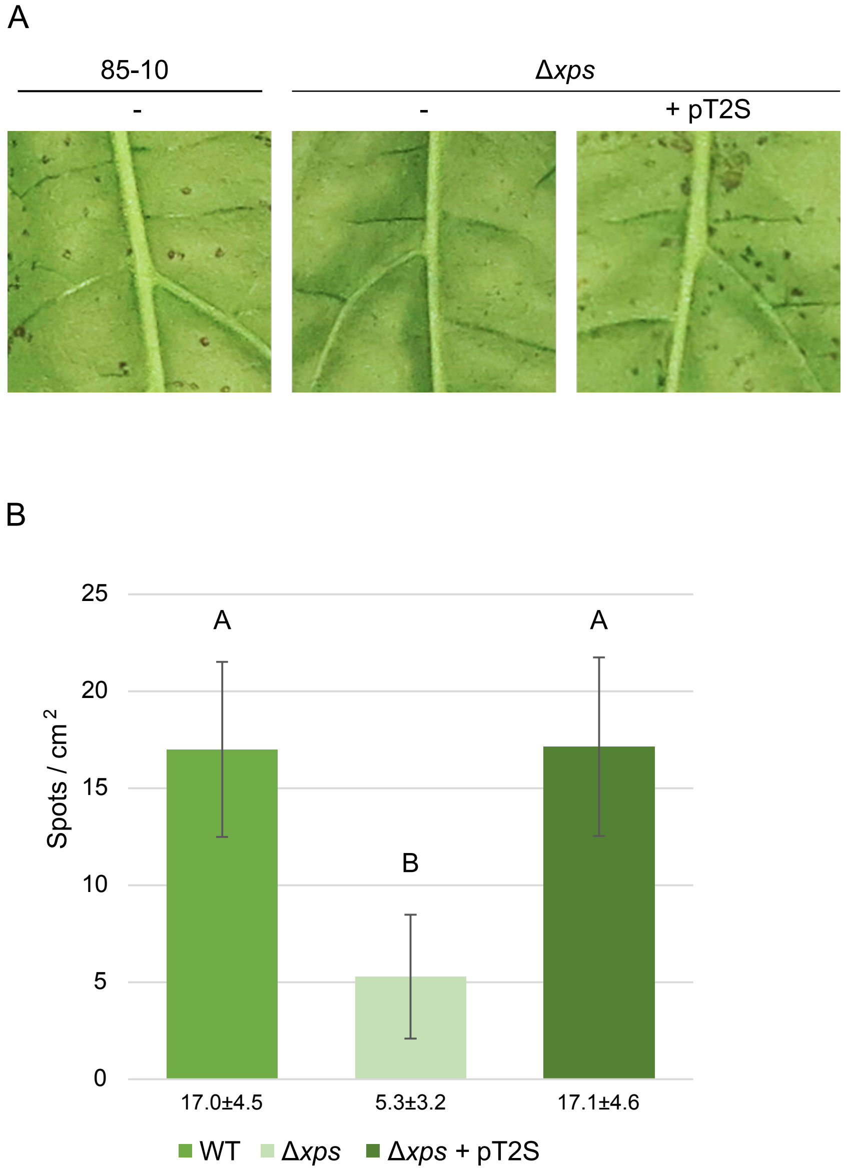

Supplemental figure 2

Goll *et al.*

Figure S2: The modular T2S system restores bacterial spot formation in an *xps*-T2S gene cluster mutant.

(A) Leaves of susceptible ECW pepper plant were dip-infected with the wild-type strain 85-10 and the T2S deletion mutant 85-10Δ*xps* (Δ*xps*) with (+) or without (-) the modular *xps*-T2S expression construct (pT2S). Disease symptom formation was photographed seven weeks after infection. The experiment was performed three times with similar results. Spot formation on one representative leaf per strain is shown.

(B) Quantitative analysis of spot formation. Spots were counted in seven different leaf areas per strain. Mean values of the number of spots per cm^2 of leaf area and standard deviations are shown. Statistical significance was determined using an ANOVA with post-hoc HSD (Honestly Significant Difference) test ($P<0.001$).
