# Supplementary material for: Modularization of the type II secretion gene cluster from Xanthomonas euvesicatoria facilitates the identification of a structurally conserved XpsCLM assembly platform complex
Source: PLoS Pathog. 2025 Apr 9;21(4):e1013008. doi: 10.1371/journal.ppat.1013008 (PMC11981180; doi:10.1371/journal.ppat.1013008)
Supplement: S3 Fig — Genes are represented by arrows, promoters by orange boxes. Deletions are indicated by black boxes. Letters refer to the nomenclature of xps genes. For complementation and localization studies, genes and gene fusions under control of the xpsG promoter were inserted upstream of xpsE into the modular T2S gene cluster constructs as indicated. (PDF) [file ppat.1013008.s007.pdf]

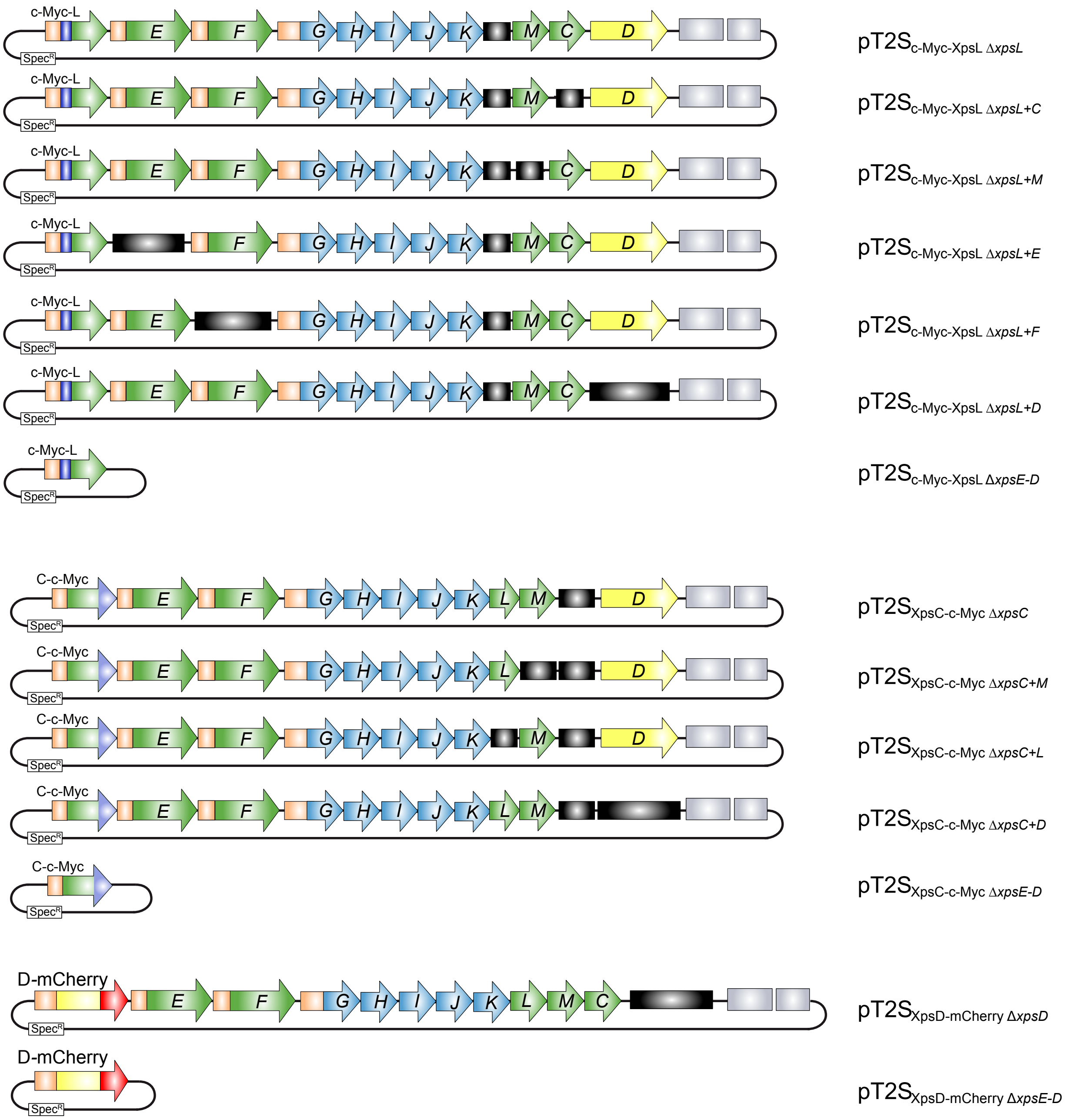

Supplemental figure 3

Goll *et al.*

Figure S3: Overview on modular T2S gene cluster constructs used in this study.

Genes are represented by arrows, promoters by orange boxes. Deletions are indicated by black boxes. Letters refer to the nomenclature of *xps* genes. For complementation and localization studies, genes and gene fusions under control of the *xpsG* promoter were inserted upstream of *xpsE* into the modular T2S gene cluster constructs as indicated.
