# Supplementary material for: Modularization of the type II secretion gene cluster from Xanthomonas euvesicatoria facilitates the identification of a structurally conserved XpsCLM assembly platform complex
Source: PLoS Pathog. 2025 Apr 9;21(4):e1013008. doi: 10.1371/journal.ppat.1013008 (PMC11981180; doi:10.1371/journal.ppat.1013008)
Supplement: S4 Fig — Secondary structures were predicted using the AlphaFold2 algorithm and the molecular visualization program UCSF ChimeraX [40,41,71]. N- and C-terminal regions are indicated in blue and red colours, respectively. The models on the right side show the per-residue model confidence score (pLDDT, predicted local distance difference test) which is scaled from 0 to 100 with blue colours indicating higher scores (70 – 100) and thus a higher confidence and more accurate prediction. (PDF) [file ppat.1013008.s008.pdf]

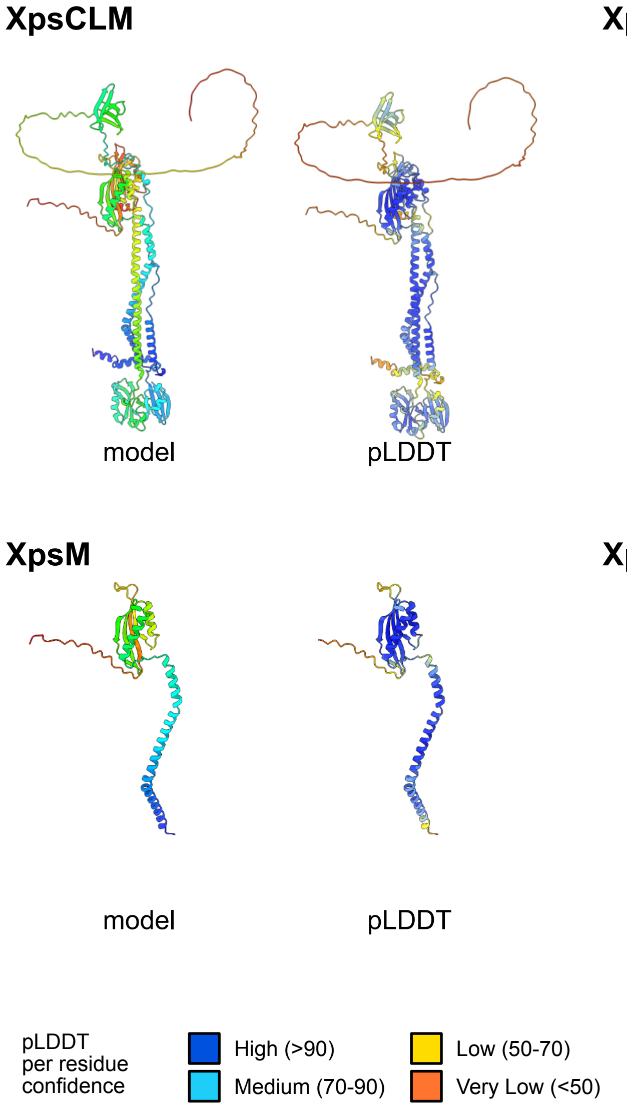

Supplemental figure 4

Goll *et al.*

Figure S4: Secondary structure predictions for XpsC, XpsL and XpsM from *X. euvesicatoria.* Secondary structures were predicted using the AlphaFold2 algorithm and the molecular visualization program UCSF ChimeraX [40, 41, 71|. N- and C-terminal regions are indicated in blue and red colours, respectively. The models on the right side show the per-residue model confidence score (pLDDT, predicted local distance difference test) which is scaled from 0 to 100 with blue colours indicating higher scores (70 – 100) and thus a higher confidence and more accurate prediction.
