# Supplementary material for: Modularization of the type II secretion gene cluster from Xanthomonas euvesicatoria facilitates the identification of a structurally conserved XpsCLM assembly platform complex
Source: PLoS Pathog. 2025 Apr 9;21(4):e1013008. doi: 10.1371/journal.ppat.1013008 (PMC11981180; doi:10.1371/journal.ppat.1013008)
Supplement: S5 Fig — (A) Predicted α-helices and β-sheets in XpsC from X. euvesicatoria. The amino acid sequence of XpsC (accession number CAJ25390) and the positions of predicted α helices and β sheets predicted by AlphaFold2 are shown [41,71]. Coloured rectangles refer to cytoplasmic, transmembrane and periplasmic regions as well as to the HR and 2P domains as indicated. Numbers indicate amino acid positions. (B) Predicted secondary structure elements in GspC proteins. α helices and β sheets were predicted and indicated as described in (A). The following proteins were analysed: XcpP from P. aeruginosa strain PAO1 (accession number CAA48581), OutC from D. dadantii strain 3937 (accession number CAA46369) and EpsC from V. cholerae strain N16961 (accession number P45777). (PDF) [file ppat.1013008.s009.pdf]

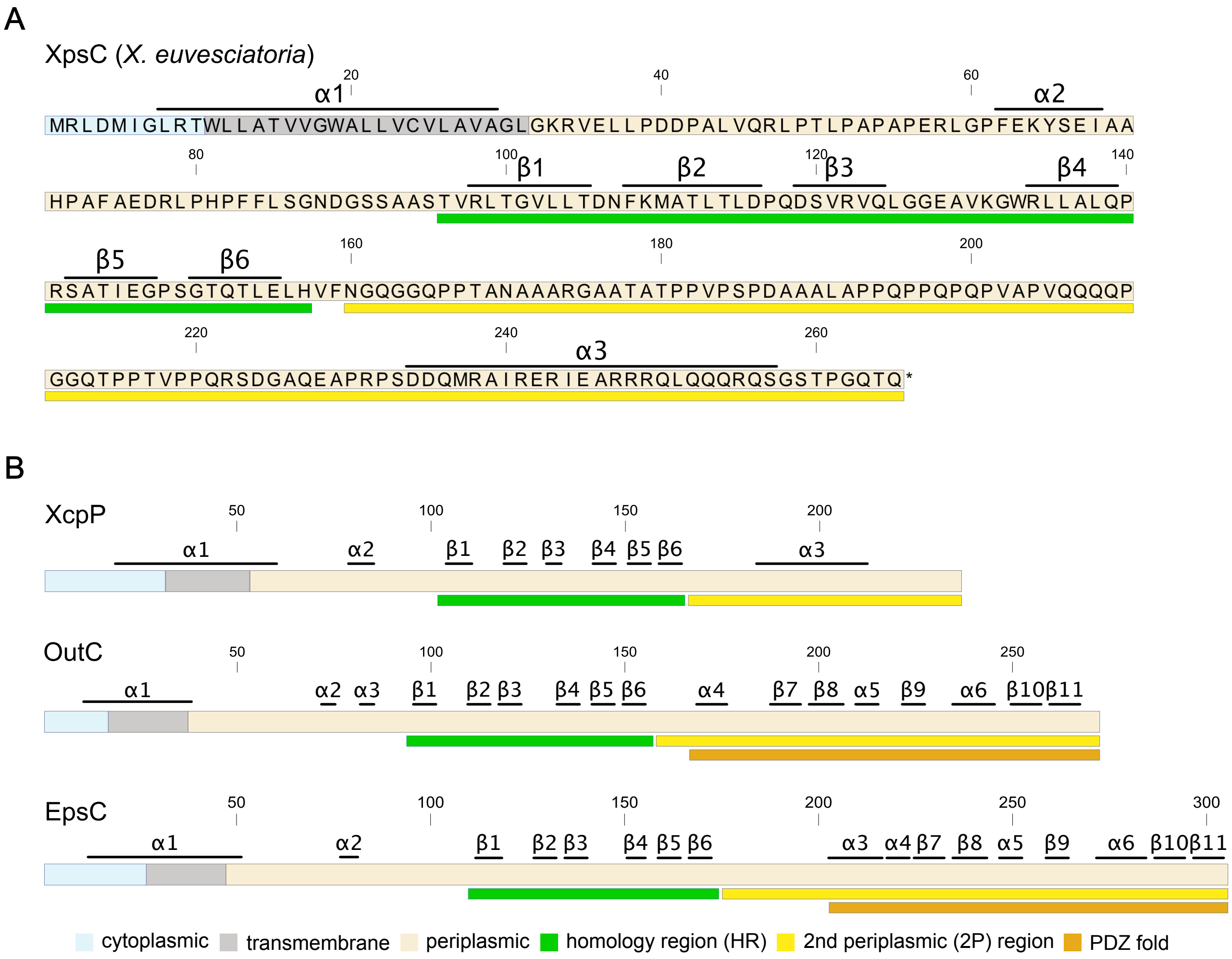

Supplemental figure 5

Goll *et al.*

Figure S5: Predicted secondary structures in XpsC and corresponding GspC proteins.

(A) Predicted α-helices and β-sheets in XpsC from *X. euvesicatoria.* The amino acid sequence of XpsC (accession number CAJ25390) and the positions of predicted α helices and β sheets predicted by AlphaFold2 are shown [41, 71|. Coloured rectangles refer to cytoplasmic, transmembrane and periplasmic regions as well as to the HR and 2P domains as indicated. Numbers indicate amino acid positions.

(B) Predicted secondary structure elements in GspC proteins. α helices and β sheets were predicted and indicated as described in (A). The following proteins were analysed: XcpP from *P. aeruginosa* strain PAO1 (accession number CAA48581), OutC from *D. dadantii* strain 3937 (accession number CAA46369) and EpsC from *V. cholerae* strain N16961 (accession number P45777).
