# Supplementary material for: Modularization of the type II secretion gene cluster from Xanthomonas euvesicatoria facilitates the identification of a structurally conserved XpsCLM assembly platform complex
Source: PLoS Pathog. 2025 Apr 9;21(4):e1013008. doi: 10.1371/journal.ppat.1013008 (PMC11981180; doi:10.1371/journal.ppat.1013008)
Supplement: S6 Fig — Complexes containing XpsCLM from X. euvesicatoria or corresponding proteins from P. aeruginosa, D. dadantii and V. cholerae were predicted using the AlphaFold2 algorithm and the molecular visualization program UCSF ChimeraX [40,41,71]. Similarly, XpsD as well as complexes between XpsL and XpsE, XpsC and the N-terminal region of XpsD (XpsD2-252) were modeled. In the models on the left side, proteins are shown in different colours as indicated. The models on the right side show the per-residue model confidence score (pLDDT, predicted local distance difference test) which is scaled from 0 to 100 with different colours referring to different scores as indicated. In addition, a predicted aligned error (PAE) plot shows regions of high (blue colour, low PAE value) and low (red colour, high PAE value) confidence for the predicted structures. pTM (predicted template modeling) and ipTM (inter-chain predicted template modeling) values are indicated. (PDF) [file ppat.1013008.s010.pdf]

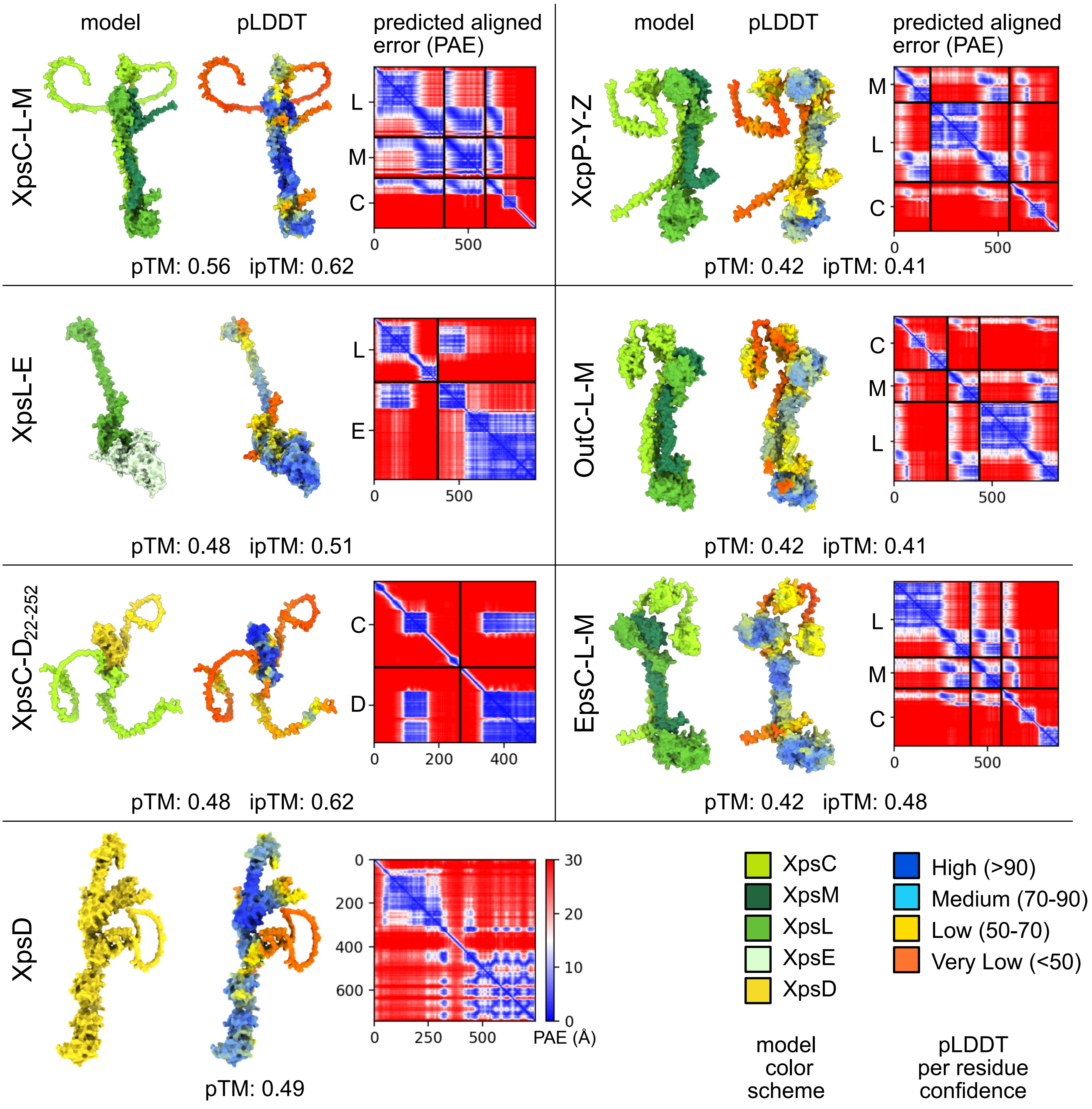

Supplemental figure 6

Goll *et al.*

Figure S6: Structure predictions of assembly platform components from *X. euvesicatoria, P. aeruginosa, D. dadantii* and *V. cholerae*.

Complexes containing XpsCLM from *X. euvesicatoria* or corresponding proteins from *P. aeruginosa, D. dadantii* and *V. cholerae* were predicted using the AlphaFold2 algorithm and the molecular visualization program UCSF ChimeraX [40, 41, 71|. Similarly, XpsD as well as complexes between XpsL and XpsE, XpsC and the N-terminal region of XpsD ($XpsD_{2-252}$) were modeled. In the models on the left side, proteins are shown in different colours as indicated. The models on the right side show the per-residue model confidence score (pLDDT, predicted local distance difference test) which is scaled from 0 to 100 with different colours referring to different scores as indicated. In addition, a predicted aligned error (PAE) plot shows regions of high (blue colour, low PAE value) and low (red colour, high PAE value) confidence for the predicted structures. pTM (predicted template modeling) and ipTM (inter-chain predicted template modeling) values are indicated.
