# Supplementary material for: Modularization of the type II secretion gene cluster from Xanthomonas euvesicatoria facilitates the identification of a structurally conserved XpsCLM assembly platform complex
Source: PLoS Pathog. 2025 Apr 9;21(4):e1013008. doi: 10.1371/journal.ppat.1013008 (PMC11981180; doi:10.1371/journal.ppat.1013008)
Supplement: S7 Fig — Cell extracts from E. coli strain JM109 containing expression constructs encoding T18 and T25 fusions of XpsC, XpsL, XpsM and XpsE as indicated were analysed by immunoblotting, using a FLAG epitope-specific antibody. (PDF) [file ppat.1013008.s011.pdf]

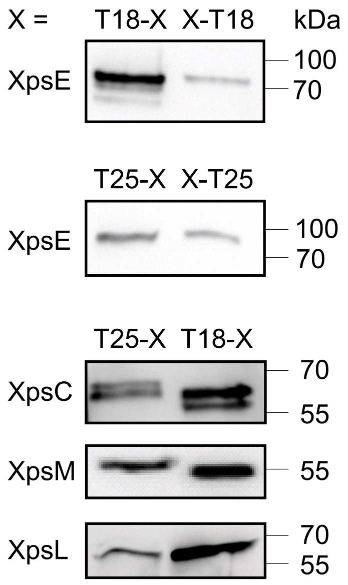

Supplemental figure 7

Goll *et al.*

Figure S7: Synthesis and immunological detection of T18 and T25 fusions of assembly platform components. Cell extracts from *E. coli* strain JM109 containing expression constructs encoding T18 and T25 fusions of XpsC, XpsL, XpsM and XpsE as indicated were analysed by immunoblotting, using a FLAG epitope-specific antibody.
