# Supplementary material for: Modularization of the type II secretion gene cluster from Xanthomonas euvesicatoria facilitates the identification of a structurally conserved XpsCLM assembly platform complex
Source: PLoS Pathog. 2025 Apr 9;21(4):e1013008. doi: 10.1371/journal.ppat.1013008 (PMC11981180; doi:10.1371/journal.ppat.1013008)
Supplement: S8 Fig — (A) The xpsG promoter, which was used for the expression of xpsC-c-myc, contains several potential ATG or GTG start codons as indicated. To prevent the synthesis of XpsC-c-Myc with additional N-terminal amino acids, the expression construct encoding c-Myc-XpsC,contained a nonsense mutation (C to T exchange) upstream of the translation initiation start site as indicated. The mutated nucleotide is underlined, the start codon of c-myc-xpsC in shown in bold letters. (B) Detection of c-Myc-XpsC-containing complexes after in vivo crosslinking using the expression construct shown in (A). Derivatives of strain 85-10∆xps (∆xps) containing the wild-type (WT) modular T2S gene cluster (+pT2S) or a derivative thereof deleted in xpsC and encoding c-Myc-XpsC as indicated were grown in NYG medium. Equal amounts of cell cultures were centrifuged and cells were either resuspended in Laemmli buffer at 99°C (total extract) or incubated with formaldehyde and resuspended in Laemmli buffer at 37°C (crosslinked). Proteins were analysed by immunoblotting using antibodies specific for the c-Myc epitope or GroEL to ensure equal loading. Signals corresponding to c-Myc-XpsC and a c-Myc-XpsC-specific protein complex are indicated with a black arrow and an asterisk, respectively. For the analysis of T2S system activity, bacteria were grown on milk protein-containing agar plates to demonstrate extracellular protease activity. Halo formation was documented two days after incubation. Experiments were performed three times with similar results. One representative example is shown. The numbers refer to the width of the halos in millimeters, with mean values calculated from three replicates. (PDF) [file ppat.1013008.s012.pdf]

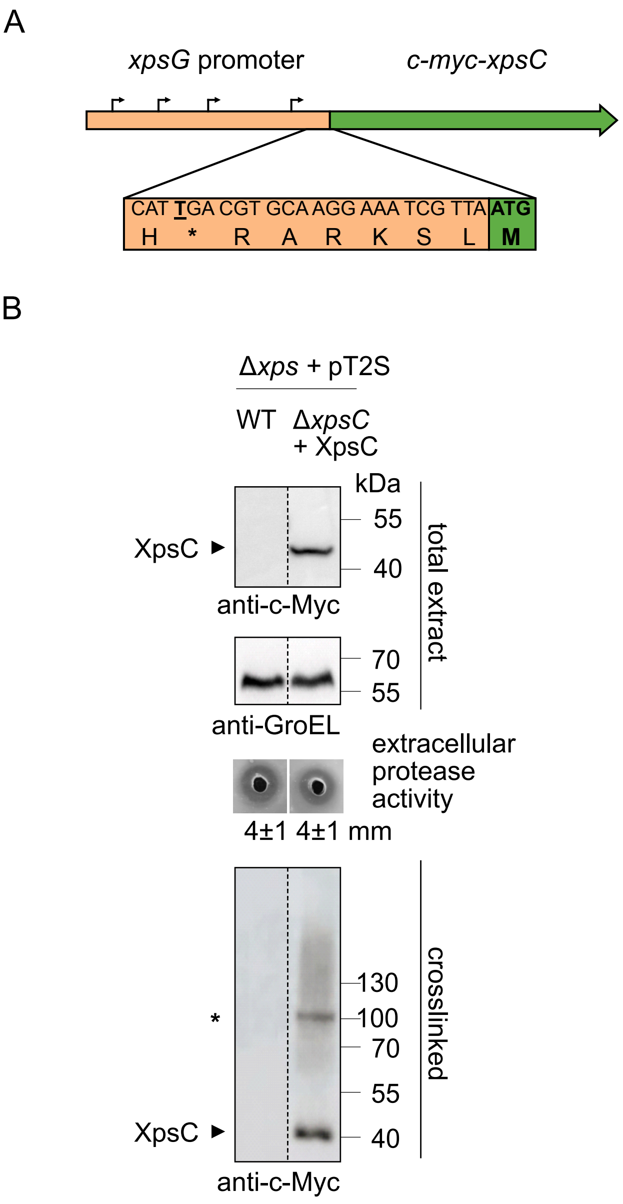

Supplemental figure 8

Goll *et al.*

Figure S8: Introduction of a nonsense mutation upstream of the start codon of *xpsC* prevents alternative translation initiation.

(A) The *xpsG* promoter, which was used for the expression of *xpsC-c-myc*, contains several potential ATG or GTG start codons as indicated. To prevent the synthesis of XpsC-c-Myc with additional N-terminal amino acids, the expression construct encoding c-Myc-XpsC,contained a nonsense mutation (C to T exchange) upstream of the translation initiation start site as indicated. The mutated nucleotide is underlined, the start codon of *c-myc-xpsC* in shown in bold letters.

(B) Detection of c-Myc-XpsC-containing complexes after *in vivo* crosslinking using the expression construct shown in (A). Derivatives of strain 85-10Δ*xps* (Δ*xps*) containing the wild-type (WT) modular T2S gene cluster (+pT2S) or a derivative thereof deleted in *xpsC* and encoding c-Myc-XpsC as indicated were grown in NYG medium. Equal amounts of cell cultures were centrifuged and cells were either resuspended in Laemmli buffer at 99°C (total extract) or incubated with formaldehyde and resuspended in Laemmli buffer at 37°C (crosslinked). Proteins were analysed by immunoblotting using antibodies specific for the c-Myc epitope or GroEL to ensure equal loading. Signals corresponding to c-Myc-XpsC and a c-Myc-XpsC-specific protein complex are indicated with a black arrow and an asterisk, respectively. For the analysis of T2S system activity, bacteria were grown on milk protein-containing agar plates to demonstrate extracellular protease activity. Halo formation was documented two days after incubation. Experiments were performed three times with similar results. One representative example is shown. The numbers refer to the width of the halos in millimeters, with mean values calculated from three replicates.
