# Supplementary material for: Modularization of the type II secretion gene cluster from Xanthomonas euvesicatoria facilitates the identification of a structurally conserved XpsCLM assembly platform complex
Source: PLoS Pathog. 2025 Apr 9;21(4):e1013008. doi: 10.1371/journal.ppat.1013008 (PMC11981180; doi:10.1371/journal.ppat.1013008)
Supplement: S9 Fig — (A) Complementation studies with XpsC-c-Myc. Xe strains 85-10 (WT) and 85-10∆xpsD (∆xpsD) with or without an expression construct encoding XpsD-c-Myc under control of the XCV4361 promoter were inoculated into leaves of susceptible ECW (Early Cal Wonder) pepper plants. Disease symptoms were photographed 7 dpi. Dashed lines indicate the infiltrated areas. For the analysis of extracellular protease activity, bacteria were grown on milk protein-containing agar plates and halo formation was documented two days after incubation. Experiments were performed three times with similar results. When compared with the lac promoter, the XCV4361 promoter results in lower expression levels [30,72]. (B) XpsC-c-Myc-specific complexes are detected in the absence of other T2S system components. Strains 85-10 (WT), 85-10∆xpsE-D (∆xpsE-D) and 85-10∆xpsD (∆xpsD) containing the XpsD-c-Myc expression construct (XpsD) as indicated were grown in minimal medium at pH 7.0. Equal amounts of bacterial cultures during the exponential growth phase were analysed by immunoblotting using a c-Myc epitope-specific antibody. The blot was reprobed with an antibody against GroEL to demonstrate equal loading. XpsD-c-Myc-specific signals, which likely correspond to oligomeric complexes, were detected in the stacking gel as indicated. (PDF) [file ppat.1013008.s013.pdf]

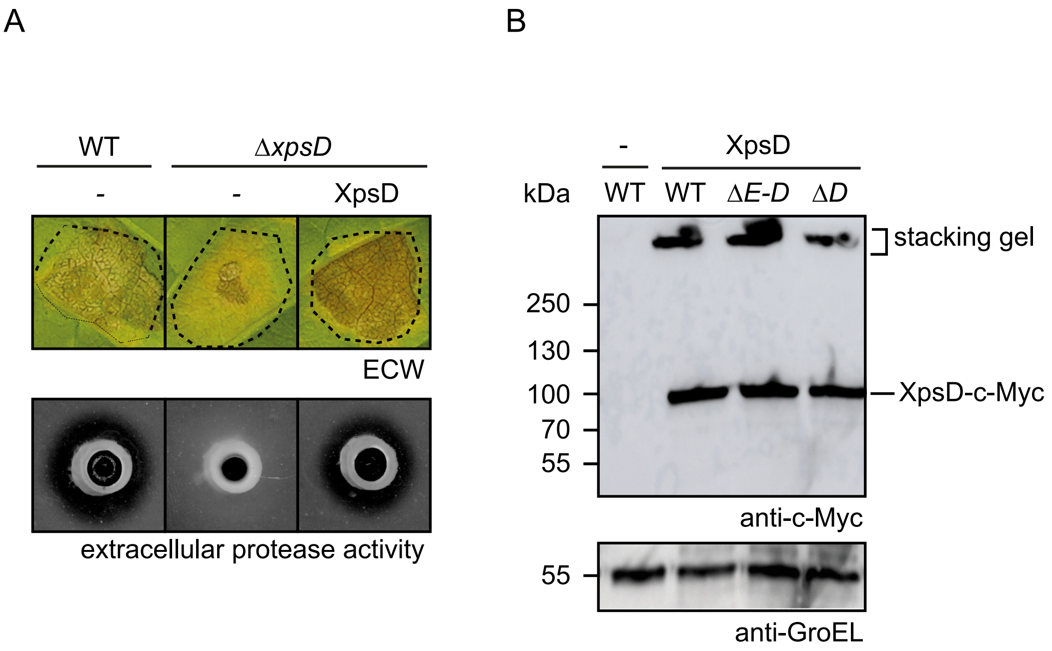

Supplemental figure 9

Goll *et al.*

Figure S9 A C-terminally c-Myc epitope-tagged XpsD derivative is functional and forms protein complexes.

(A) Complementation studies with XpsC-c-Myc. *Xe* strains 85-10 (WT) and 85-10Δ*xpsD* (Δ*xpsD*) with or without an expression construct encoding XpsD-c-Myc under control of the *XCV4361* promoter were inoculated into leaves of susceptible ECW (Early Cal Wonder) pepper plants. Disease symptoms were photographed 7 dpi. Dashed lines indicate the infiltrated areas. For the analysis of extracellular protease activity, bacteria were grown on milk protein-containing agar plates and halo formation was documented two days after incubation. Experiments were performed three times with similar results. When compared with the *lac* promoter, the *XCV4361* promoter results in lower expression levels [30, 72].

(B) XpsC-c-Myc-specific complexes are detected in the absence of other T2S system components. Strains 85-10 (WT), 85-10Δ*xpsE-D* (Δ*xpsE-D*) and 85-10Δ*xpsD* (Δ*xpsD*) containing the XpsD-c-Myc expression construct (XpsD) as indicated were grown in minimal medium at pH 7.0. Equal amounts of bacterial cultures during the exponential growth phase were analysed by immunoblotting using a c-Myc epitope-specific antibody. The blot was reprobed with an antibody against GroEL to demonstrate equal loading. XpsD-c-Myc-specific signals, which likely correspond to oligomeric complexes, were detected in the stacking gel as indicated.
