# Supplementary material for: Modularization of the type II secretion gene cluster from Xanthomonas euvesicatoria facilitates the identification of a structurally conserved XpsCLM assembly platform complex
Source: PLoS Pathog. 2025 Apr 9;21(4):e1013008. doi: 10.1371/journal.ppat.1013008 (PMC11981180; doi:10.1371/journal.ppat.1013008)
Supplement: S10 Fig — X. euvesicatoria strain 85*∆hrp_fsHAGX containing a modular T3S gene cluster encoding the type III effector XopB fused to mCherry was incubated in minimal medium under T3S-permissive conditions. mCherry fluorescence was analysed by fluorescence microscopy. One representative image is shown. The size bar corresponds to 2.5 µm. The picture in the right panel results from an overlay of the fluorescent signals with the images of the brightfield channel. The modular T3S gene cluster was previously generated using Golden Gate cloning and contains the hrp (hypersensitive response and pathogenicity) gene cluster and the accessory genes xopA, hpaH, hrpG and hrpX. A reporter fusion encoding, e.g., XopB-mCherry was included. The modular T3S gene cluster construct was analysed in a X. euvesicatoria strain deleted in the native hrp gene cluster (∆hrp) and containing frameshift (fs) mutations in xopA, hpaH, hrpG and hrpX (HAGX) [36]. XopB-mCherry does not form fluorescent foci and is detected in the bacterial cytoplasm. The cytoplasmic localization of mCherry was previously also reported for other Xanthomonas spp. [73]. (PDF) [file ppat.1013008.s014.pdf]

XopB-mCherry

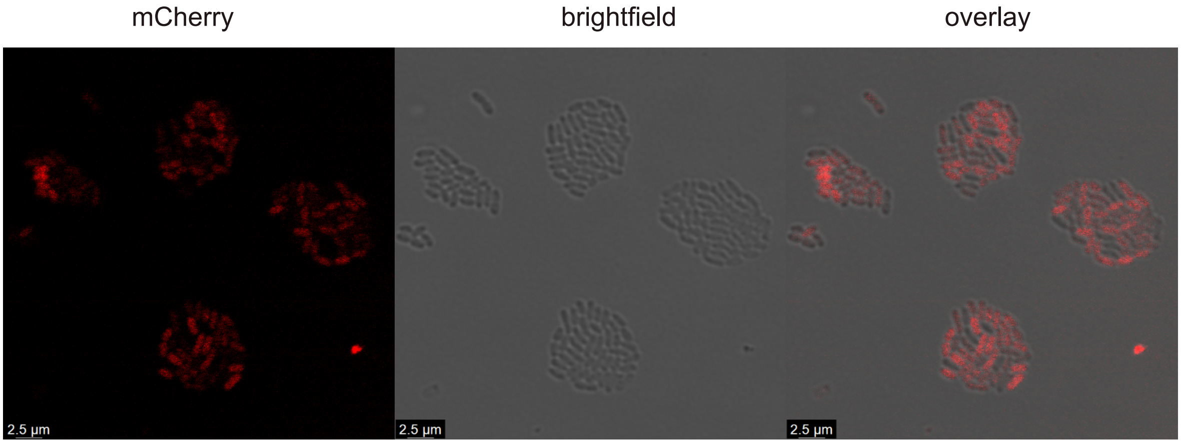

Supplemental figure 10

Goll *et al.*

Figure S10 Localization of XopB-mCherry in *X. euvesicatoria.*

X. euvesicatoria strain 85*Δ*hrp*_fs*HAGX* containing a modular T3S gene cluster encoding the type III effector XopB fused to mCherry was incubated in minimal medium under T3S-permissive conditions. mCherry fluorescence was analysed by fluorescence microscopy. One representative image is shown. The size bar corresponds to 2.5 μm. The picture in the right panel results from an overlay of the fluorescent signals with the images of the brightfield channel. The modular T3S gene cluster was previously generated using Golden Gate cloning and contains the *hrp* (hypersensitive response and pathogenicity) gene cluster and the accessory genes *xopA, hpaH, hrpG* and *hrpX.* A reporter fusion encoding e.g. XopB-mCherry was included. The modular T3S gene cluster construct was analysed in a *X. euvesicatoria* strain deleted in the native *hrp* gene cluster (Δ*hrp*) and containing frameshift (fs) mutations in *xopA, hpaH, hrpG* and *hrpX* (*HAGX*) [36]. XopB-mCherry does not form fluorescent foci and is detected in the bacterial cytoplasm. The cytoplasmic localization of mCherry was previously also reported for other *Xanthomonas* spp. [73].
